# Supplementary figures and images for: Unconventional secretion of α-synuclein mediated by palmitoylated DNAJC5 oligomers (part 1 of 2)
Source: eLife. 2023 Jan 10;12:e85837. doi: 10.7554/eLife.85837 (PMC9876576; doi:10.7554/eLife.85837)

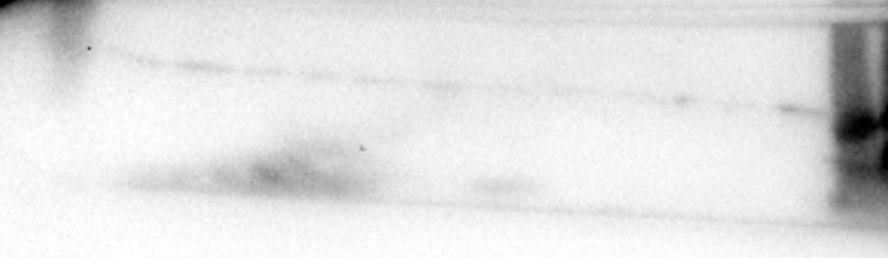

Supplement: Figure 1—source data 1. [file elife-85837-fig1-data1.zip › Figure 1-source data/Figure 1D-1.tif]

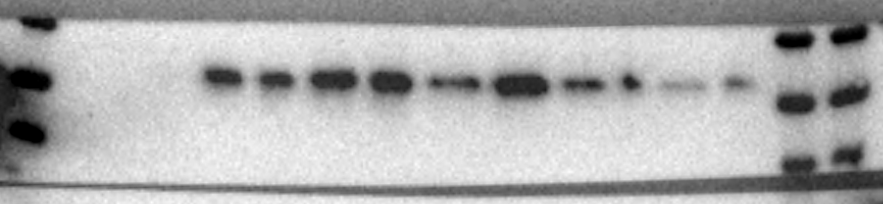

Supplement: Figure 1—source data 1. [file elife-85837-fig1-data1.zip › Figure 1-source data/Figure 1D-3.tif]

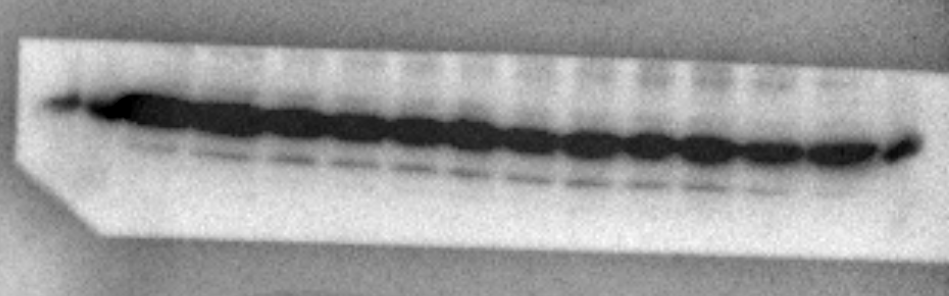

Supplement: Figure 1—source data 1. [file elife-85837-fig1-data1.zip › Figure 1-source data/Figure 1D-2.tif]

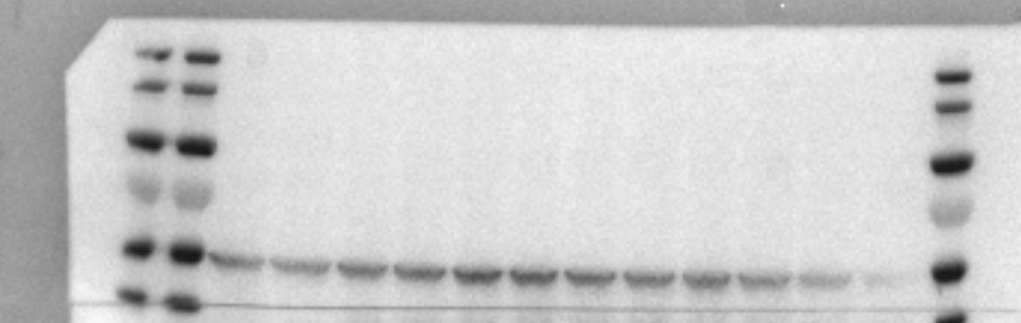

Supplement: Figure 1—source data 1. [file elife-85837-fig1-data1.zip › Figure 1-source data/Figure 1D-6.tif]

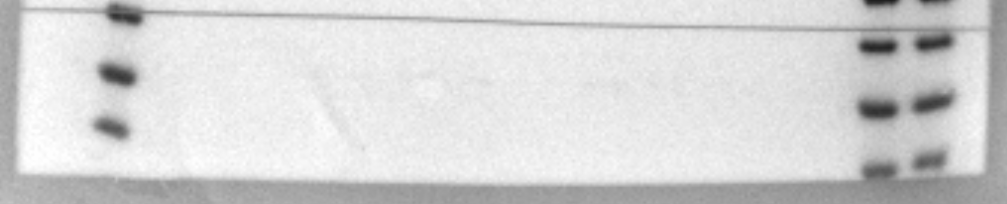

Supplement: Figure 1—source data 1. [file elife-85837-fig1-data1.zip › Figure 1-source data/Figure 1D-5.tif]

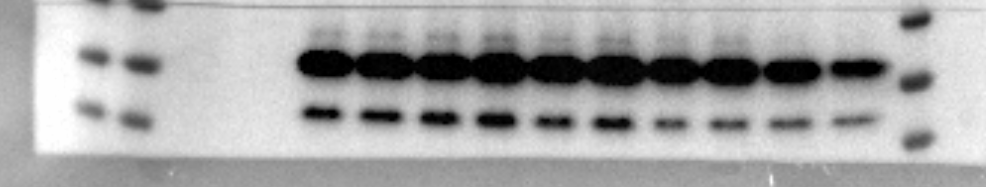

Supplement: Figure 1—source data 1. [file elife-85837-fig1-data1.zip › Figure 1-source data/Figure 1D-4.tif]

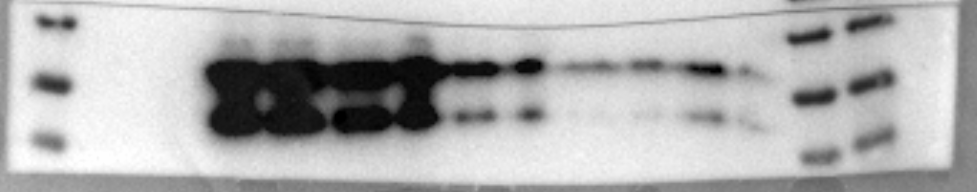

Supplement: Figure 1—source data 1. [file elife-85837-fig1-data1.zip › Figure 1-source data/Figure 1E-3.tif]

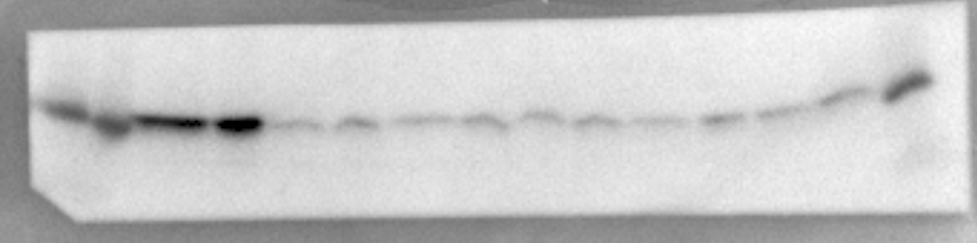

Supplement: Figure 1—source data 1. [file elife-85837-fig1-data1.zip › Figure 1-source data/Figure 1E-2.tif]

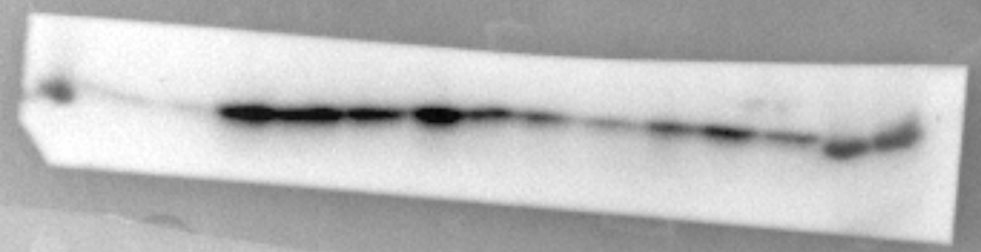

Supplement: Figure 1—source data 1. [file elife-85837-fig1-data1.zip › Figure 1-source data/Figure 1E-1.tif]

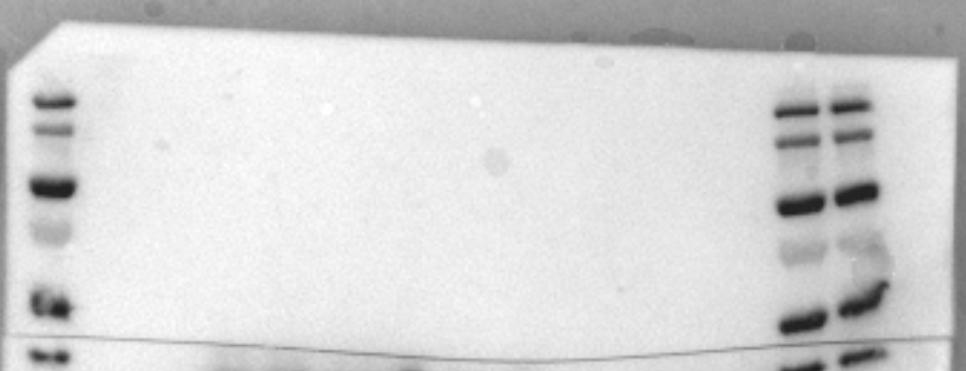

Supplement: Figure 1—source data 1. [file elife-85837-fig1-data1.zip › Figure 1-source data/Figure 1E-5.tif]

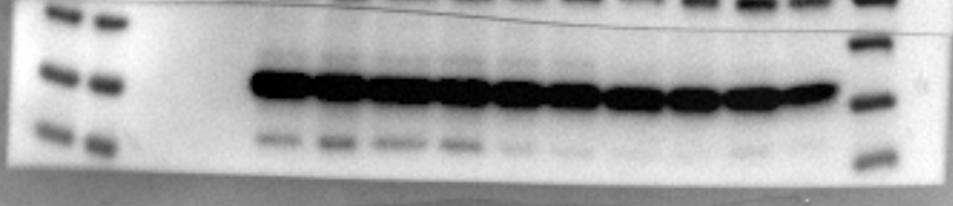

Supplement: Figure 1—source data 1. [file elife-85837-fig1-data1.zip › Figure 1-source data/Figure 1E-4.tif]

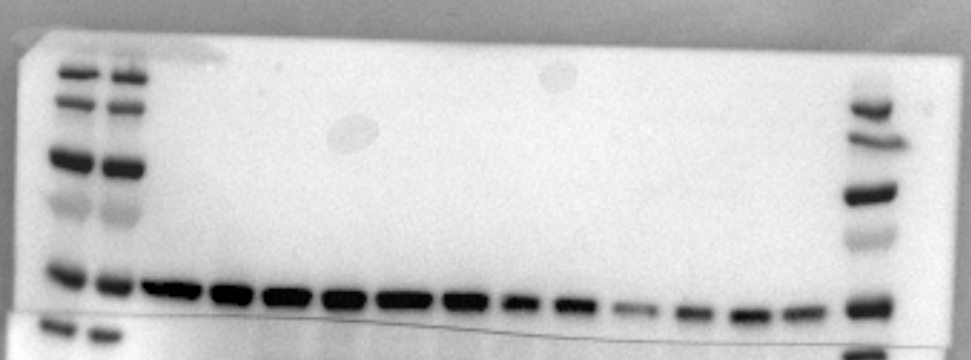

Supplement: Figure 1—source data 1. [file elife-85837-fig1-data1.zip › Figure 1-source data/Figure 1E-6.tif]

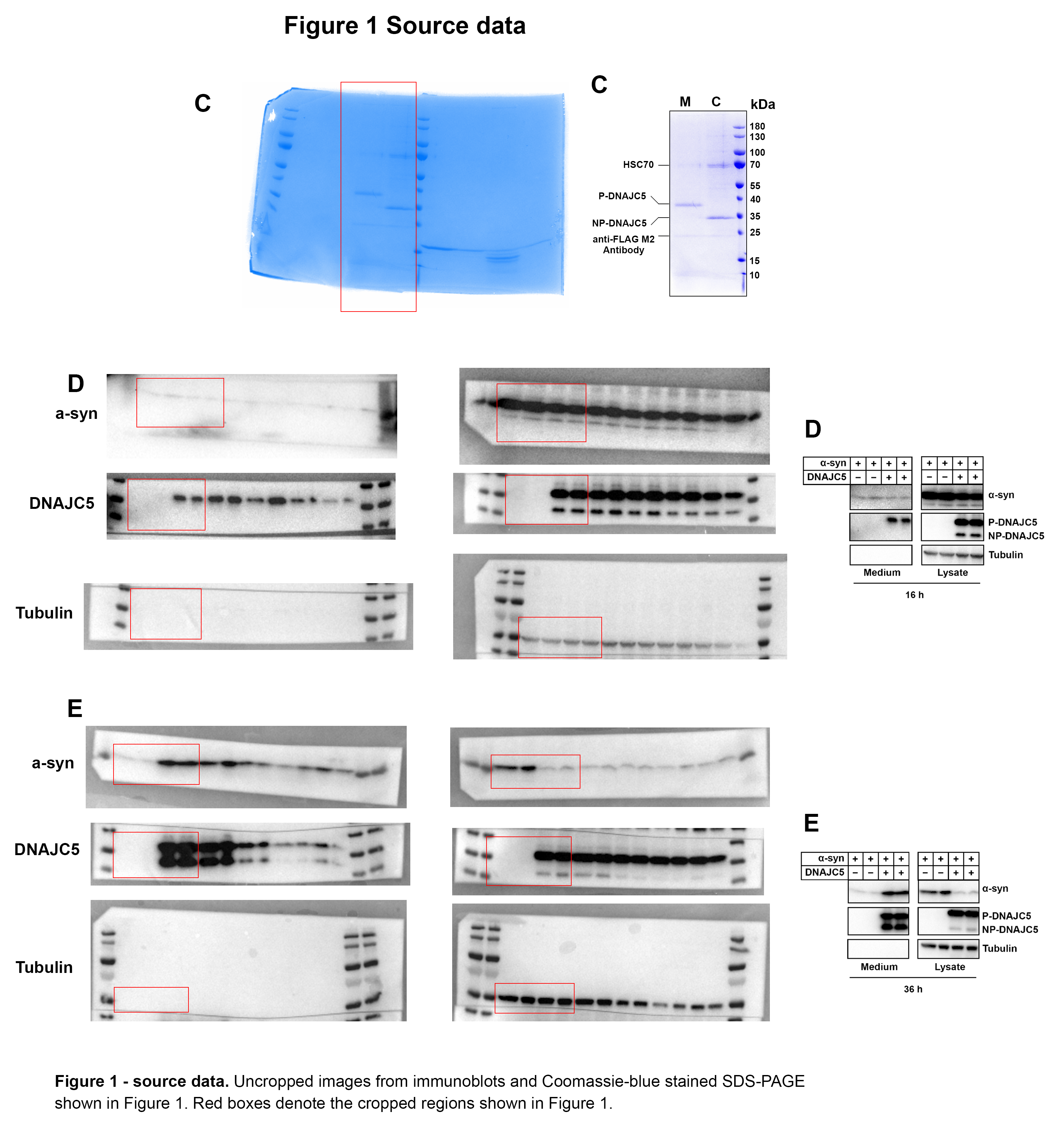

Supplement: Figure 1—source data 1. [file elife-85837-fig1-data1.zip › Figure 1-source data/Figure 1-source data.tif]

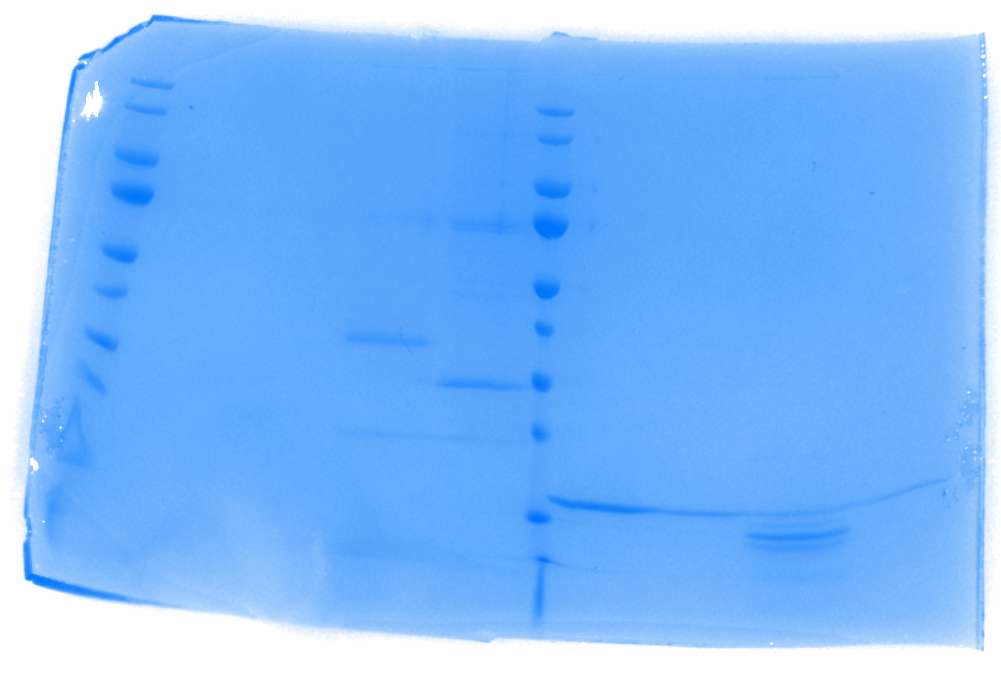

Supplement: Figure 1—source data 1. [file elife-85837-fig1-data1.zip › Figure 1-source data/Figure 1C.tif]

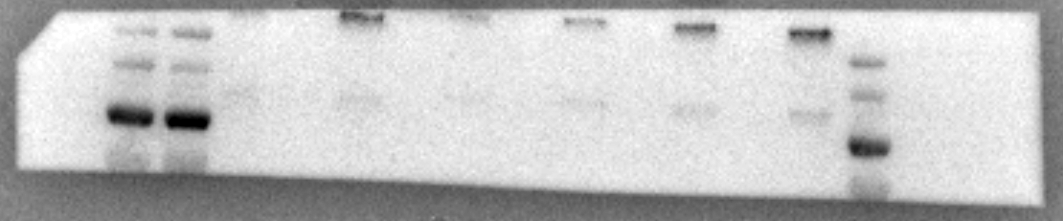

Supplement: Figure 1—figure supplement 1—source data 1. [file elife-85837-fig1-figsupp1-data1.zip › Figure 1-figure supplement 1-source data/Figure 1- figure supplement 1A-2.tif]

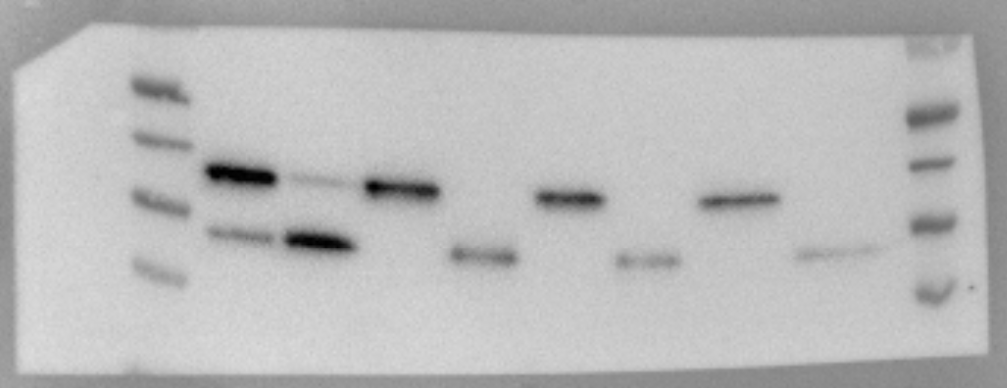

Supplement: Figure 1—figure supplement 1—source data 1. [file elife-85837-fig1-figsupp1-data1.zip › Figure 1-figure supplement 1-source data/Figure 1- figure supplement 1B.tif]

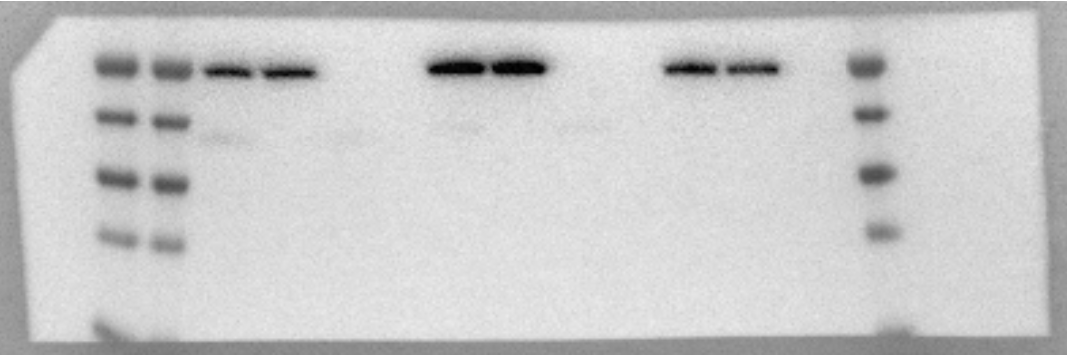

Supplement: Figure 1—figure supplement 1—source data 1. [file elife-85837-fig1-figsupp1-data1.zip › Figure 1-figure supplement 1-source data/Figure 1- figure supplement 1A-3.tif]

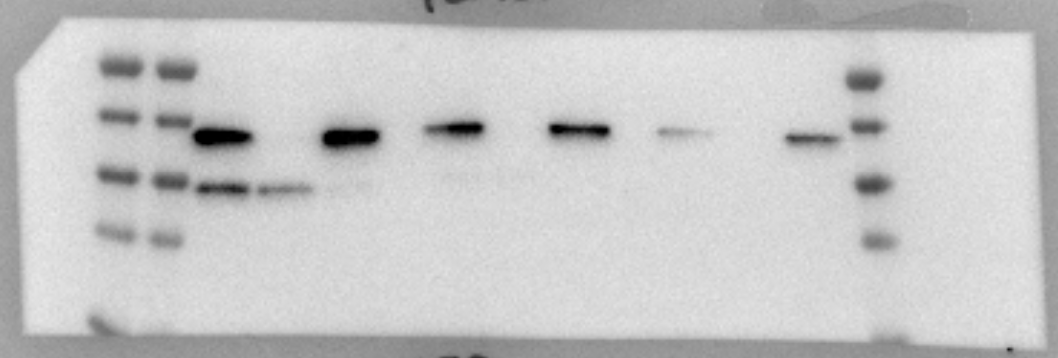

Supplement: Figure 1—figure supplement 1—source data 1. [file elife-85837-fig1-figsupp1-data1.zip › Figure 1-figure supplement 1-source data/Figure 1- figure supplement 1A-1.tif]

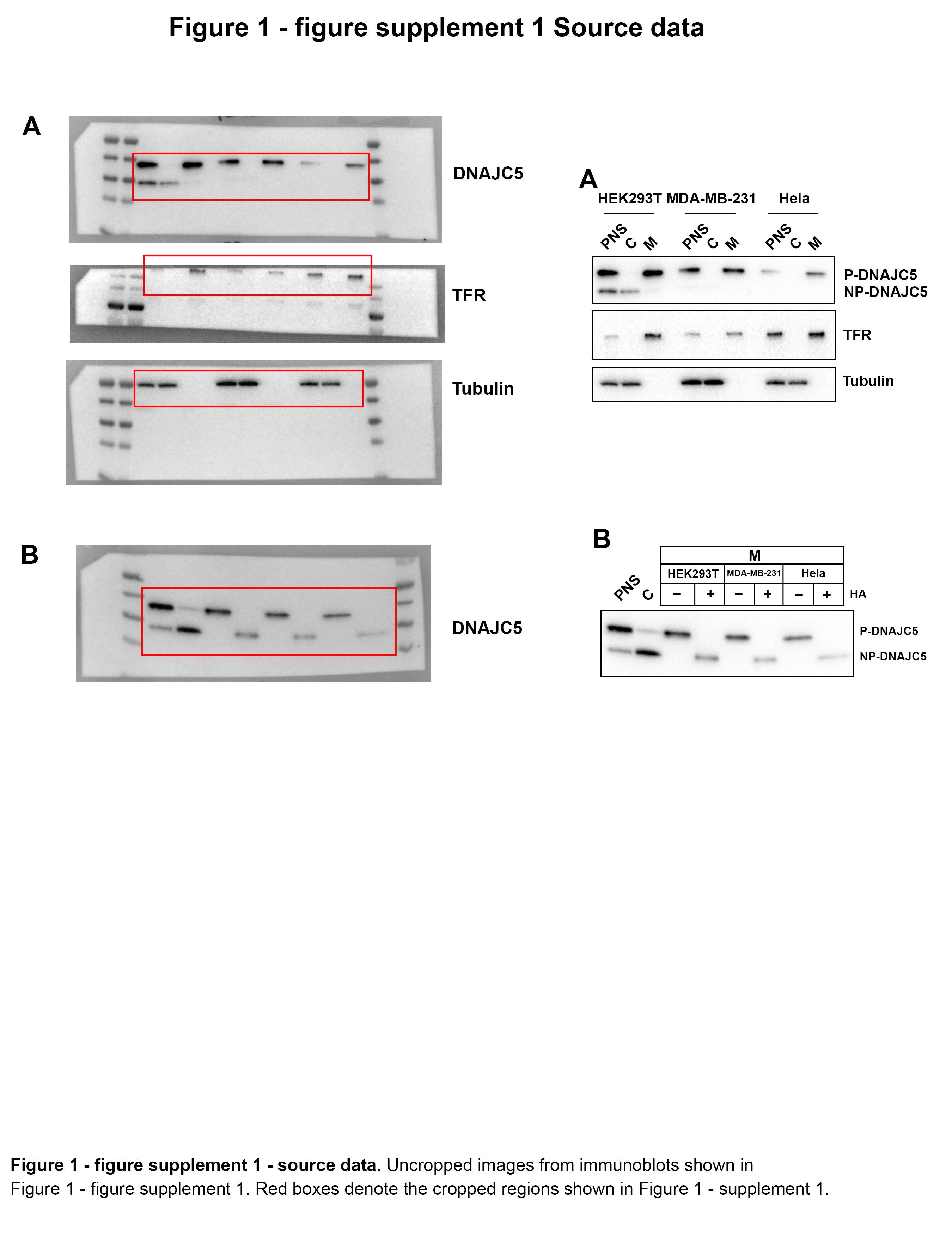

Supplement: Figure 1—figure supplement 1—source data 1. [file elife-85837-fig1-figsupp1-data1.zip › Figure 1-figure supplement 1-source data/Figure 1- figure supplement 1-source data.tif]

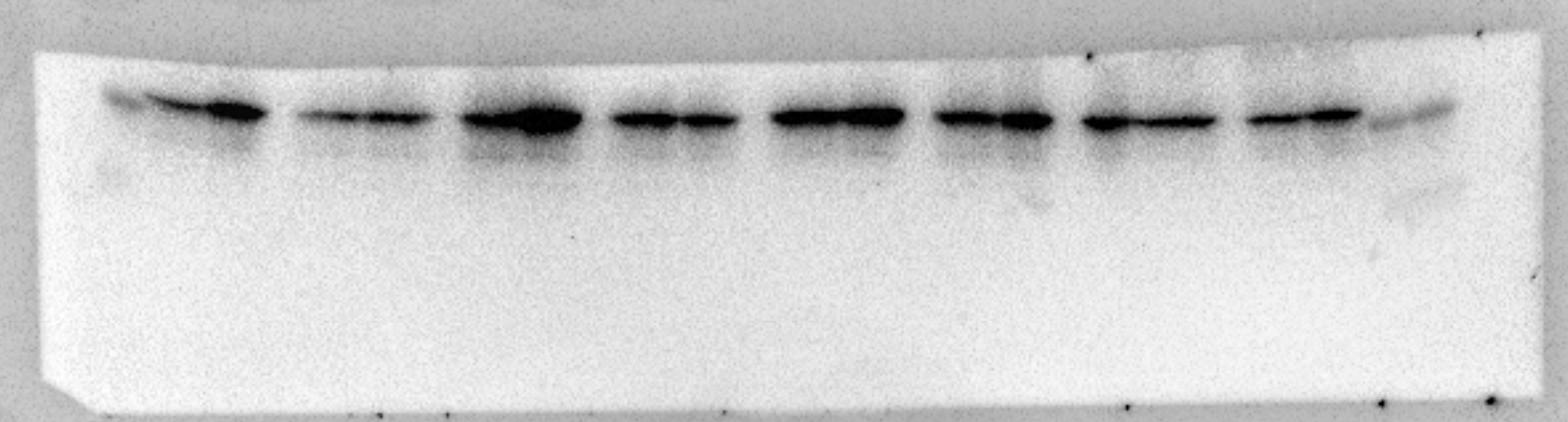

Supplement: Figure 1—figure supplement 2—source data 1. [file elife-85837-fig1-figsupp2-data1.zip › Figure 1-figure supplement 2-source data/Figure 1- figure supplement 2C-1.tif]

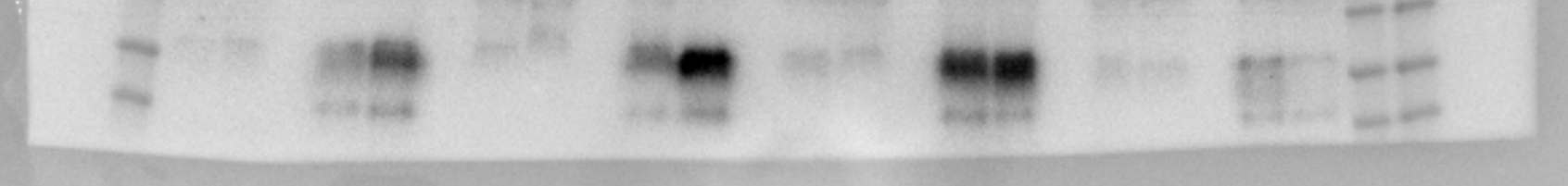

Supplement: Figure 1—figure supplement 2—source data 1. [file elife-85837-fig1-figsupp2-data1.zip › Figure 1-figure supplement 2-source data/Figure 1- figure supplement 2C-2.tif]

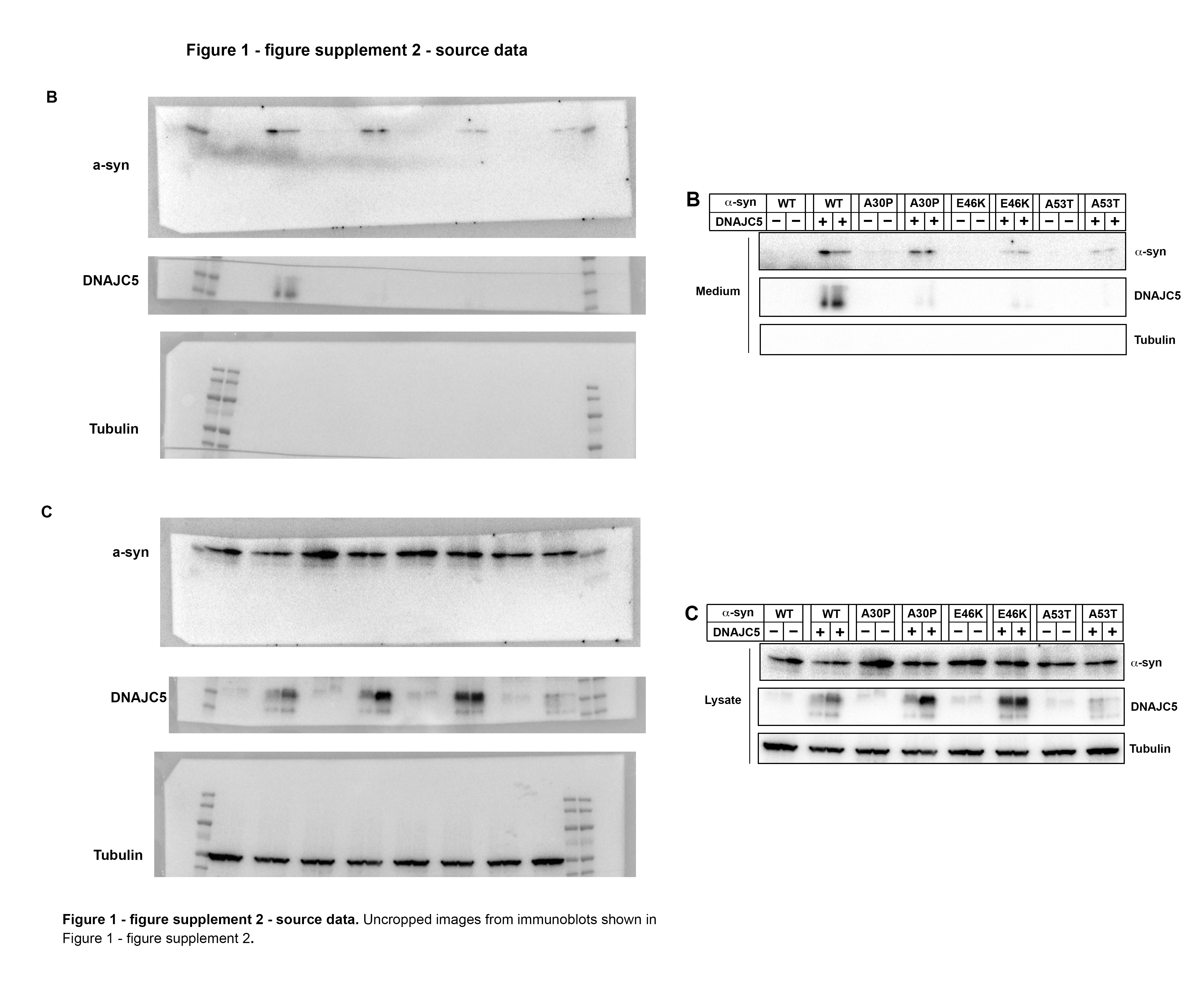

Supplement: Figure 1—figure supplement 2—source data 1. [file elife-85837-fig1-figsupp2-data1.zip › Figure 1-figure supplement 2-source data/Figure 1- figure supplement 2-source data.tif]

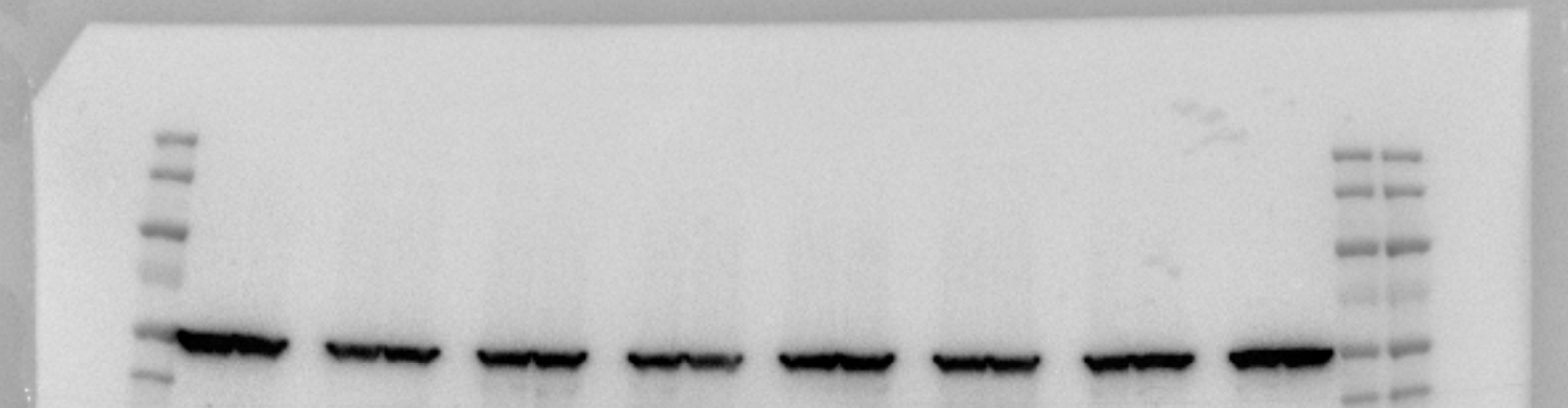

Supplement: Figure 1—figure supplement 2—source data 1. [file elife-85837-fig1-figsupp2-data1.zip › Figure 1-figure supplement 2-source data/Figure 1- figure supplement 2C-3.tif]

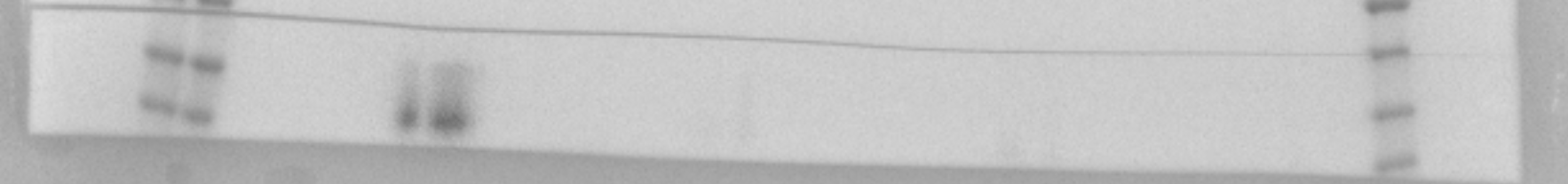

Supplement: Figure 1—figure supplement 2—source data 1. [file elife-85837-fig1-figsupp2-data1.zip › Figure 1-figure supplement 2-source data/Figure 1- figure supplement 2B-2.tif]

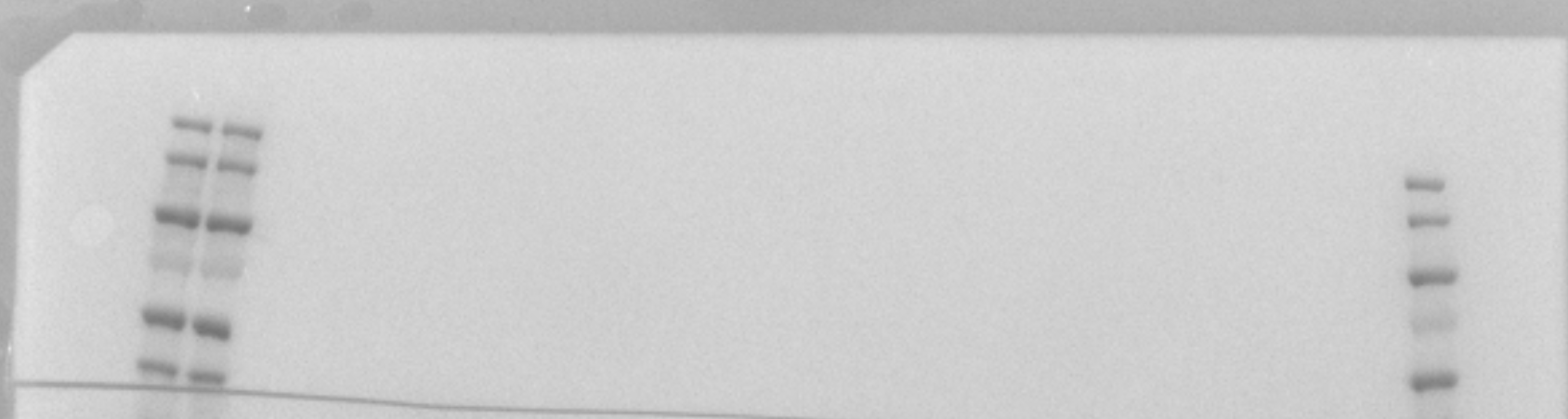

Supplement: Figure 1—figure supplement 2—source data 1. [file elife-85837-fig1-figsupp2-data1.zip › Figure 1-figure supplement 2-source data/Figure 1- figure supplement 2B-3.tif]

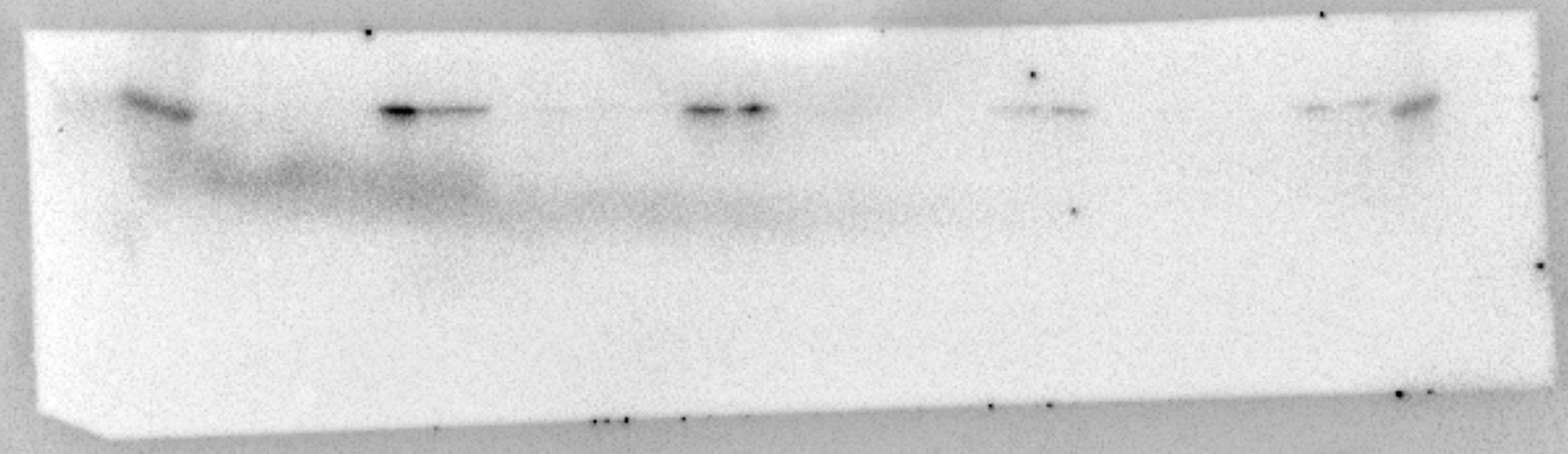

Supplement: Figure 1—figure supplement 2—source data 1. [file elife-85837-fig1-figsupp2-data1.zip › Figure 1-figure supplement 2-source data/Figure 1- figure supplement 2B-1.tif]

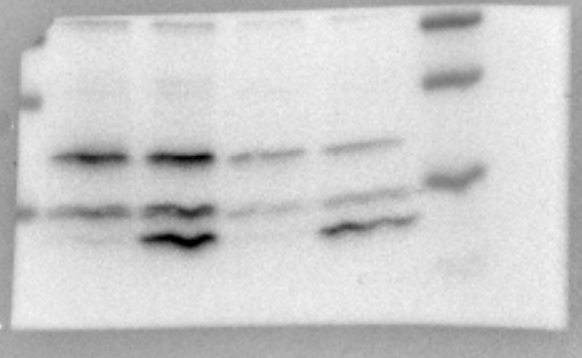

Supplement: Figure 1—figure supplement 3—source data 1. [file elife-85837-fig1-figsupp3-data1.zip › Figure 1-figure supplement 3-source data/Figure 1- figure supplement 3G-1.tif]

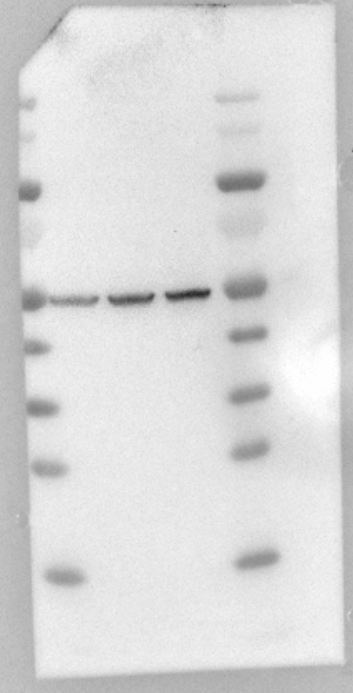

Supplement: Figure 1—figure supplement 3—source data 1. [file elife-85837-fig1-figsupp3-data1.zip › Figure 1-figure supplement 3-source data/Figure 1- figure supplement 3E-3.tif]

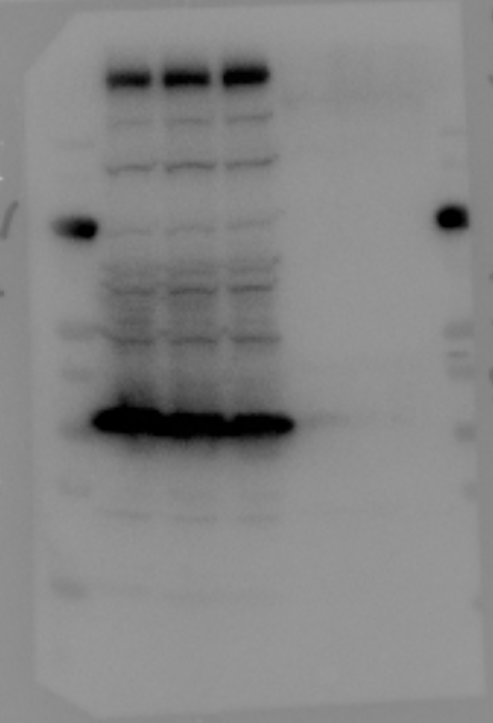

Supplement: Figure 1—figure supplement 3—source data 1. [file elife-85837-fig1-figsupp3-data1.zip › Figure 1-figure supplement 3-source data/Figure 1- figure supplement 3E-2.tif]

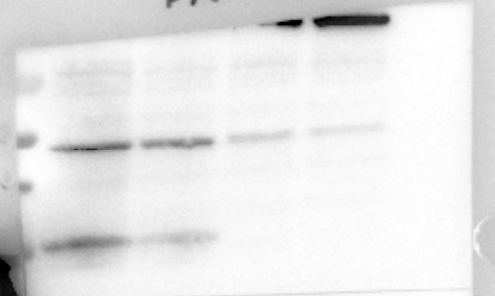

Supplement: Figure 1—figure supplement 3—source data 1. [file elife-85837-fig1-figsupp3-data1.zip › Figure 1-figure supplement 3-source data/Figure 1- figure supplement 3G-2.tif]

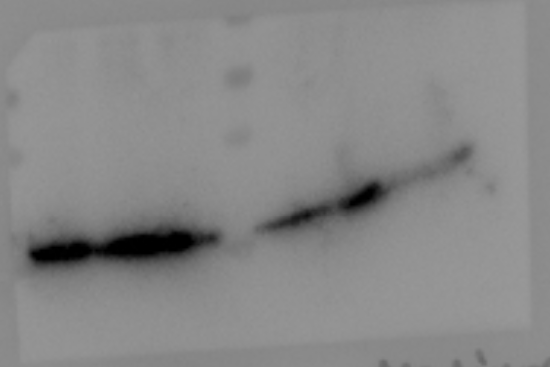

Supplement: Figure 1—figure supplement 3—source data 1. [file elife-85837-fig1-figsupp3-data1.zip › Figure 1-figure supplement 3-source data/Figure 1- figure supplement 3E-1.tif]

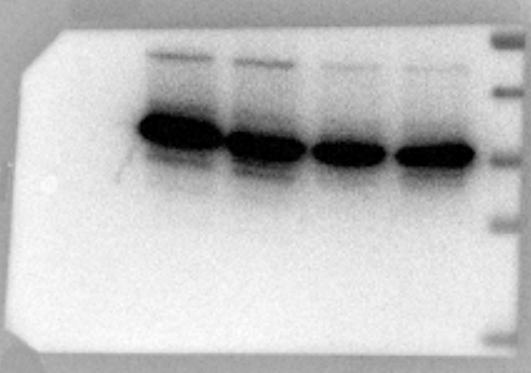

Supplement: Figure 1—figure supplement 3—source data 1. [file elife-85837-fig1-figsupp3-data1.zip › Figure 1-figure supplement 3-source data/Figure 1- figure supplement 3G-3.tif]

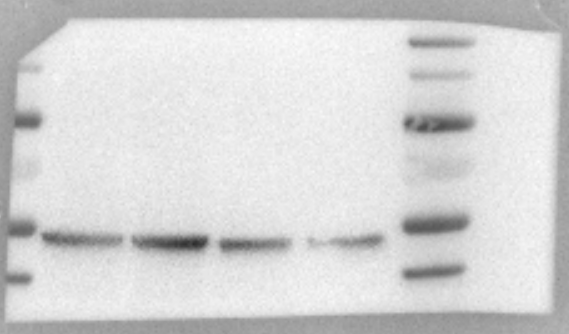

Supplement: Figure 1—figure supplement 3—source data 1. [file elife-85837-fig1-figsupp3-data1.zip › Figure 1-figure supplement 3-source data/Figure 1- figure supplement 3G-4.tif]

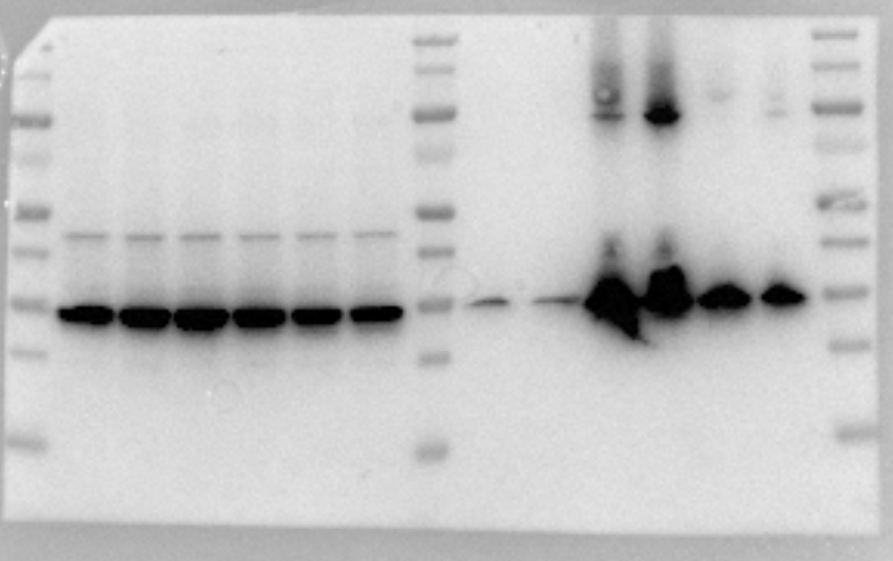

Supplement: Figure 1—figure supplement 3—source data 1. [file elife-85837-fig1-figsupp3-data1.zip › Figure 1-figure supplement 3-source data/Figure 1- figure supplement 3B-1.tif]

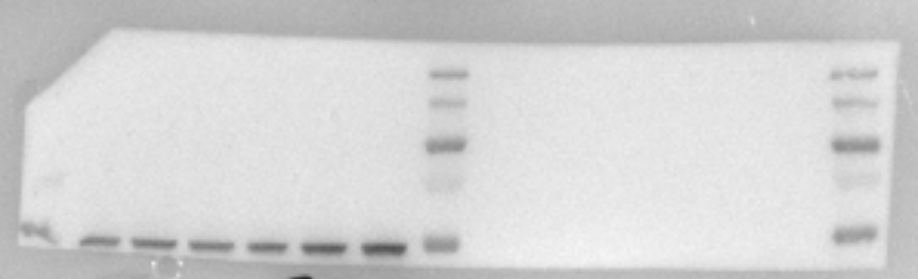

Supplement: Figure 1—figure supplement 3—source data 1. [file elife-85837-fig1-figsupp3-data1.zip › Figure 1-figure supplement 3-source data/Figure 1- figure supplement 3B-3.tif]

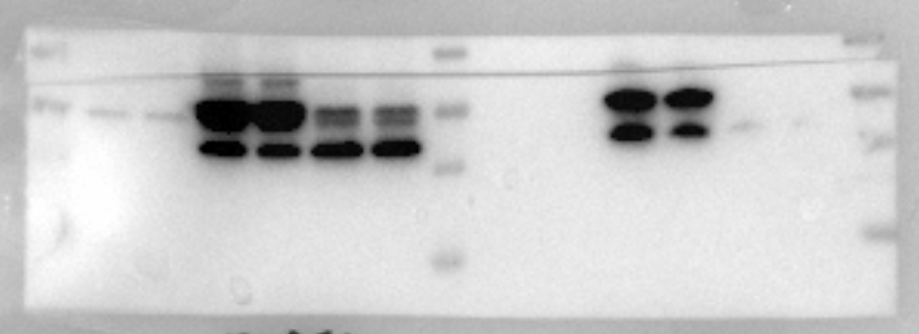

Supplement: Figure 1—figure supplement 3—source data 1. [file elife-85837-fig1-figsupp3-data1.zip › Figure 1-figure supplement 3-source data/Figure 1- figure supplement 3B-2.tif]

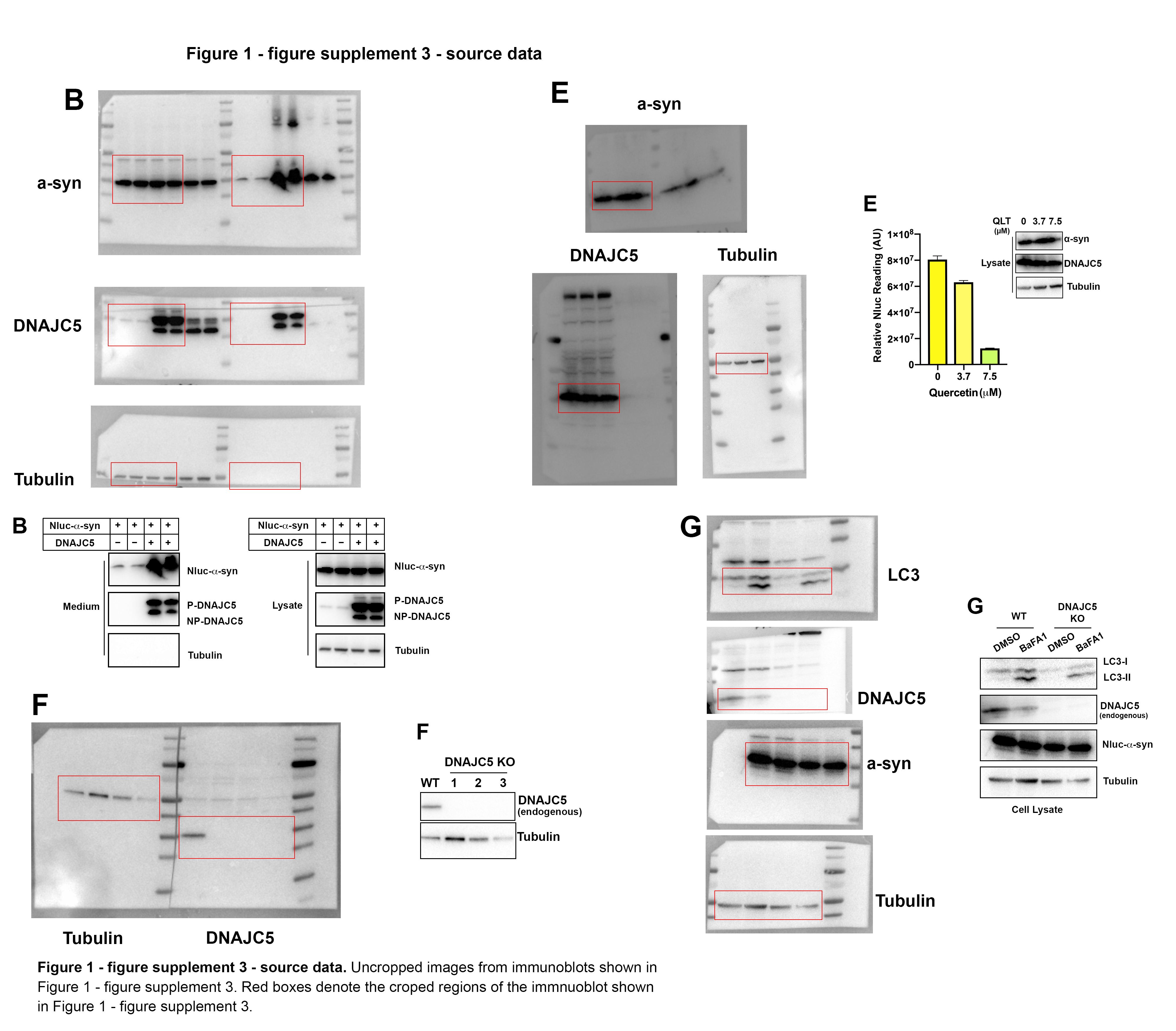

Supplement: Figure 1—figure supplement 3—source data 1. [file elife-85837-fig1-figsupp3-data1.zip › Figure 1-figure supplement 3-source data/Figure 1- figure supplement 3-source data.tif]

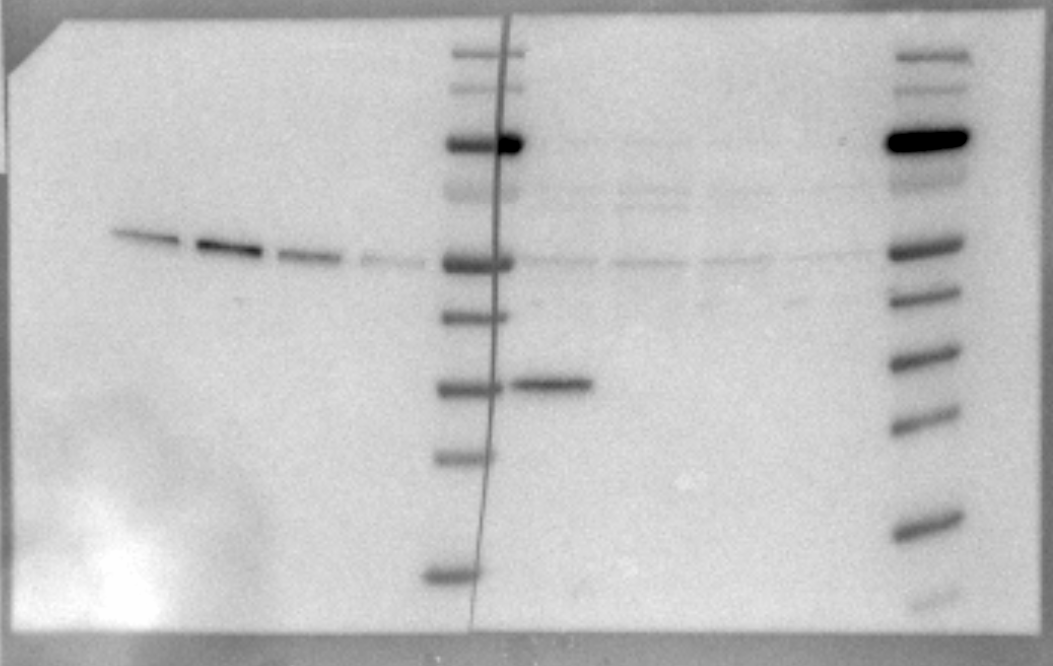

Supplement: Figure 1—figure supplement 3—source data 1. [file elife-85837-fig1-figsupp3-data1.zip › Figure 1-figure supplement 3-source data/Figure 1- figure supplement 3F.tif]

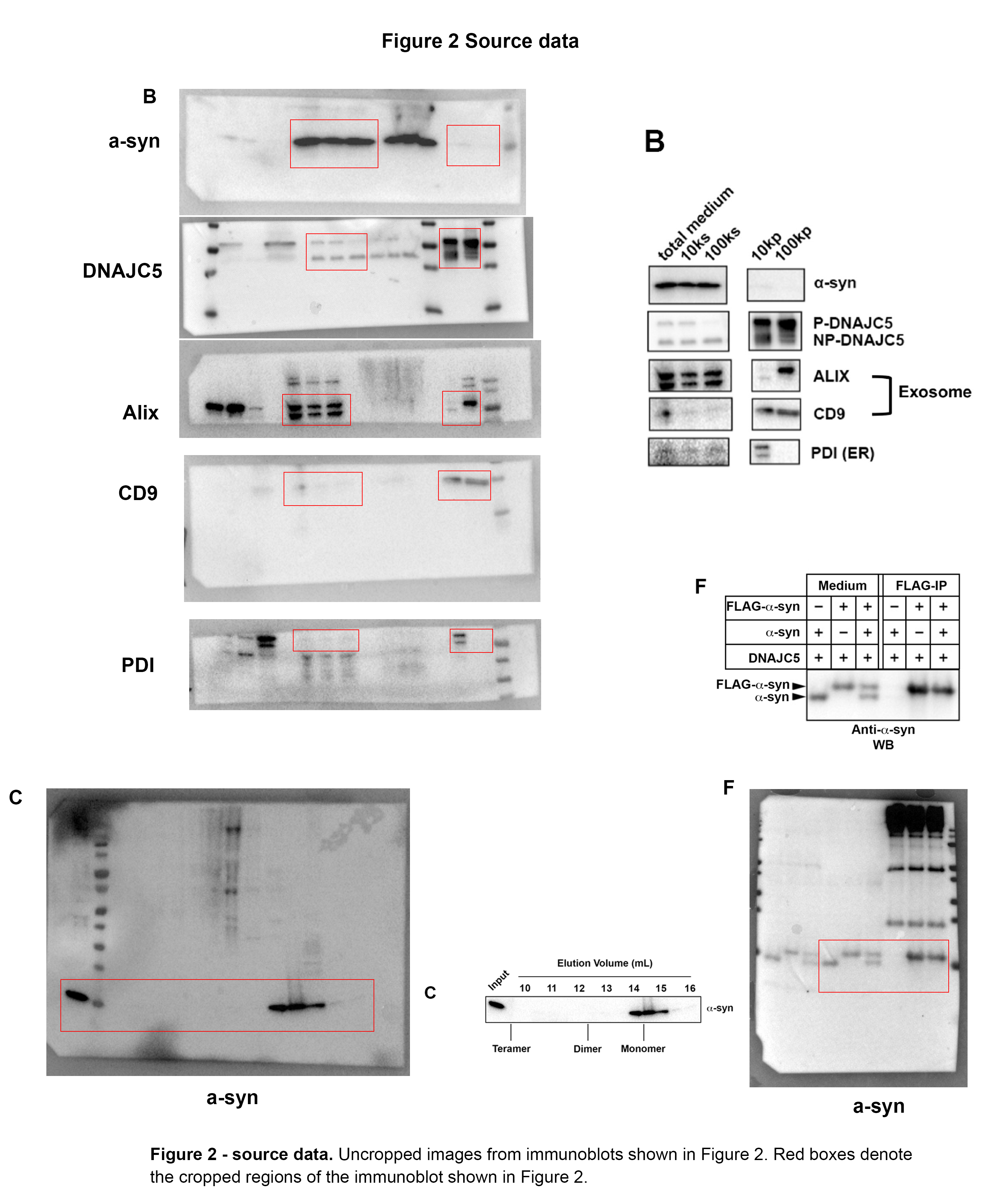

Supplement: Figure 2—source data 1. [file elife-85837-fig2-data1.zip › Figure 2-source data/Figure 2-source data.tif]

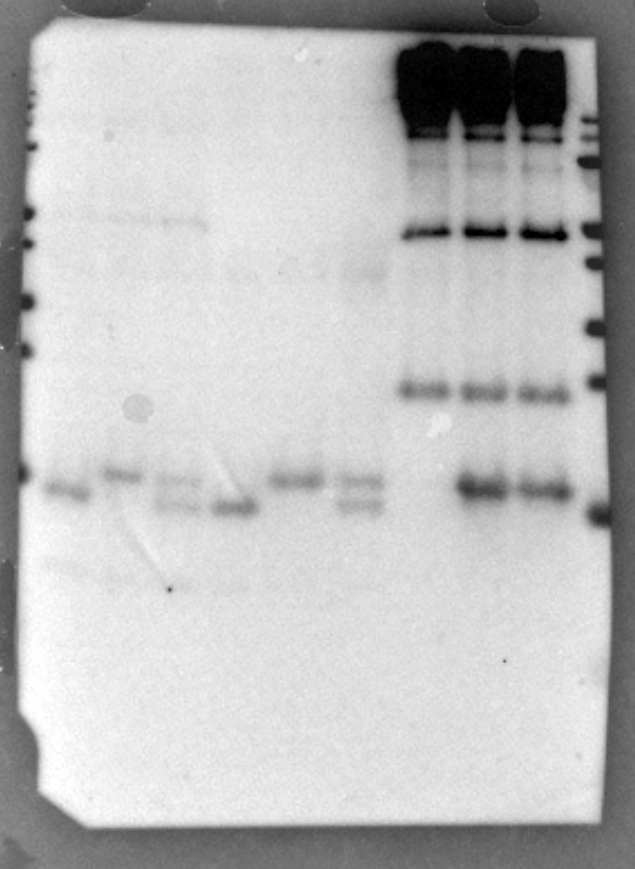

Supplement: Figure 2—source data 1. [file elife-85837-fig2-data1.zip › Figure 2-source data/Figure 2F.tif]

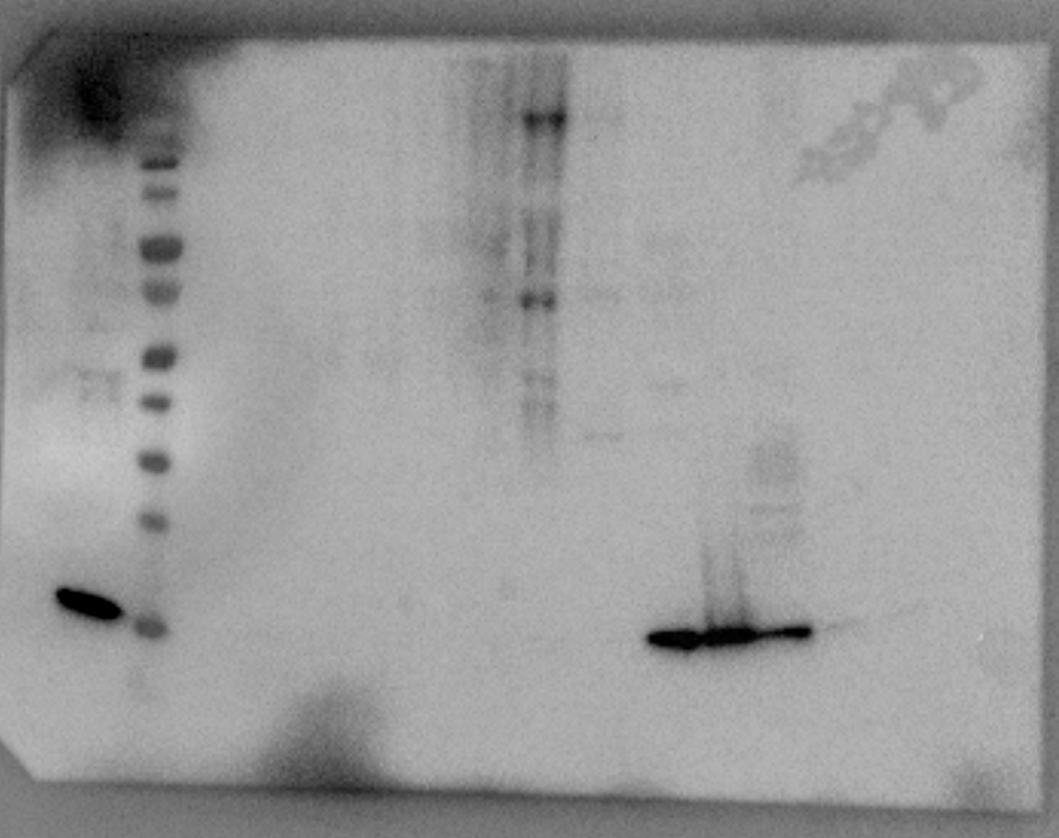

Supplement: Figure 2—source data 1. [file elife-85837-fig2-data1.zip › Figure 2-source data/Figure 2C.tif]

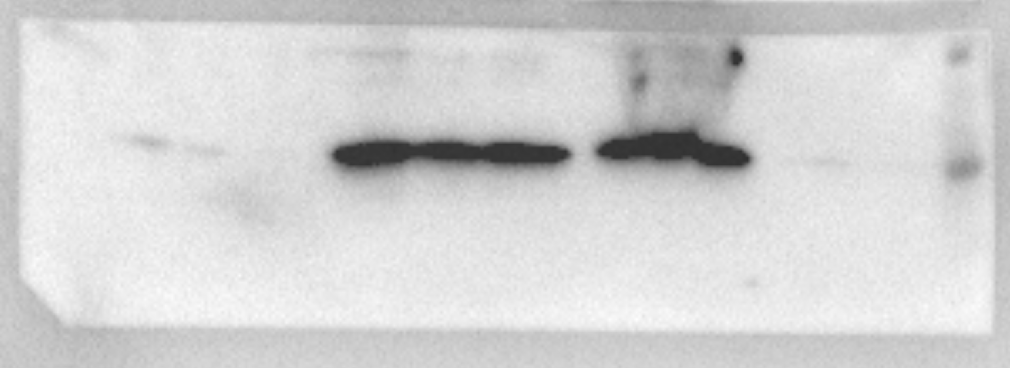

Supplement: Figure 2—source data 1. [file elife-85837-fig2-data1.zip › Figure 2-source data/Figure 2A-1.tif]

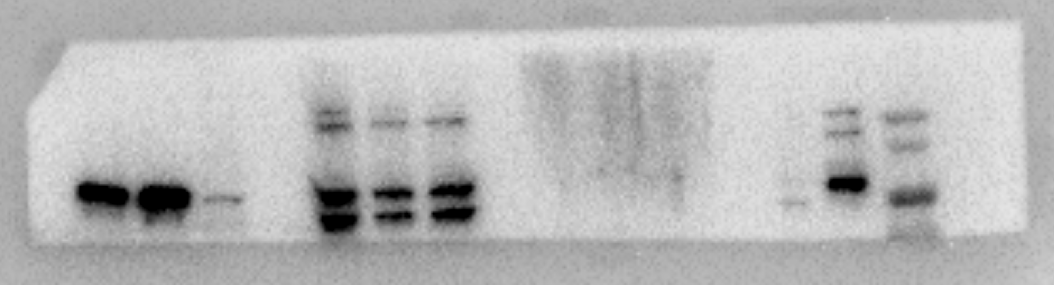

Supplement: Figure 2—source data 1. [file elife-85837-fig2-data1.zip › Figure 2-source data/Figure 2A-3.tif]

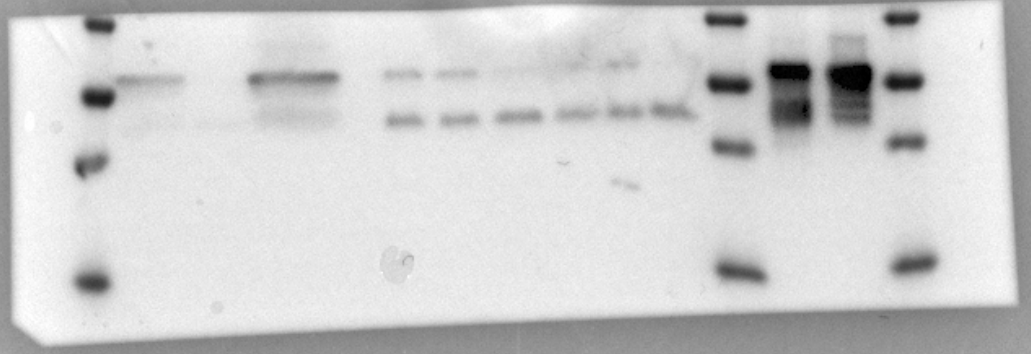

Supplement: Figure 2—source data 1. [file elife-85837-fig2-data1.zip › Figure 2-source data/Figure 2A-2.tif]

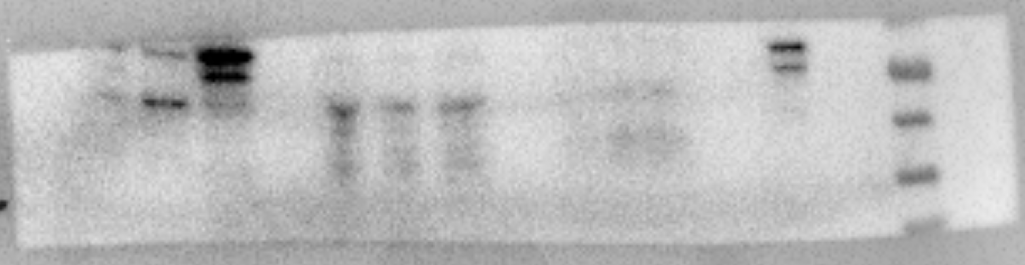

Supplement: Figure 2—source data 1. [file elife-85837-fig2-data1.zip › Figure 2-source data/Figure 2A-5.tif]

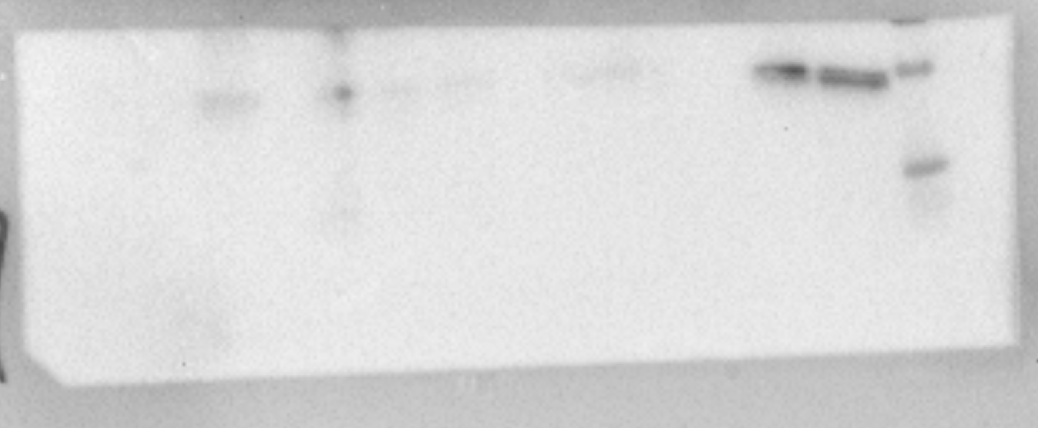

Supplement: Figure 2—source data 1. [file elife-85837-fig2-data1.zip › Figure 2-source data/Figure 2A-4.tif]

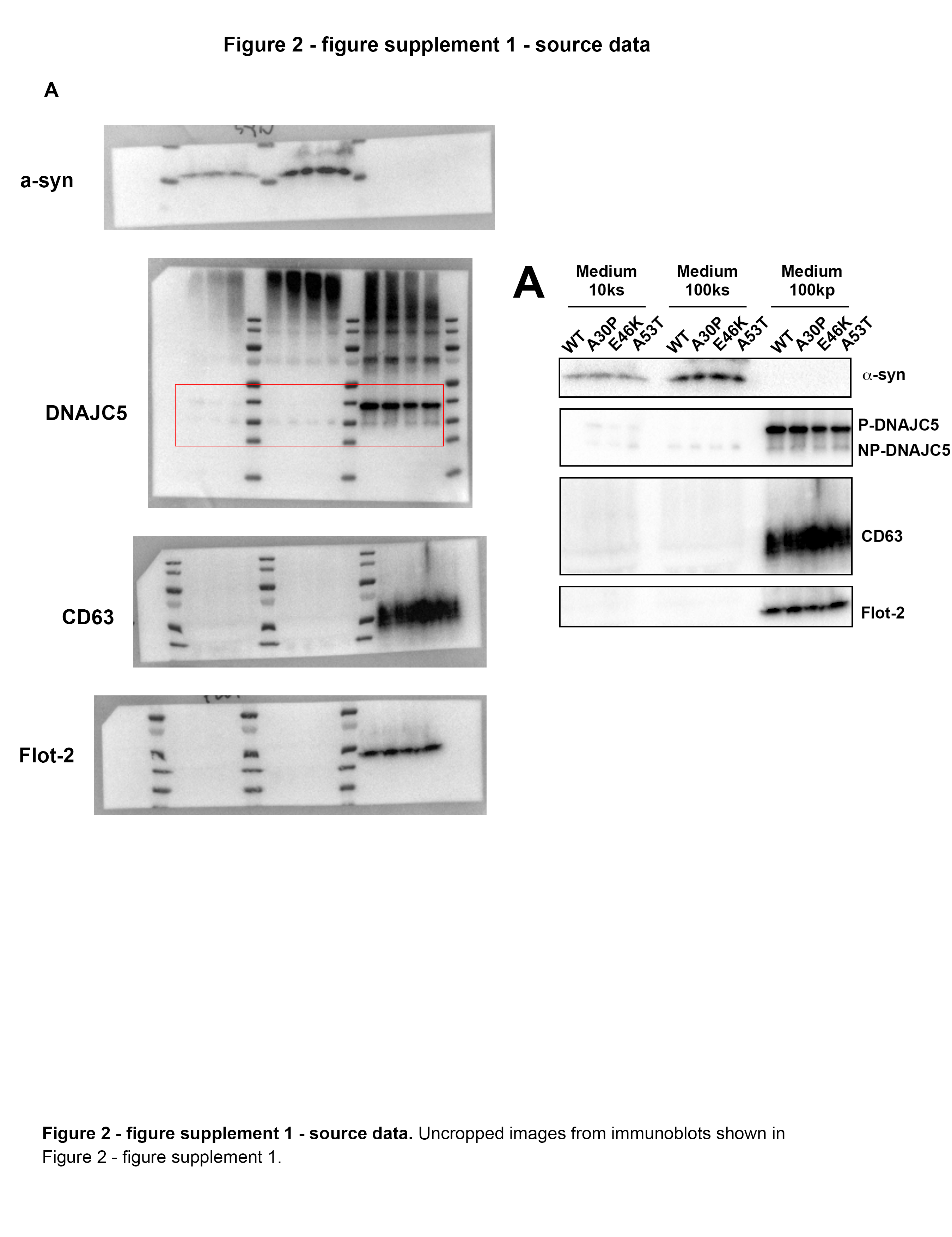

Supplement: Figure 2—figure supplement 1—source data 1. [file elife-85837-fig2-figsupp1-data1.zip › Figure 2-figure supplement 1-source data/Figure 2-figure supplement 1-source data.tif]

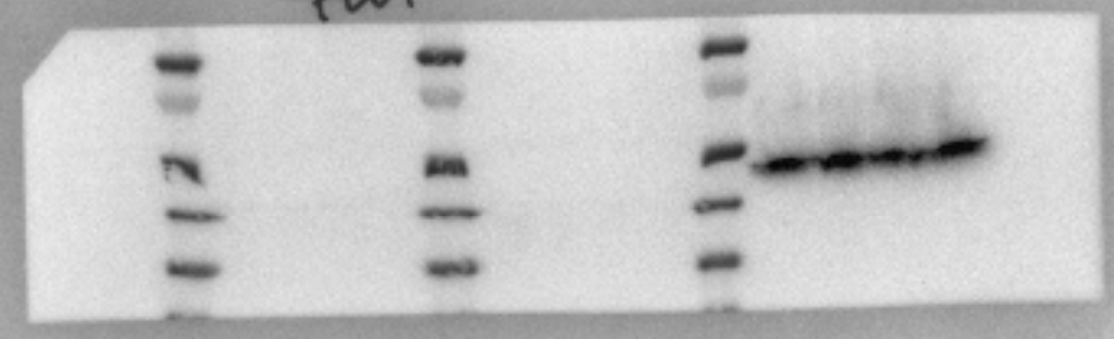

Supplement: Figure 2—figure supplement 1—source data 1. [file elife-85837-fig2-figsupp1-data1.zip › Figure 2-figure supplement 1-source data/Figure 2- figure supplement 1A-4.tif]

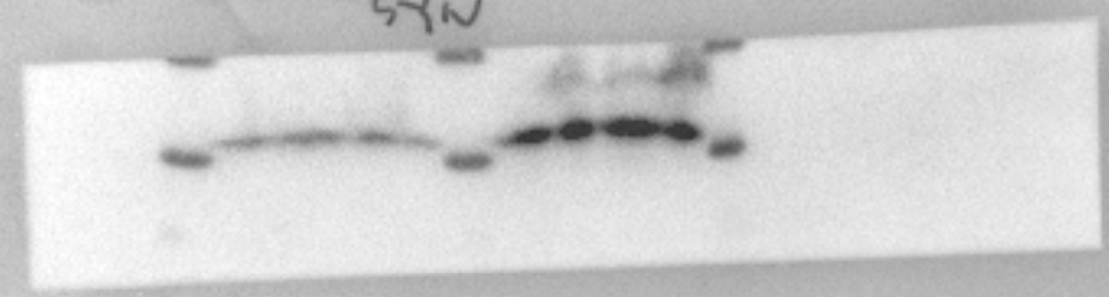

Supplement: Figure 2—figure supplement 1—source data 1. [file elife-85837-fig2-figsupp1-data1.zip › Figure 2-figure supplement 1-source data/Figure 2- figure supplement 1A-1.tif]

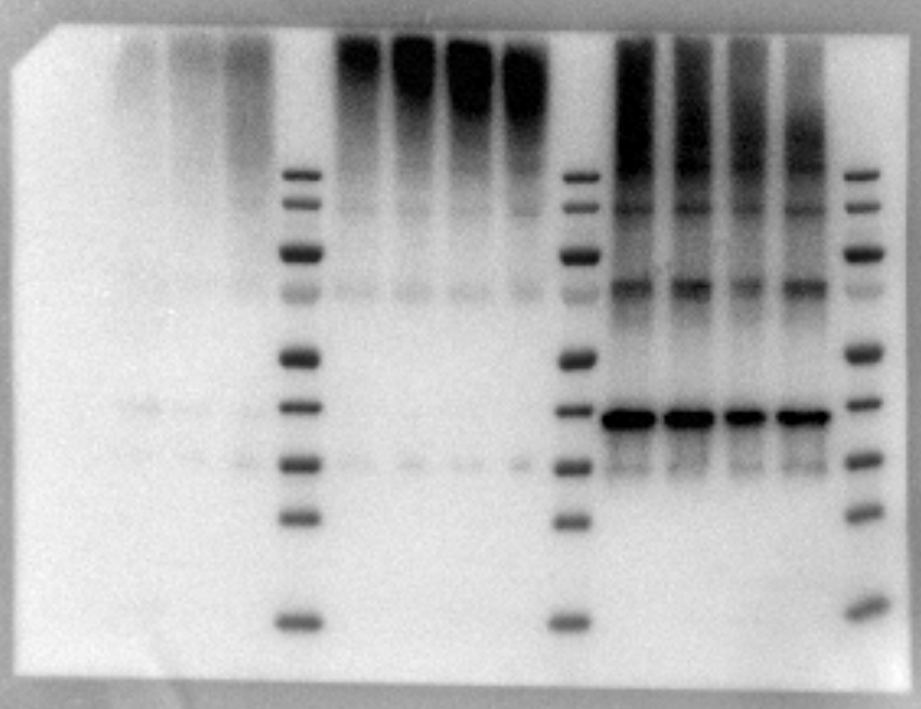

Supplement: Figure 2—figure supplement 1—source data 1. [file elife-85837-fig2-figsupp1-data1.zip › Figure 2-figure supplement 1-source data/Figure 2- figure supplement 1A-2.tif]

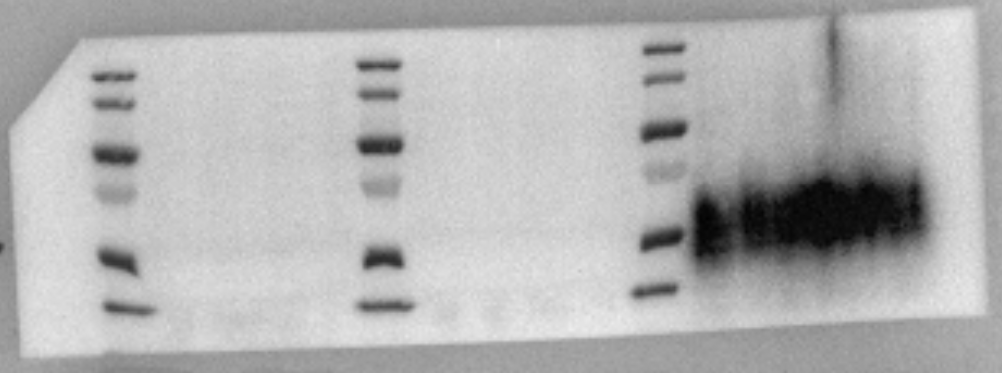

Supplement: Figure 2—figure supplement 1—source data 1. [file elife-85837-fig2-figsupp1-data1.zip › Figure 2-figure supplement 1-source data/Figure 2- figure supplement 1A-3.tif]

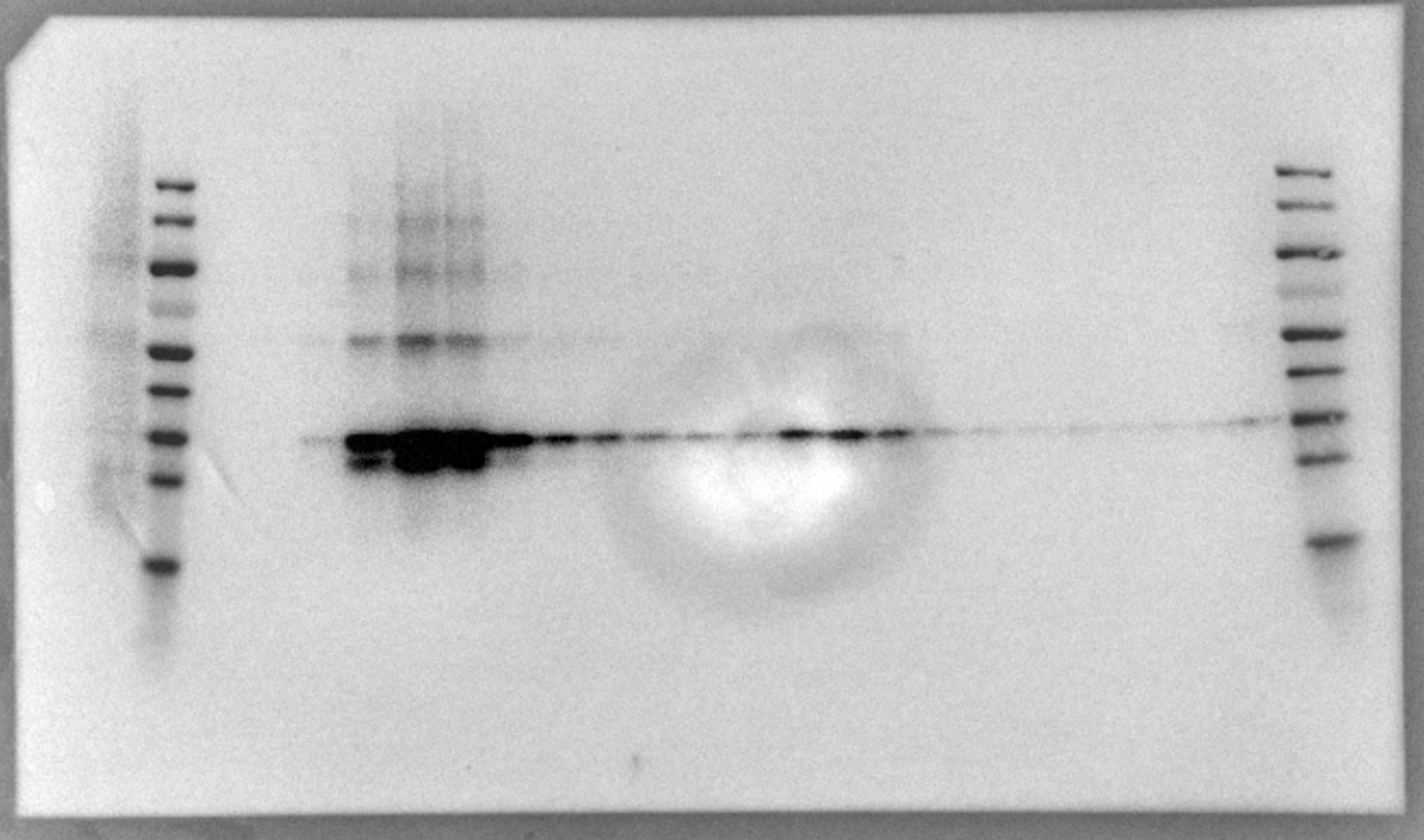

Supplement: Figure 2—figure supplement 2—source data 1. [file elife-85837-fig2-figsupp2-data1.zip › Figure 2-figure supplement 2-source data/Figure 2- figure supplement 2B-1.tif]

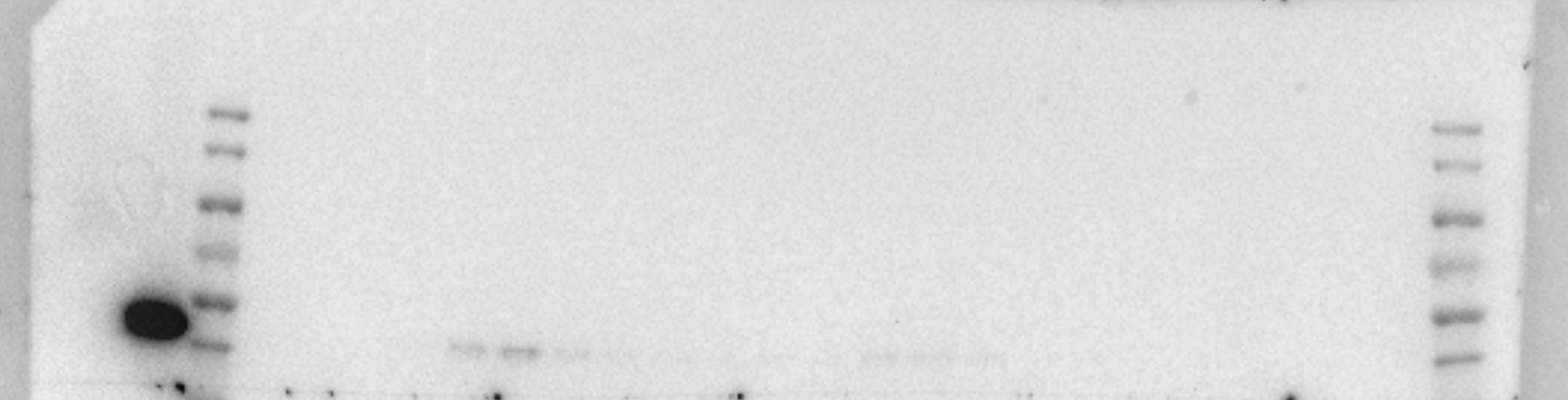

Supplement: Figure 2—figure supplement 2—source data 1. [file elife-85837-fig2-figsupp2-data1.zip › Figure 2-figure supplement 2-source data/Figure 2- figure supplement 2B-2.tif]

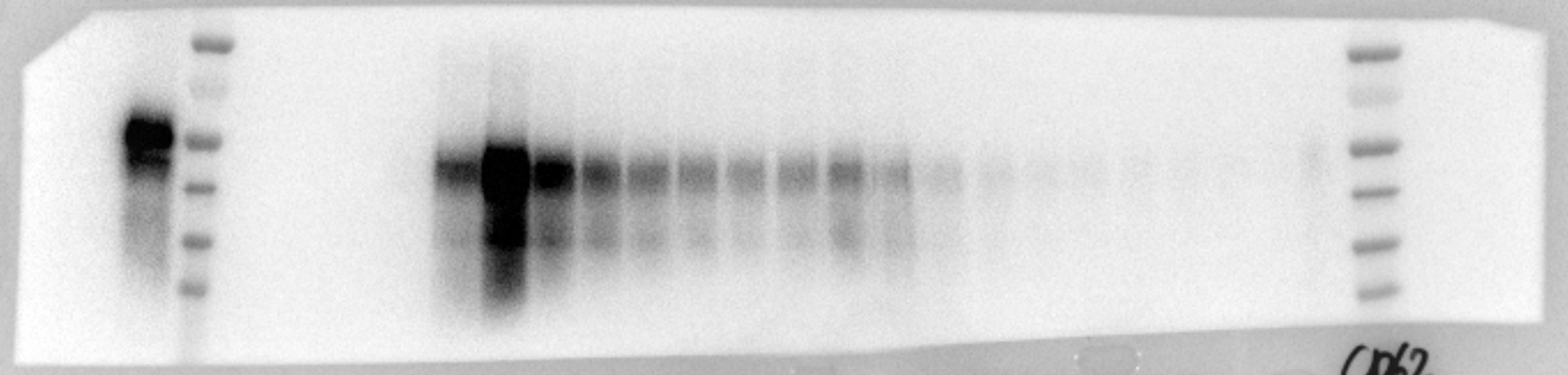

Supplement: Figure 2—figure supplement 2—source data 1. [file elife-85837-fig2-figsupp2-data1.zip › Figure 2-figure supplement 2-source data/Figure 2- figure supplement 2B-3.tif]

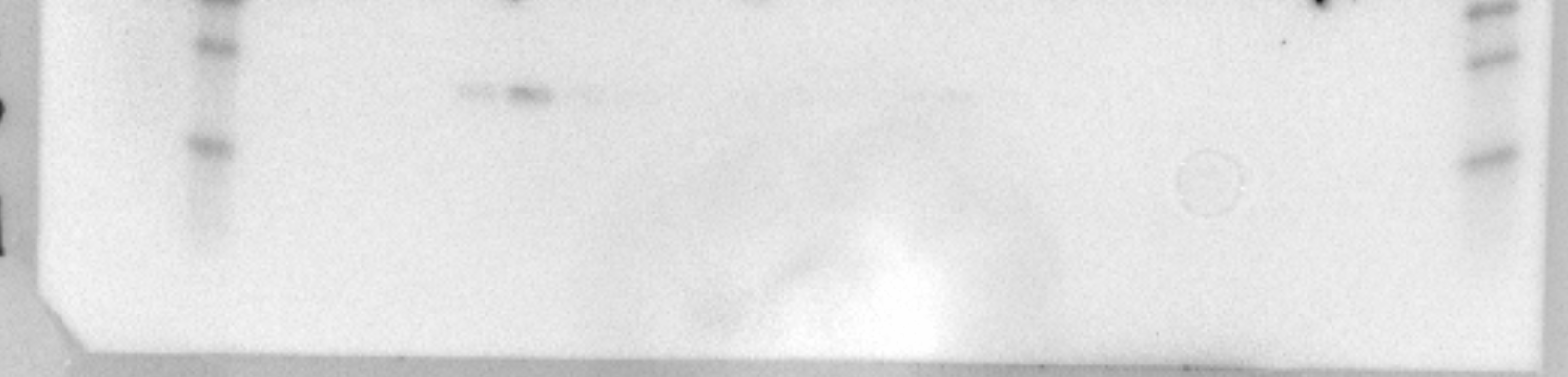

Supplement: Figure 2—figure supplement 2—source data 1. [file elife-85837-fig2-figsupp2-data1.zip › Figure 2-figure supplement 2-source data/Figure 2- figure supplement 2B-4.tif]

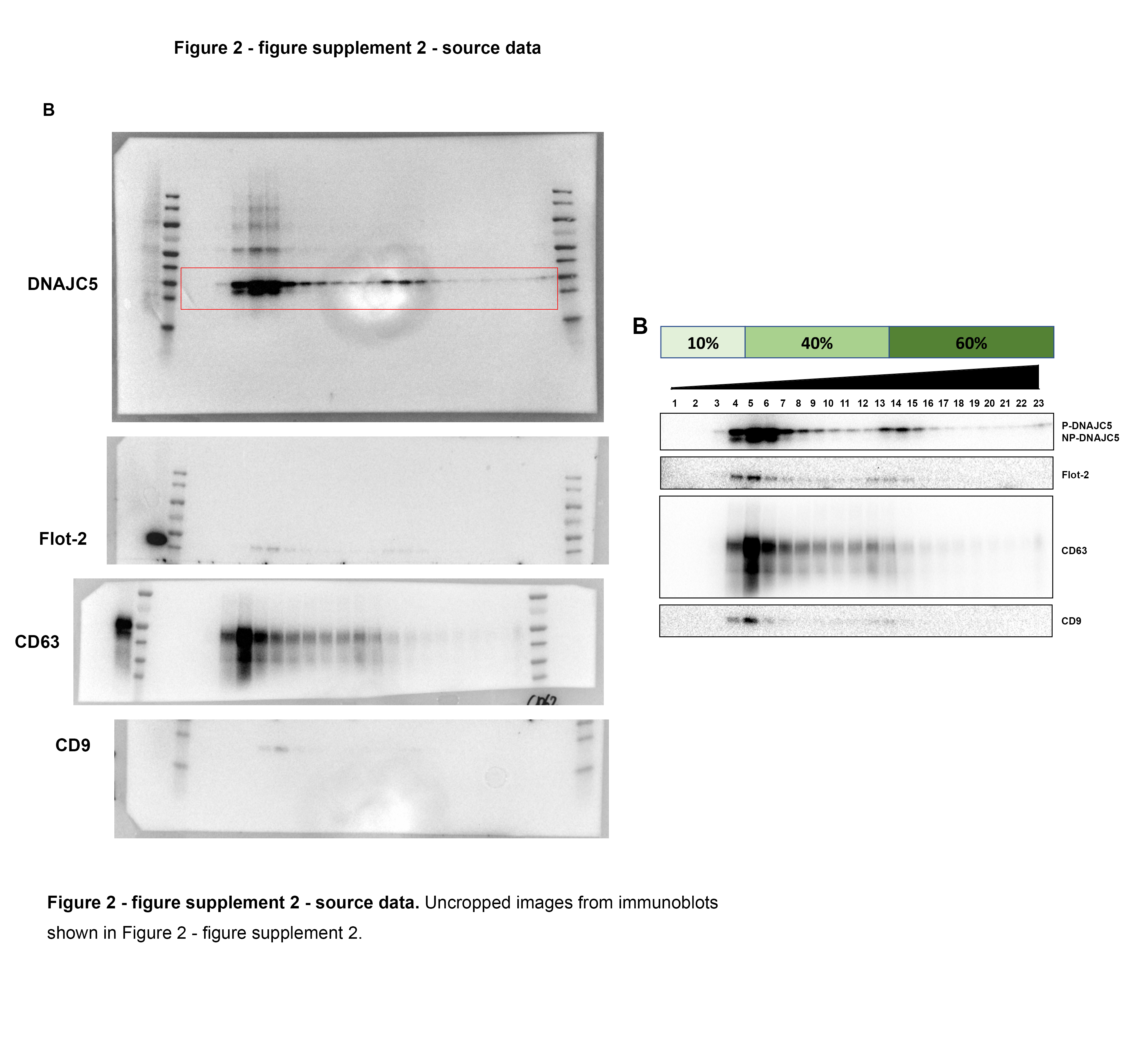

Supplement: Figure 2—figure supplement 2—source data 1. [file elife-85837-fig2-figsupp2-data1.zip › Figure 2-figure supplement 2-source data/Figure 2-figure supplement 2-source data.tif]

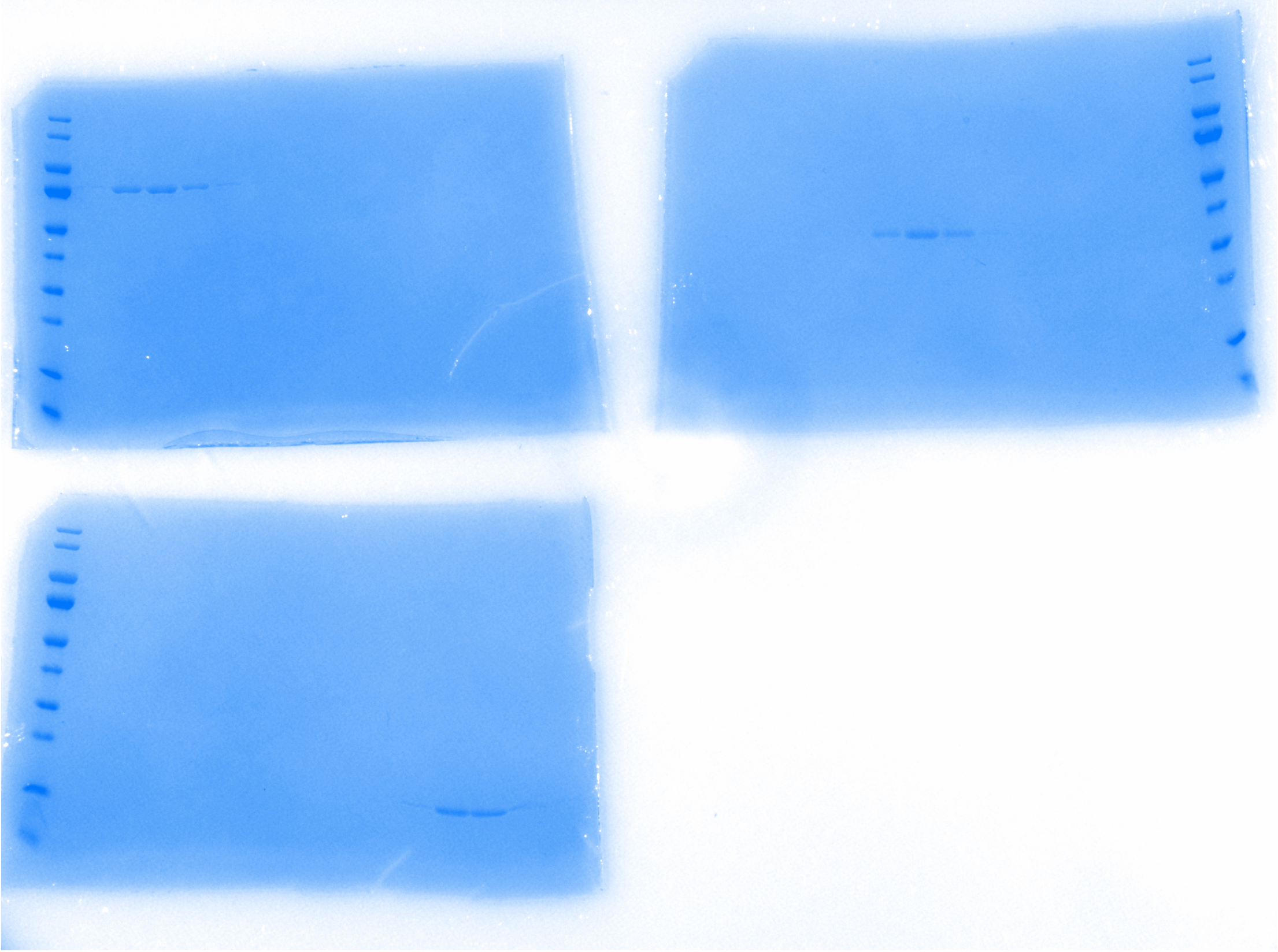

Supplement: Figure 2—figure supplement 3—source data 1. [file elife-85837-fig2-figsupp3-data1.zip › Figure 2-figure supplement 3-source data/Figure 2-figure supplement 3BDF.tif]

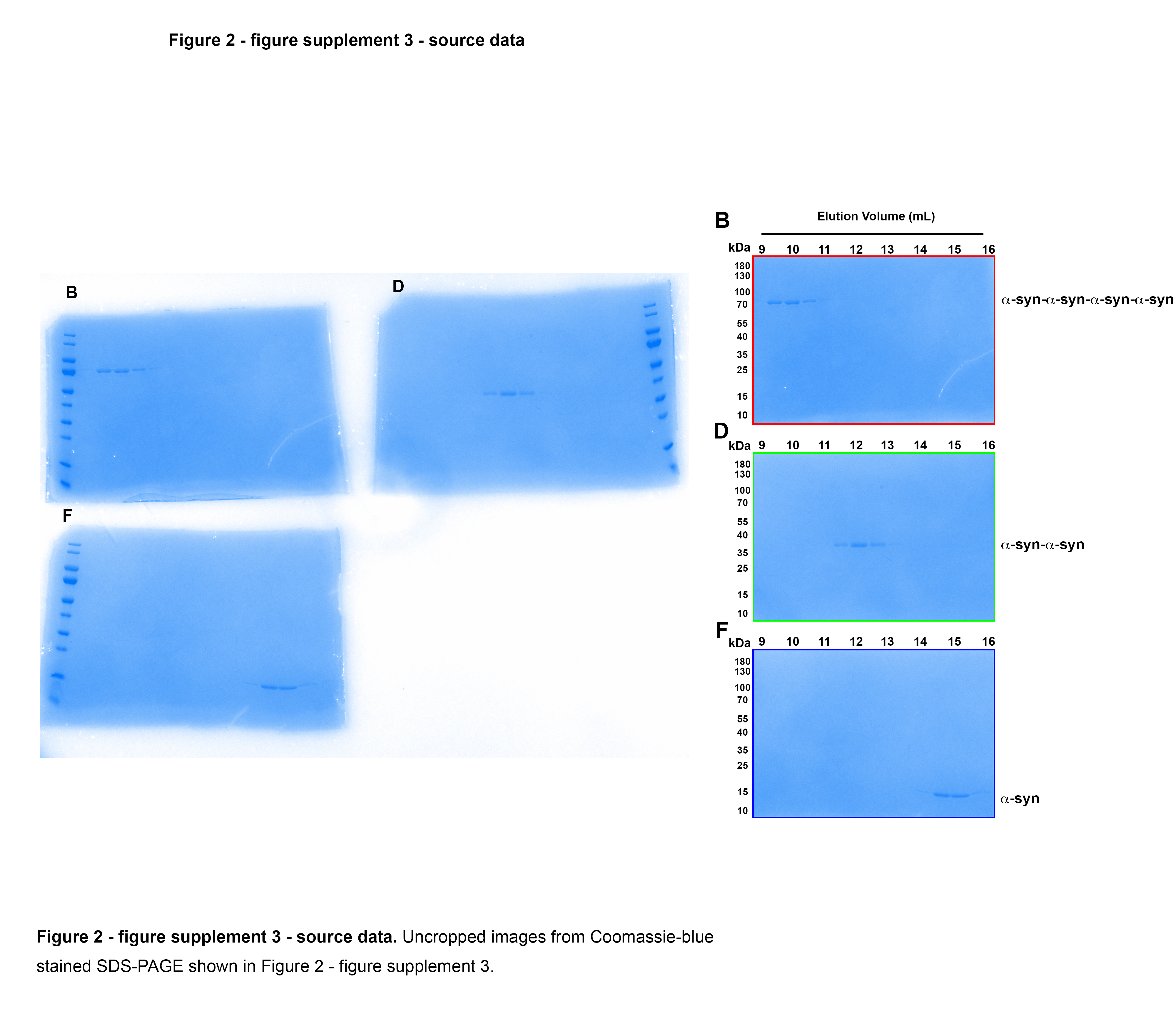

Supplement: Figure 2—figure supplement 3—source data 1. [file elife-85837-fig2-figsupp3-data1.zip › Figure 2-figure supplement 3-source data/Figure 2-figure supplement 3-source data.tif]

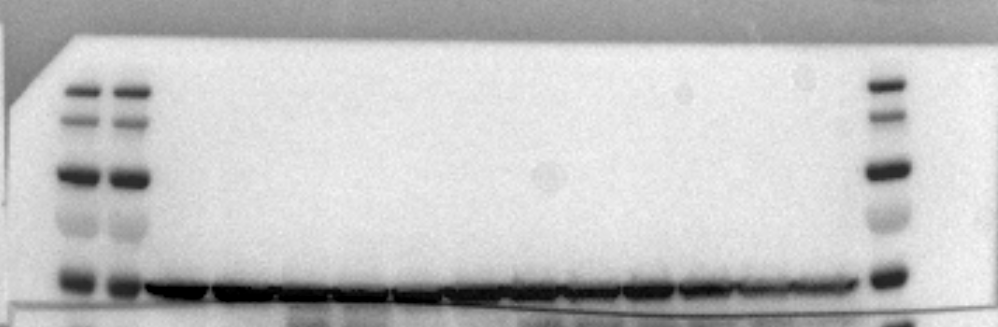

Supplement: Figure 3—source data 1. [file elife-85837-fig3-data1.zip › Figure 3-source data/Figure 3D-3.tif]

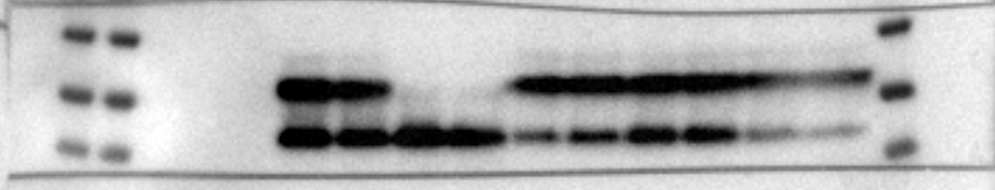

Supplement: Figure 3—source data 1. [file elife-85837-fig3-data1.zip › Figure 3-source data/Figure 3D-2.tif]

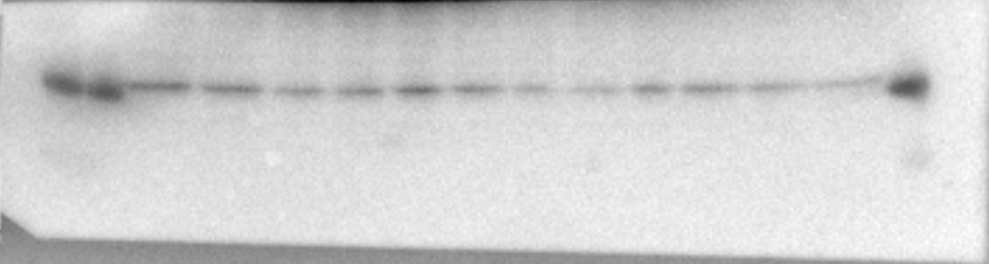

Supplement: Figure 3—source data 1. [file elife-85837-fig3-data1.zip › Figure 3-source data/Figure 3D-1.tif]

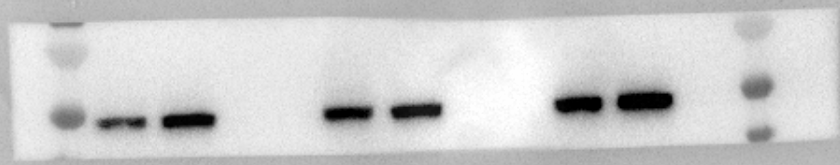

Supplement: Figure 3—source data 1. [file elife-85837-fig3-data1.zip › Figure 3-source data/Figure 3A-2.tif]

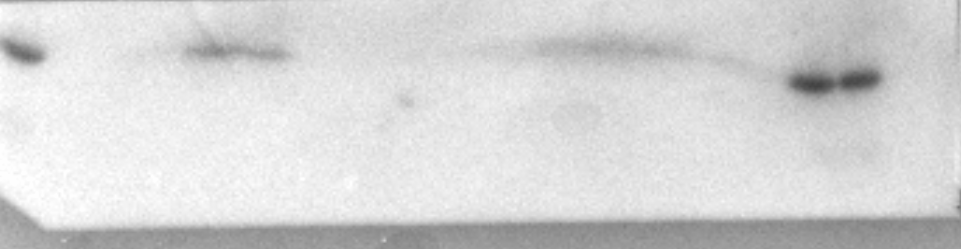

Supplement: Figure 3—source data 1. [file elife-85837-fig3-data1.zip › Figure 3-source data/Figure 3C-1.tif]

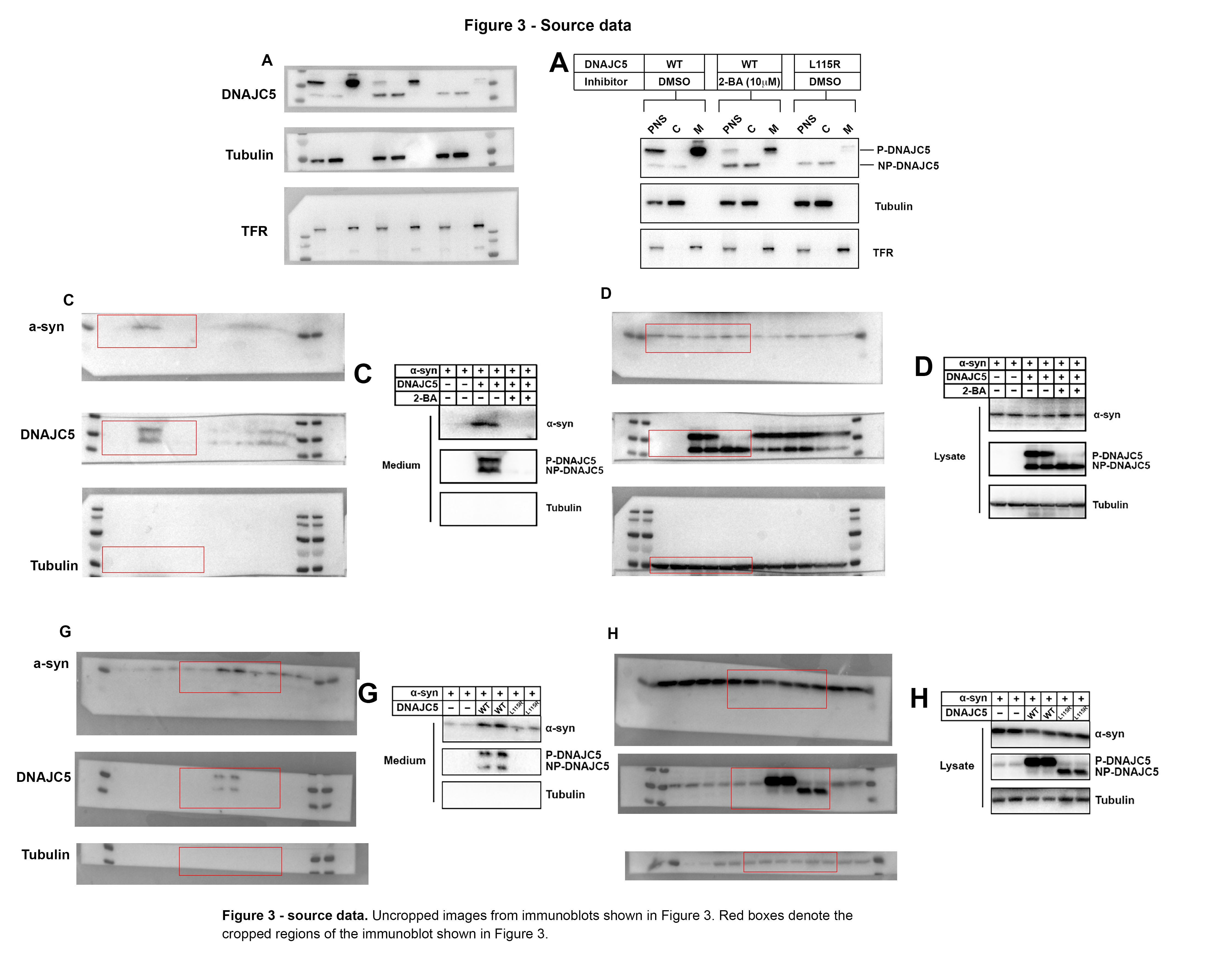

Supplement: Figure 3—source data 1. [file elife-85837-fig3-data1.zip › Figure 3-source data/Figure 3-source data.tif]

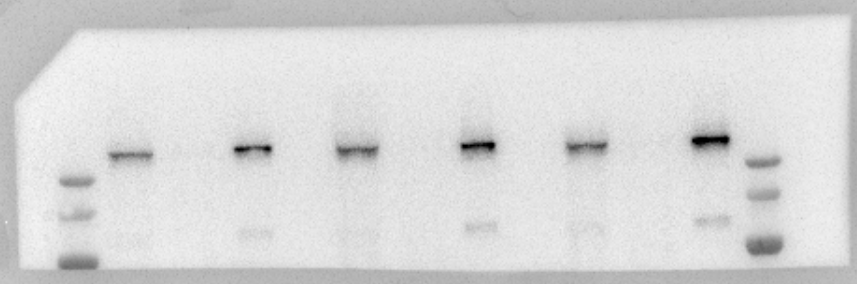

Supplement: Figure 3—source data 1. [file elife-85837-fig3-data1.zip › Figure 3-source data/Figure 3A-3.tif]

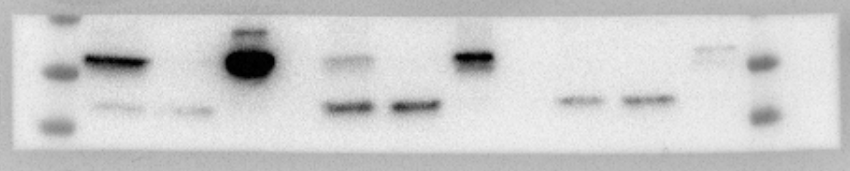

Supplement: Figure 3—source data 1. [file elife-85837-fig3-data1.zip › Figure 3-source data/Figure 3A-1.tif]

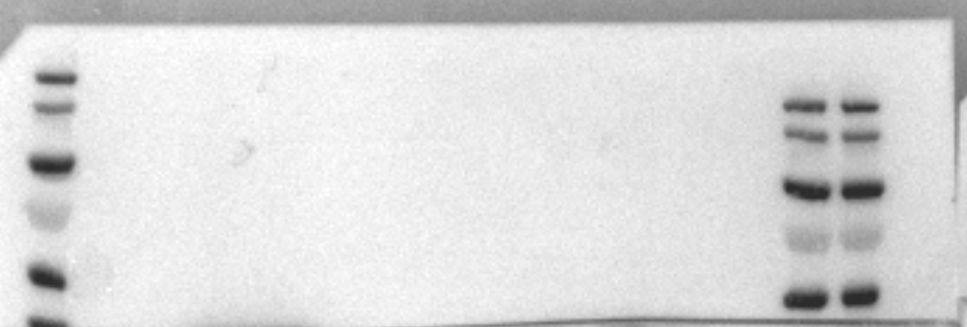

Supplement: Figure 3—source data 1. [file elife-85837-fig3-data1.zip › Figure 3-source data/Figure 3C-3.tif]

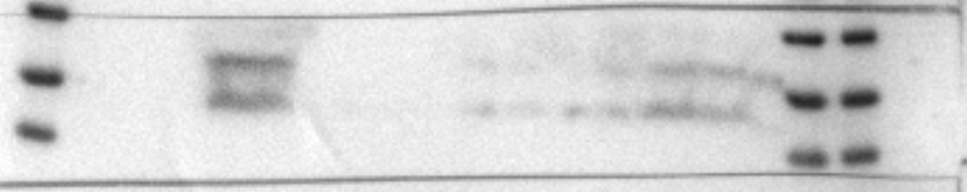

Supplement: Figure 3—source data 1. [file elife-85837-fig3-data1.zip › Figure 3-source data/Figure 3C-2.tif]

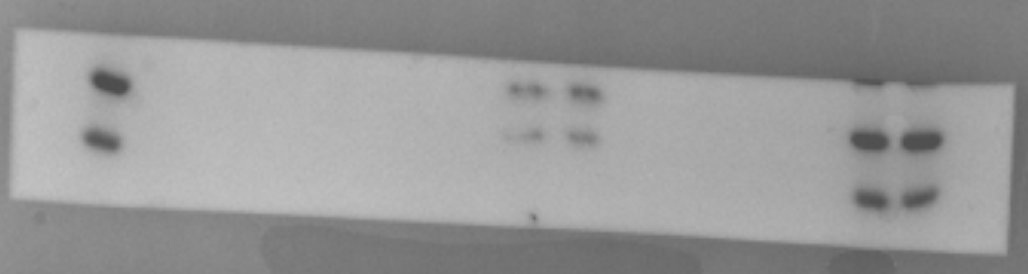

Supplement: Figure 3—source data 1. [file elife-85837-fig3-data1.zip › Figure 3-source data/Figure 3G-2.tif]

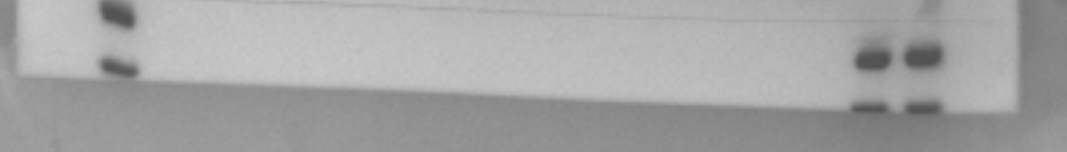

Supplement: Figure 3—source data 1. [file elife-85837-fig3-data1.zip › Figure 3-source data/Figure 3G-3.tif]

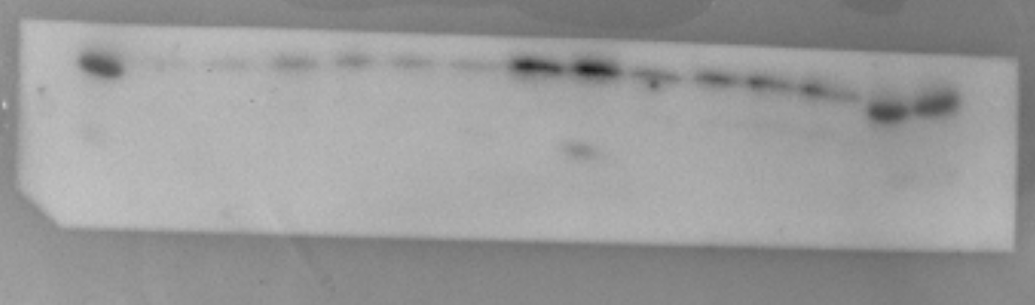

Supplement: Figure 3—source data 1. [file elife-85837-fig3-data1.zip › Figure 3-source data/Figure 3G-1.tif]

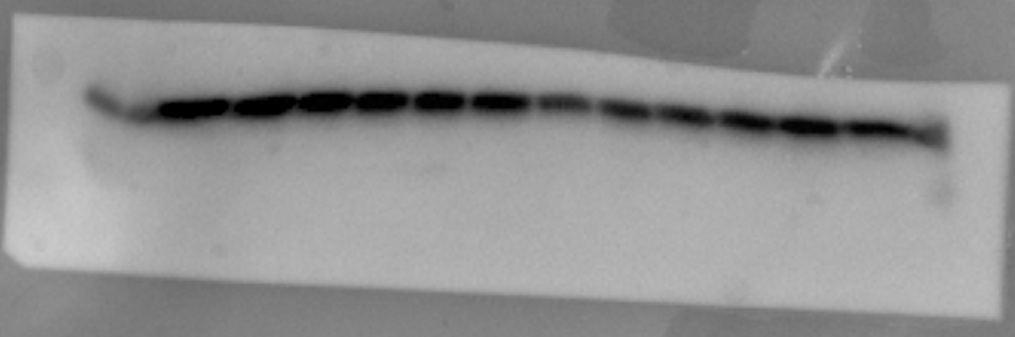

Supplement: Figure 3—source data 1. [file elife-85837-fig3-data1.zip › Figure 3-source data/Figure 3H-1.tif]

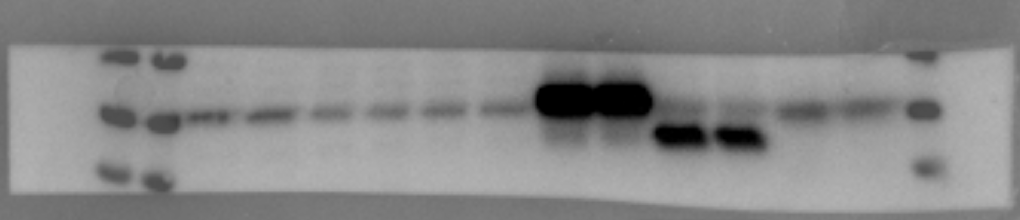

Supplement: Figure 3—source data 1. [file elife-85837-fig3-data1.zip › Figure 3-source data/Figure 3H-2.tif]

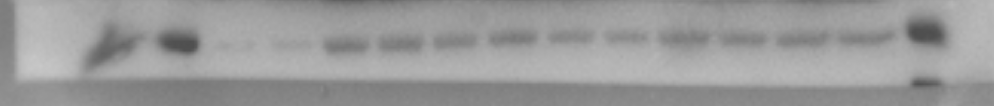

Supplement: Figure 3—source data 1. [file elife-85837-fig3-data1.zip › Figure 3-source data/Figure 3H-3.tif]

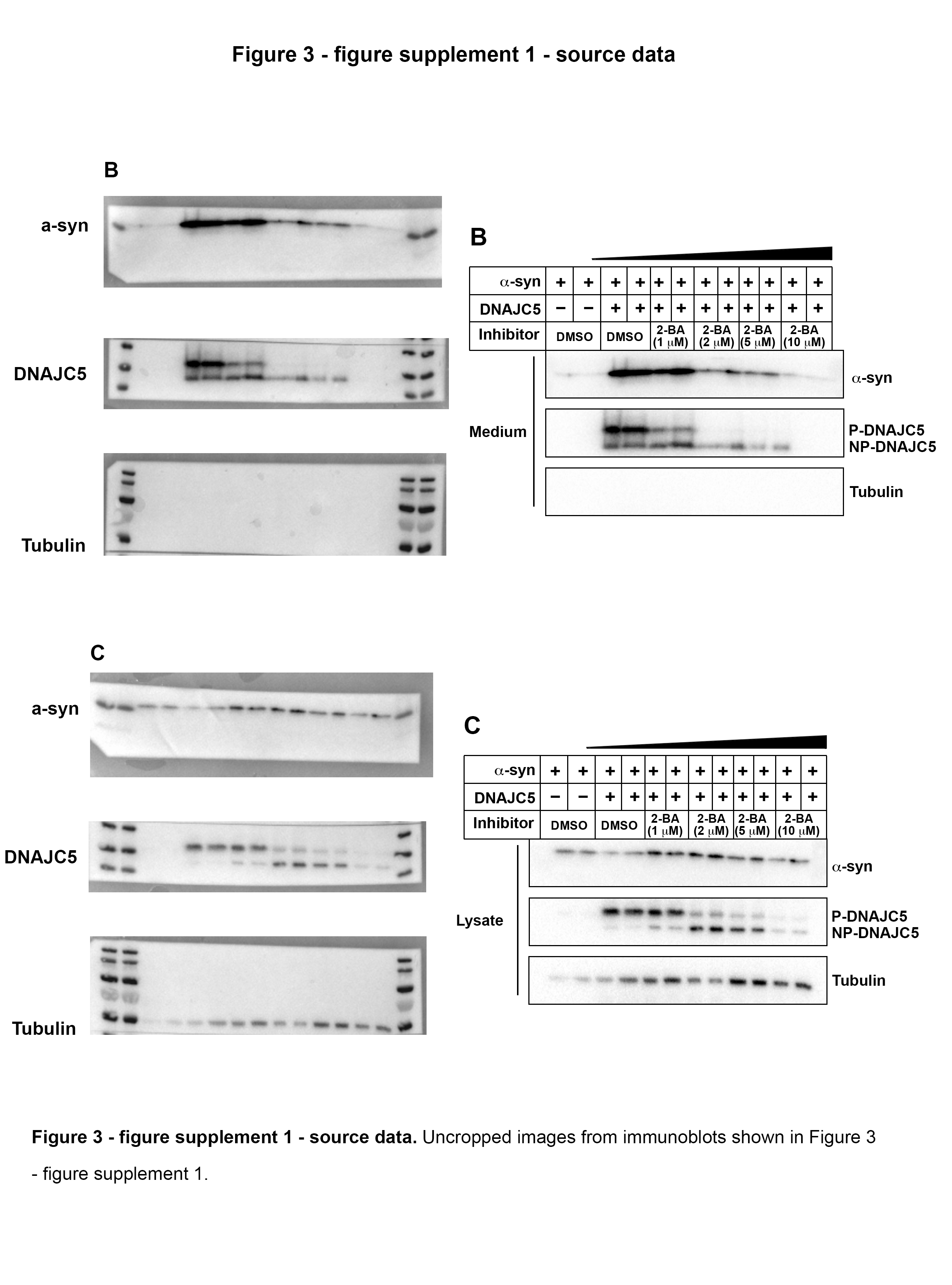

Supplement: Figure 3—figure supplement 1—source data 1. [file elife-85837-fig3-figsupp1-data1.zip › Figure 3-figure supplement 1-source data/Figure 3-figure supplement 1-source data.tif]

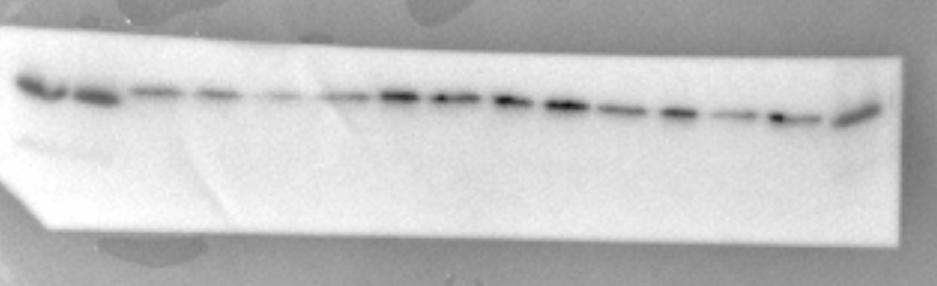

Supplement: Figure 3—figure supplement 1—source data 1. [file elife-85837-fig3-figsupp1-data1.zip › Figure 3-figure supplement 1-source data/Figure 3-figure supplement 1C-1.tif]

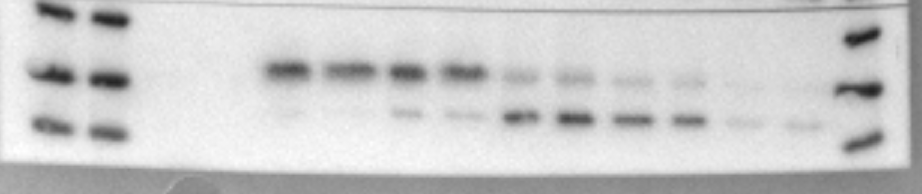

Supplement: Figure 3—figure supplement 1—source data 1. [file elife-85837-fig3-figsupp1-data1.zip › Figure 3-figure supplement 1-source data/Figure 3-figure supplement 1C-2.tif]

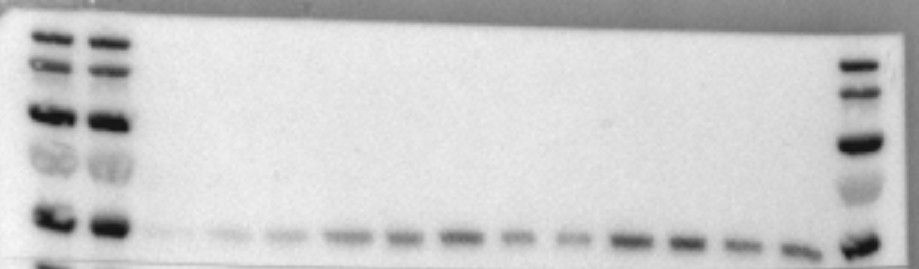

Supplement: Figure 3—figure supplement 1—source data 1. [file elife-85837-fig3-figsupp1-data1.zip › Figure 3-figure supplement 1-source data/Figure 3-figure supplement 1C-3.tif]

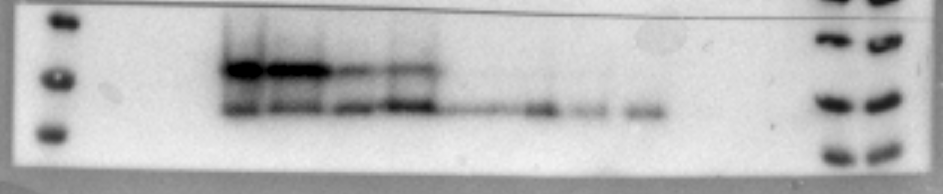

Supplement: Figure 3—figure supplement 1—source data 1. [file elife-85837-fig3-figsupp1-data1.zip › Figure 3-figure supplement 1-source data/Figure 3-figure supplement 1B-2.tif]

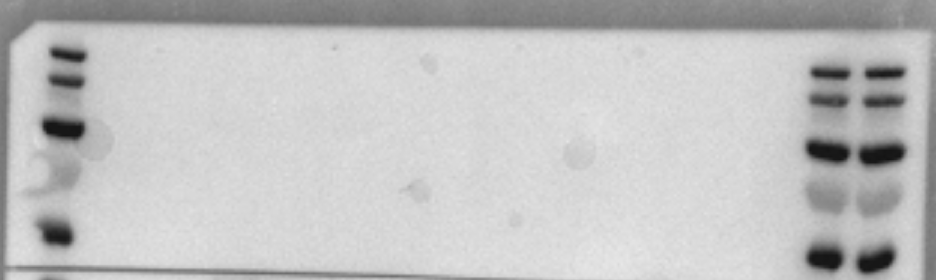

Supplement: Figure 3—figure supplement 1—source data 1. [file elife-85837-fig3-figsupp1-data1.zip › Figure 3-figure supplement 1-source data/Figure 3-figure supplement 1B-3.tif]

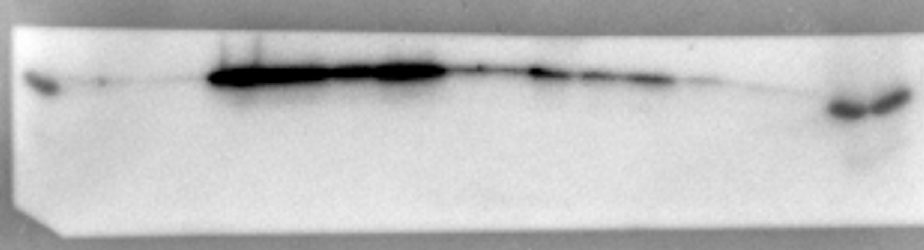

Supplement: Figure 3—figure supplement 1—source data 1. [file elife-85837-fig3-figsupp1-data1.zip › Figure 3-figure supplement 1-source data/Figure 3-figure supplement 1B-1.tif]

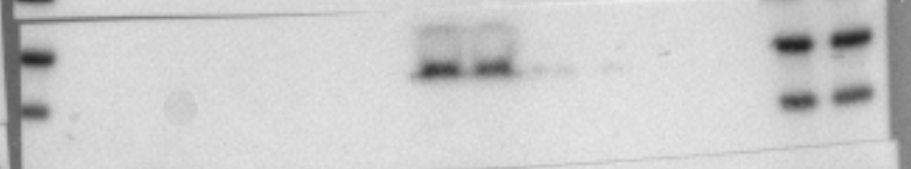

Supplement: Figure 3—figure supplement 2—source data 1. [file elife-85837-fig3-figsupp2-data1.zip › Figure 3-figure supplement 2-source data/Figure 3-figure supplement 2A-2.tif]

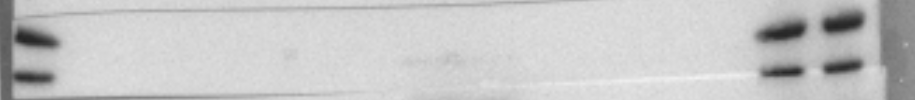

Supplement: Figure 3—figure supplement 2—source data 1. [file elife-85837-fig3-figsupp2-data1.zip › Figure 3-figure supplement 2-source data/Figure 3-figure supplement 2A-3.tif]

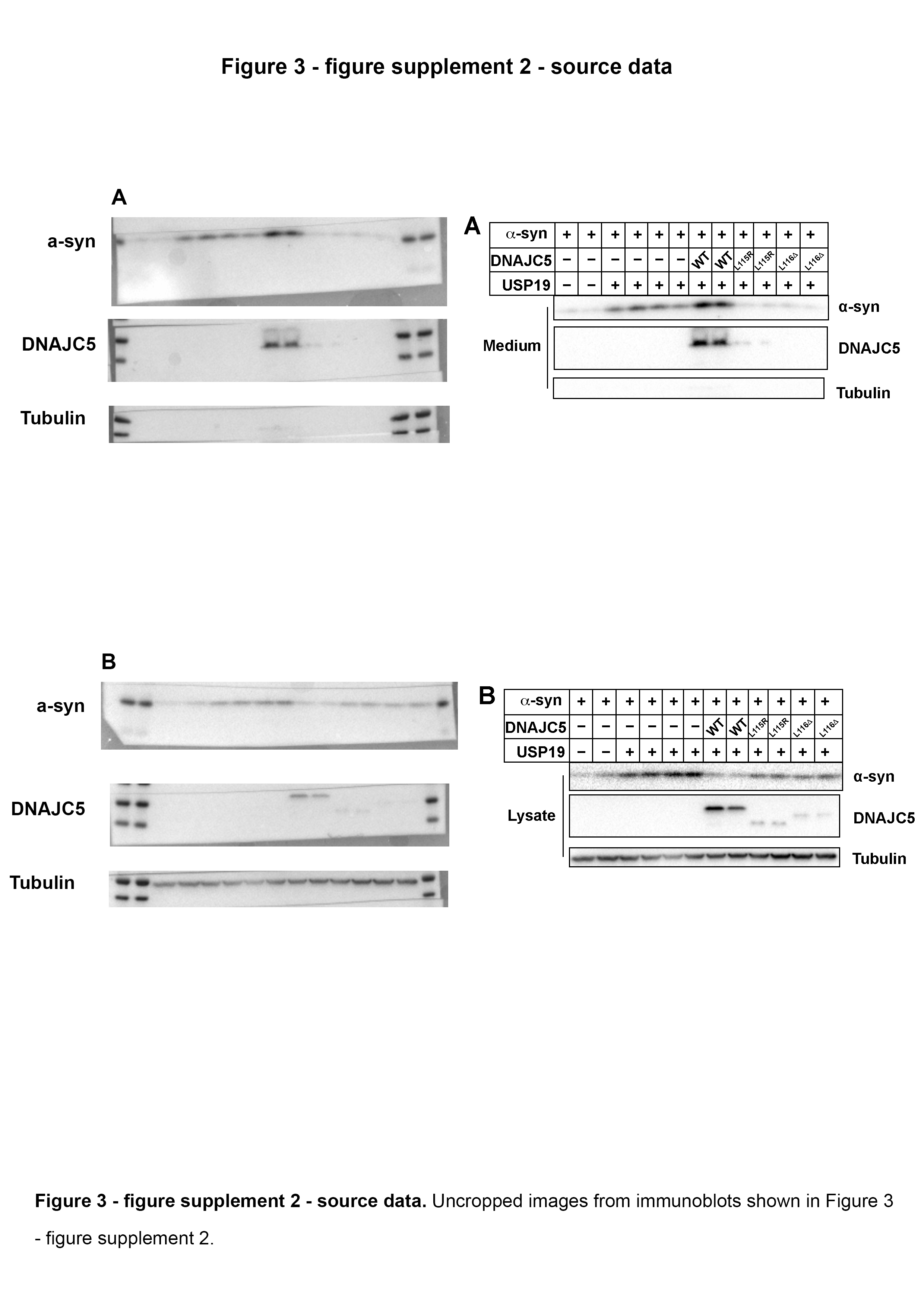

Supplement: Figure 3—figure supplement 2—source data 1. [file elife-85837-fig3-figsupp2-data1.zip › Figure 3-figure supplement 2-source data/Figure 3-figure supplement 2-source data.tif]

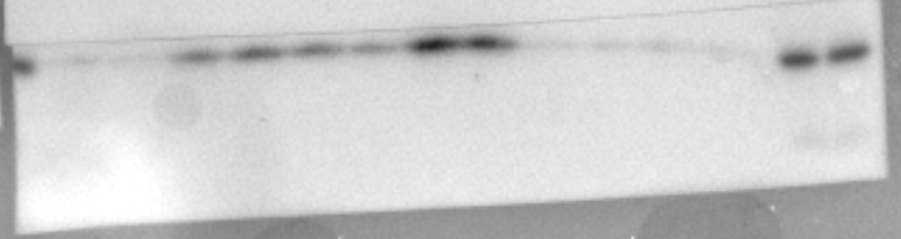

Supplement: Figure 3—figure supplement 2—source data 1. [file elife-85837-fig3-figsupp2-data1.zip › Figure 3-figure supplement 2-source data/Figure 3-figure supplement 2A-1.tif]

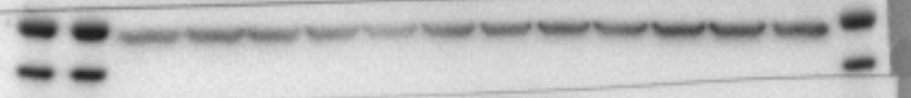

Supplement: Figure 3—figure supplement 2—source data 1. [file elife-85837-fig3-figsupp2-data1.zip › Figure 3-figure supplement 2-source data/Figure 3-figure supplement 2B-3.tif]

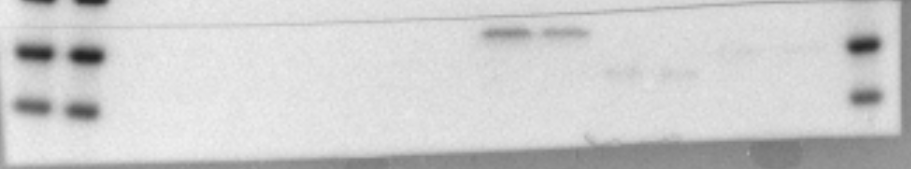

Supplement: Figure 3—figure supplement 2—source data 1. [file elife-85837-fig3-figsupp2-data1.zip › Figure 3-figure supplement 2-source data/Figure 3-figure supplement 2B-2.tif]

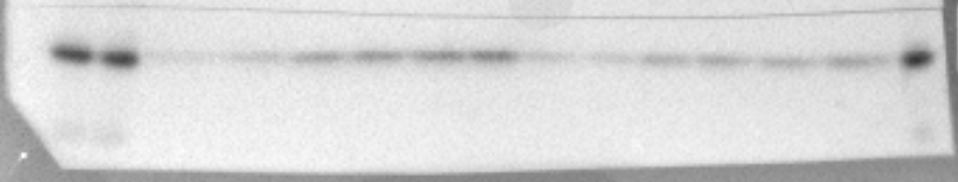

Supplement: Figure 3—figure supplement 2—source data 1. [file elife-85837-fig3-figsupp2-data1.zip › Figure 3-figure supplement 2-source data/Figure 3-figure supplement 2B-1.tif]

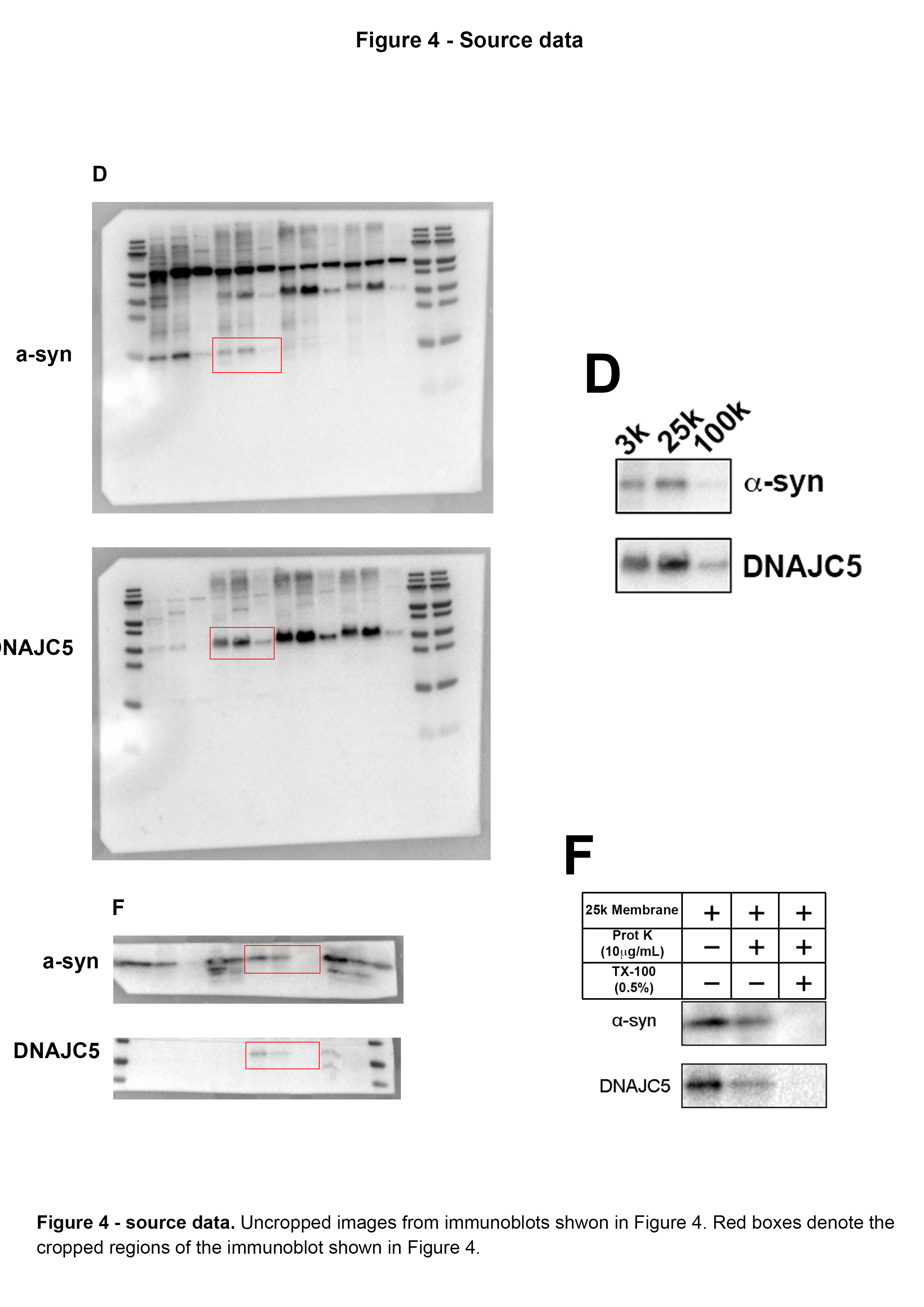

Supplement: Figure 4—source data 1. [file elife-85837-fig4-data1.zip › Figure 4-source data/Figure 4-source data.tif]

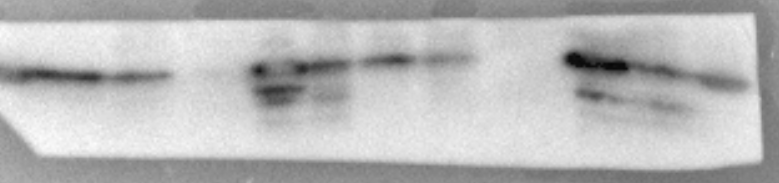

Supplement: Figure 4—source data 1. [file elife-85837-fig4-data1.zip › Figure 4-source data/Figure 4F-1.tif]

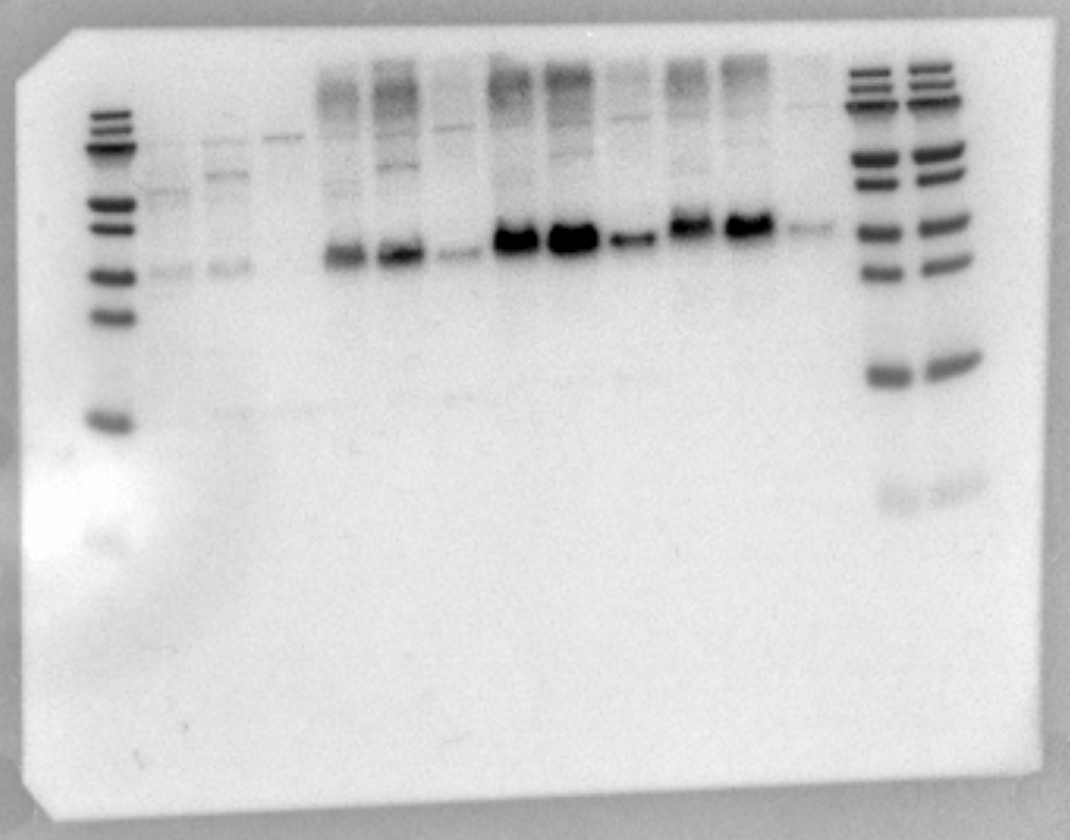

Supplement: Figure 4—source data 1. [file elife-85837-fig4-data1.zip › Figure 4-source data/Figure 4D-2.tif]

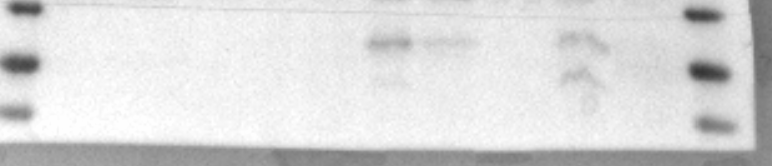

Supplement: Figure 4—source data 1. [file elife-85837-fig4-data1.zip › Figure 4-source data/Figure 4F-2.tif]

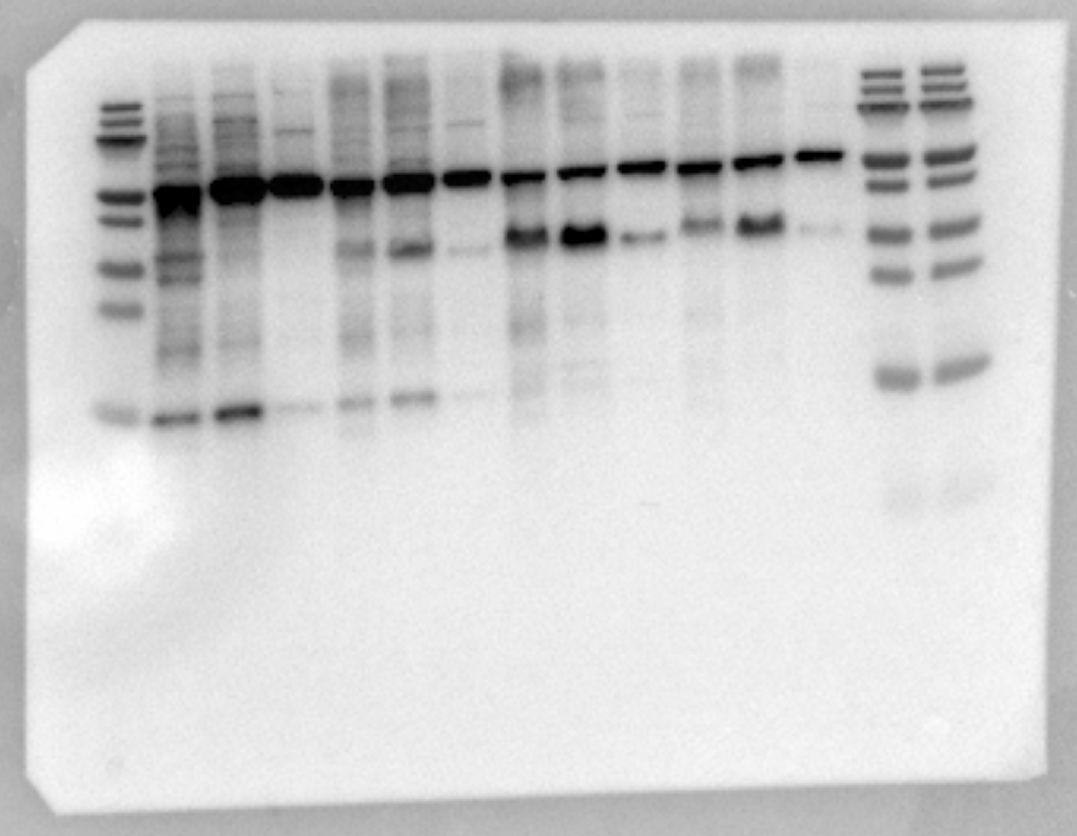

Supplement: Figure 4—source data 1. [file elife-85837-fig4-data1.zip › Figure 4-source data/Figure 4D-1.tif]

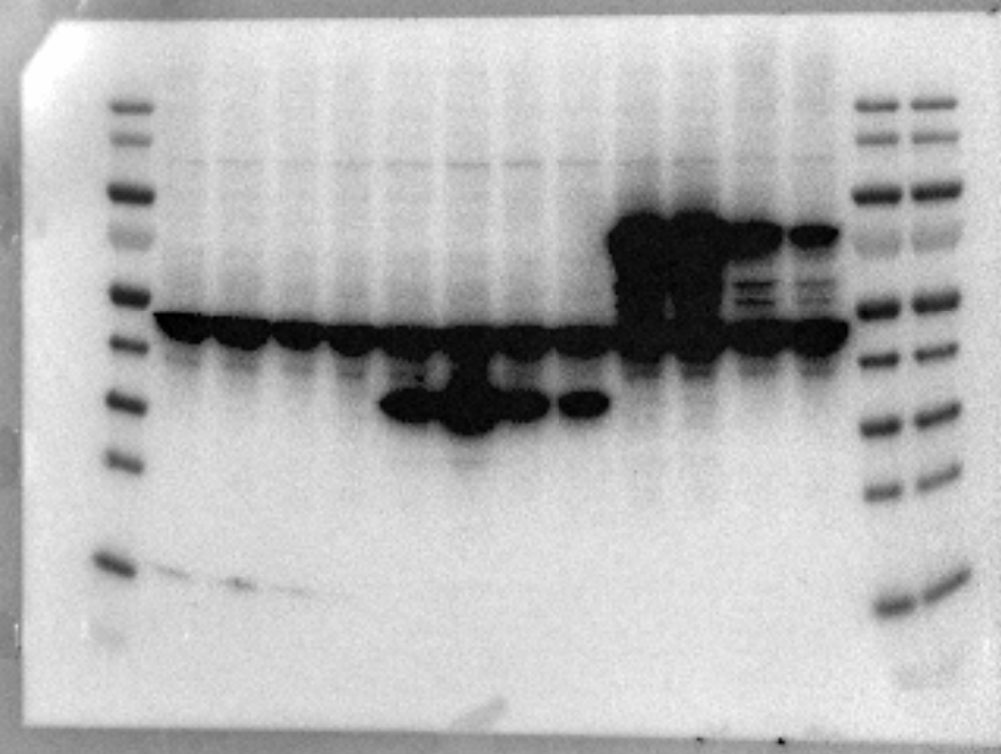

Supplement: Figure 5—source data 1. [file elife-85837-fig5-data1.zip › Figure 5-source data/Figure 5C-2.tif]

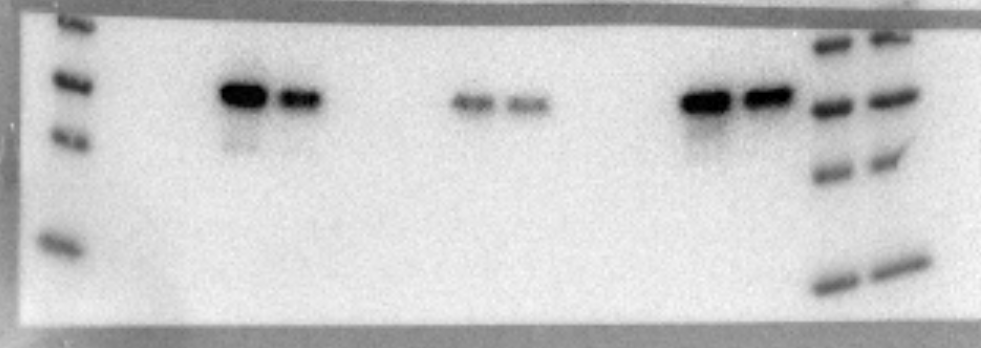

Supplement: Figure 5—source data 1. [file elife-85837-fig5-data1.zip › Figure 5-source data/Figure 5C-3.tif]

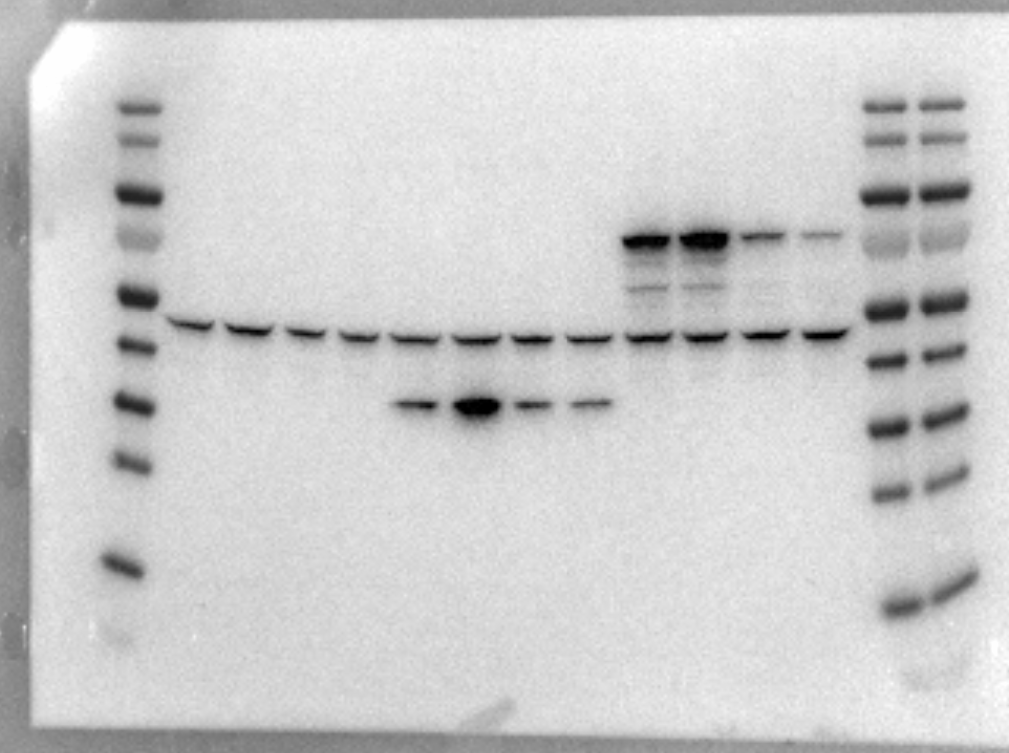

Supplement: Figure 5—source data 1. [file elife-85837-fig5-data1.zip › Figure 5-source data/Figure 5C-1.tif]

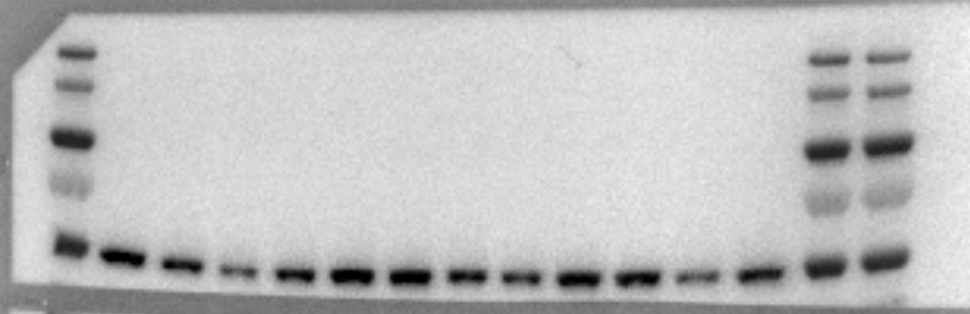

Supplement: Figure 5—source data 1. [file elife-85837-fig5-data1.zip › Figure 5-source data/Figure 5C-4.tif]

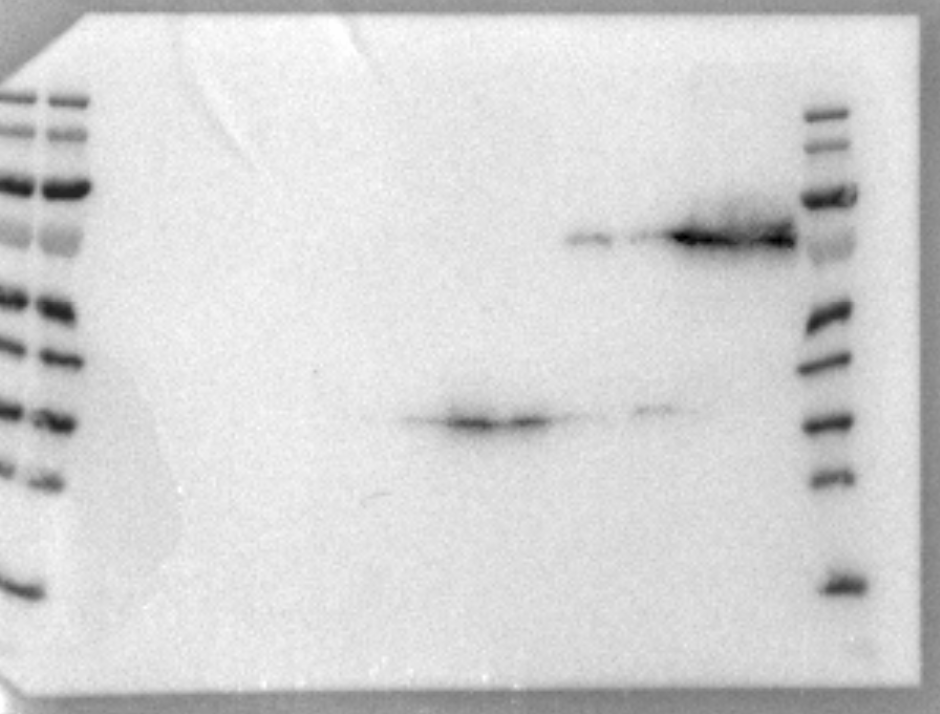

Supplement: Figure 5—source data 1. [file elife-85837-fig5-data1.zip › Figure 5-source data/Figure 5B-1.tif]

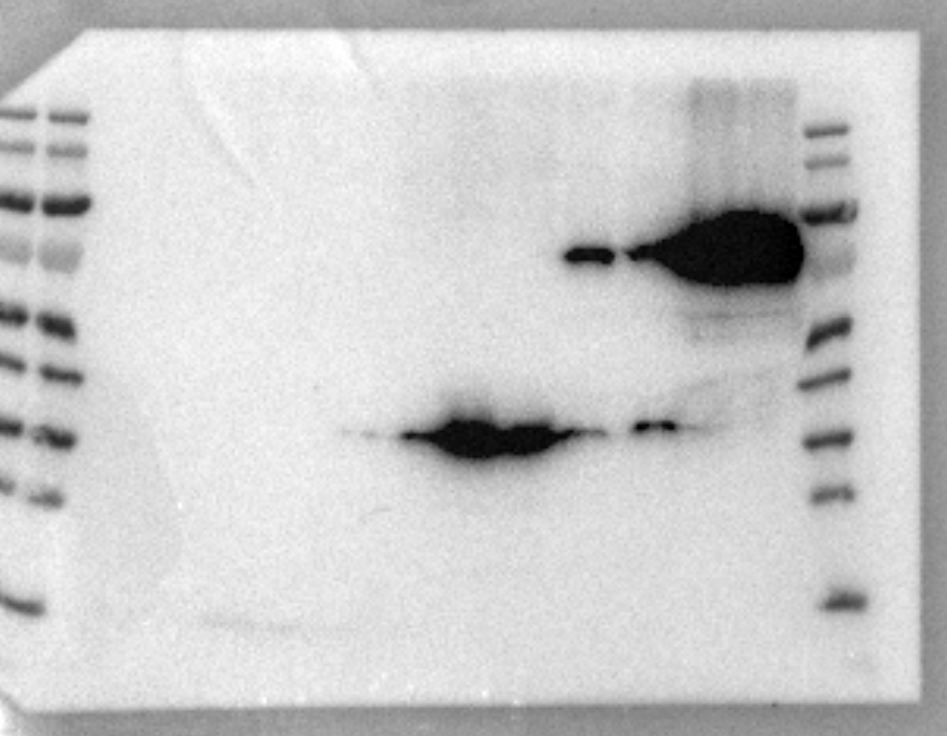

Supplement: Figure 5—source data 1. [file elife-85837-fig5-data1.zip › Figure 5-source data/Figure 5B-2.tif]

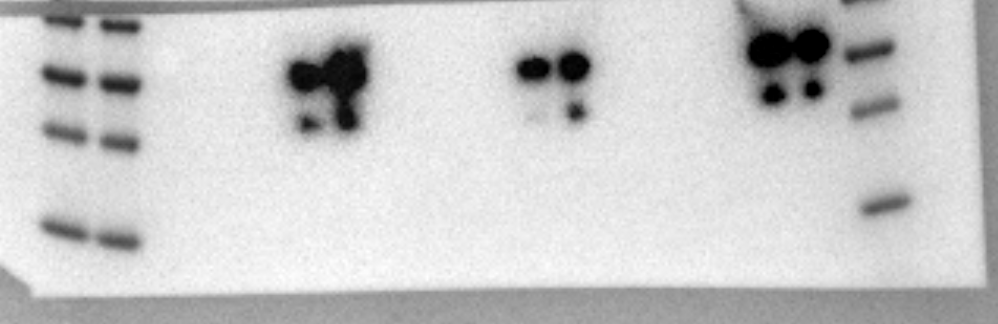

Supplement: Figure 5—source data 1. [file elife-85837-fig5-data1.zip › Figure 5-source data/Figure 5B-3.tif]
