# Supplementary figures and images for: Unconventional secretion of α-synuclein mediated by palmitoylated DNAJC5 oligomers (part 2 of 2)
Source: eLife. 2023 Jan 10;12:e85837. doi: 10.7554/eLife.85837 (PMC9876576; doi:10.7554/eLife.85837)

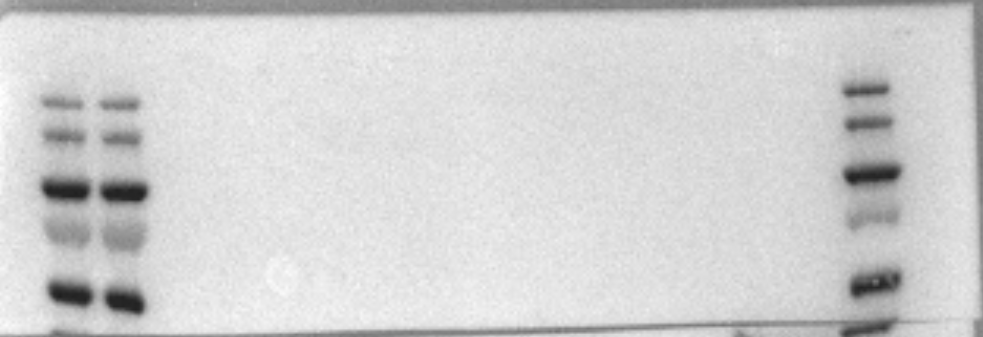

Supplement: Figure 5—source data 1. [file elife-85837-fig5-data1.zip › Figure 5-source data/Figure 5B-4.tif]

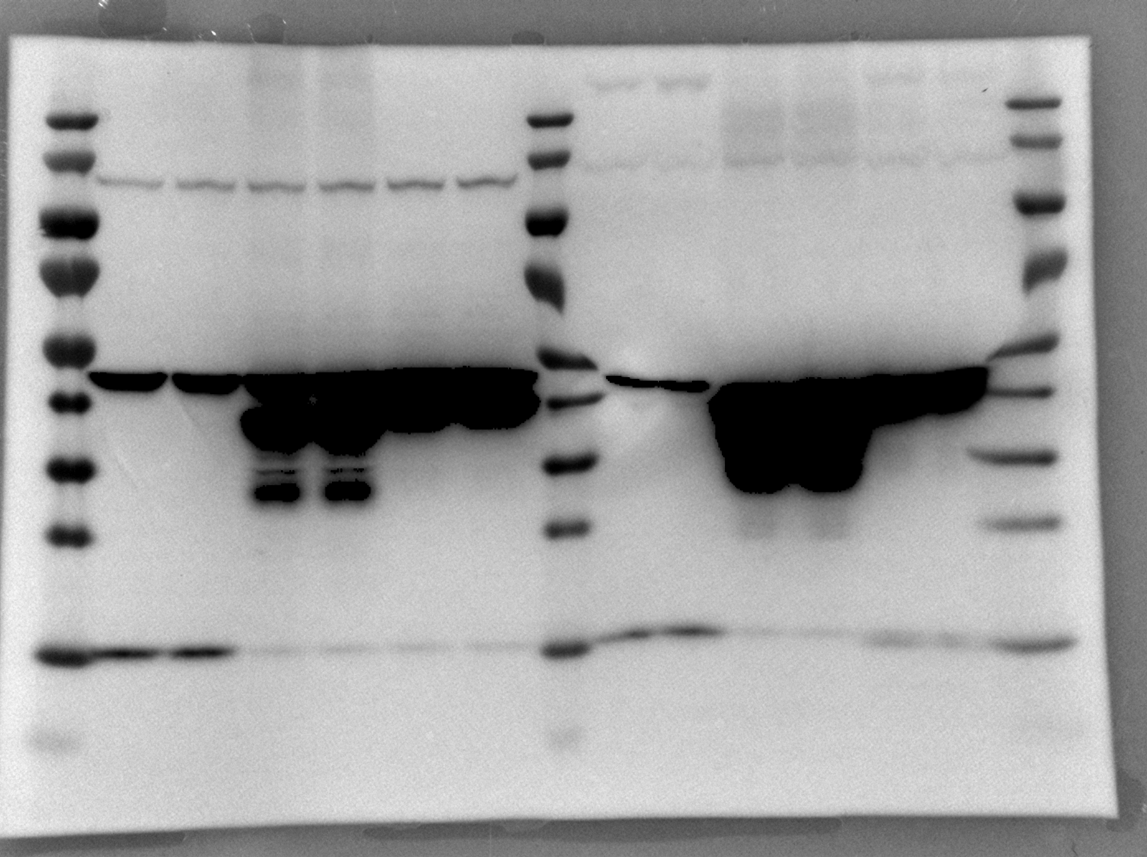

Supplement: Figure 5—source data 1. [file elife-85837-fig5-data1.zip › Figure 5-source data/Figure 5EF-2.tif]

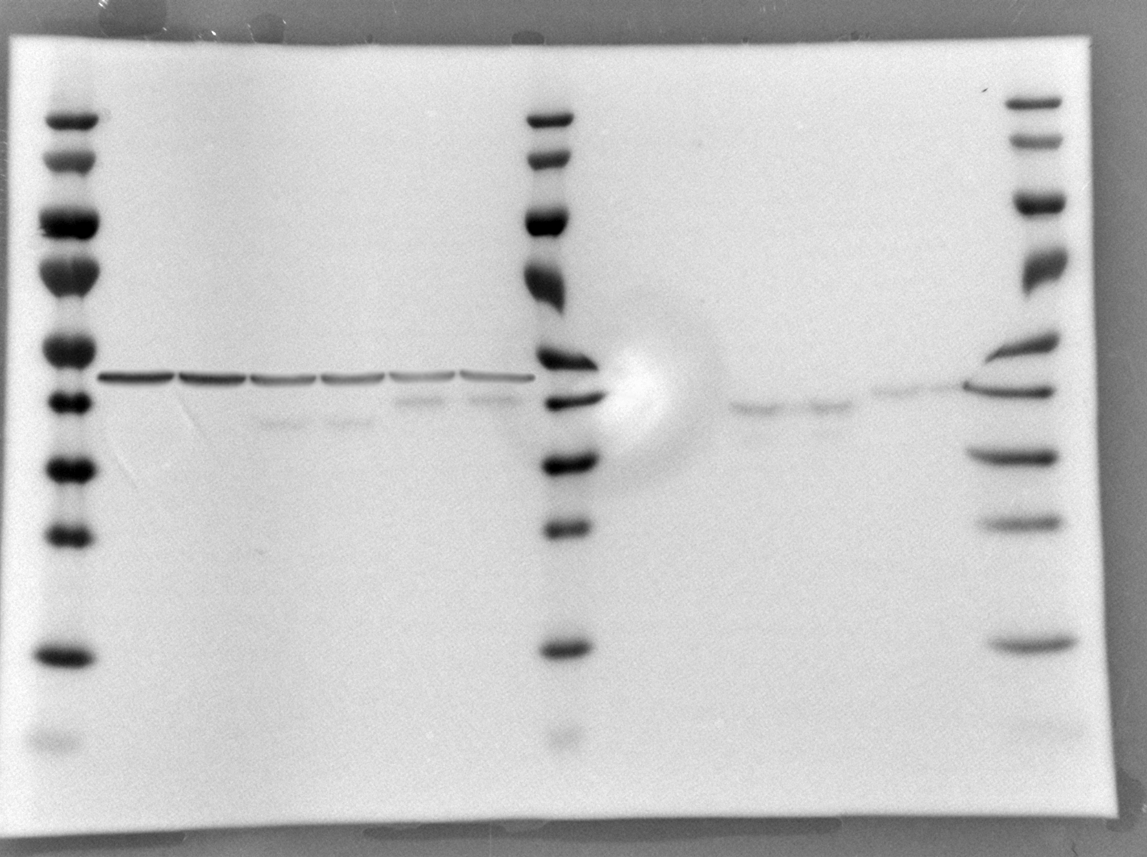

Supplement: Figure 5—source data 1. [file elife-85837-fig5-data1.zip › Figure 5-source data/Figure 5EF-3.tif]

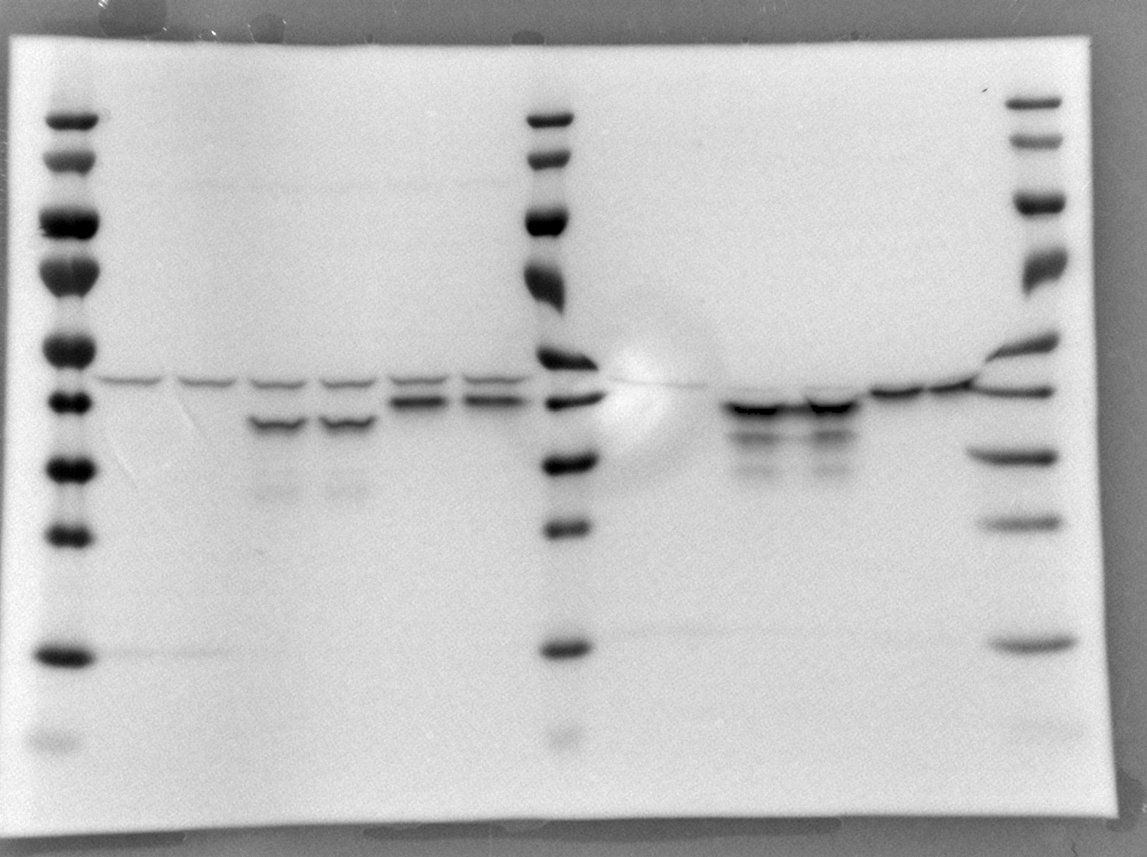

Supplement: Figure 5—source data 1. [file elife-85837-fig5-data1.zip › Figure 5-source data/Figure 5EF-1.tif]

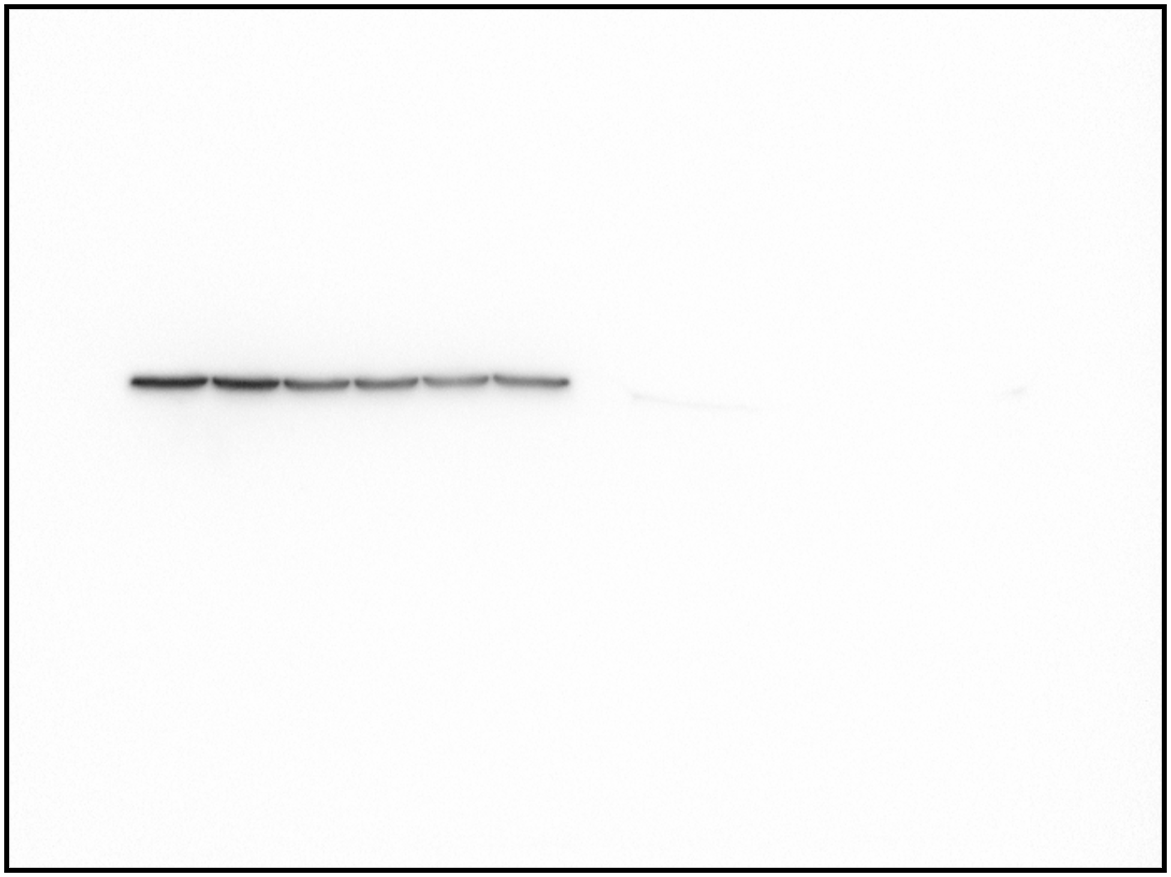

Supplement: Figure 5—source data 1. [file elife-85837-fig5-data1.zip › Figure 5-source data/Figure 5EF-4.tif]

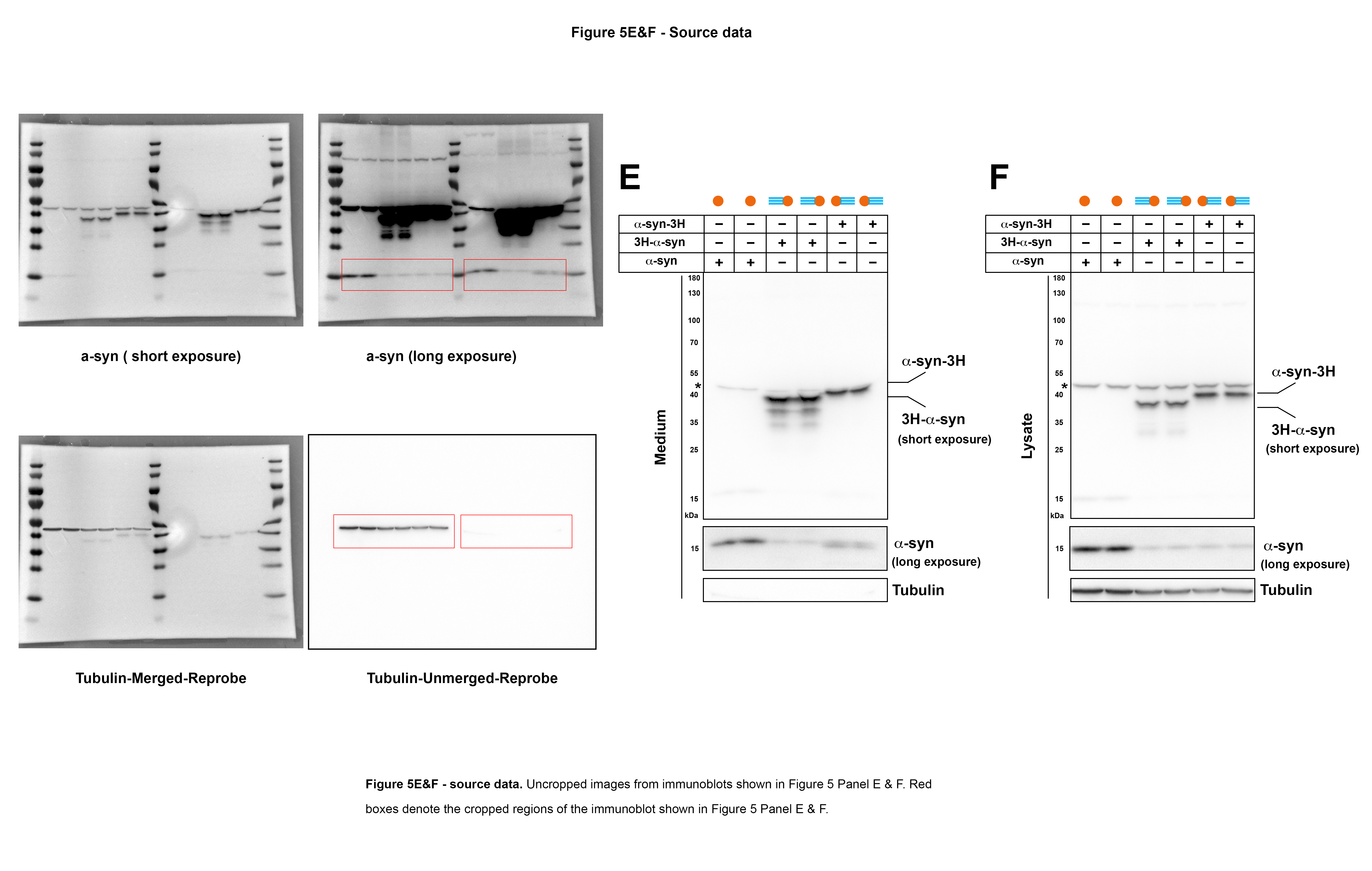

Supplement: Figure 5—source data 1. [file elife-85837-fig5-data1.zip › Figure 5-source data/Figure 5EF-source data.tif]

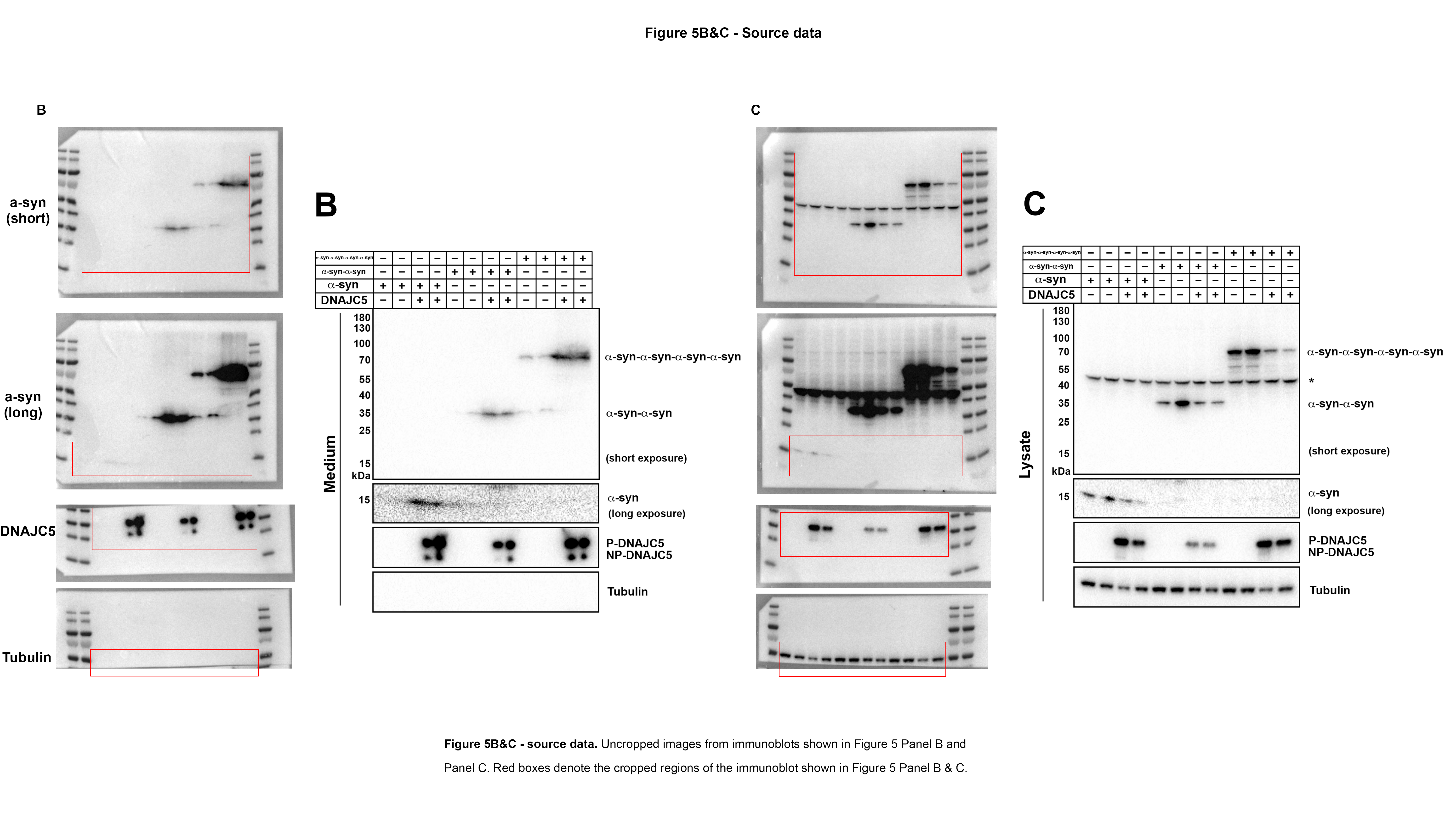

Supplement: Figure 5—source data 1. [file elife-85837-fig5-data1.zip › Figure 5-source data/Figure 5BC-source data.tif]

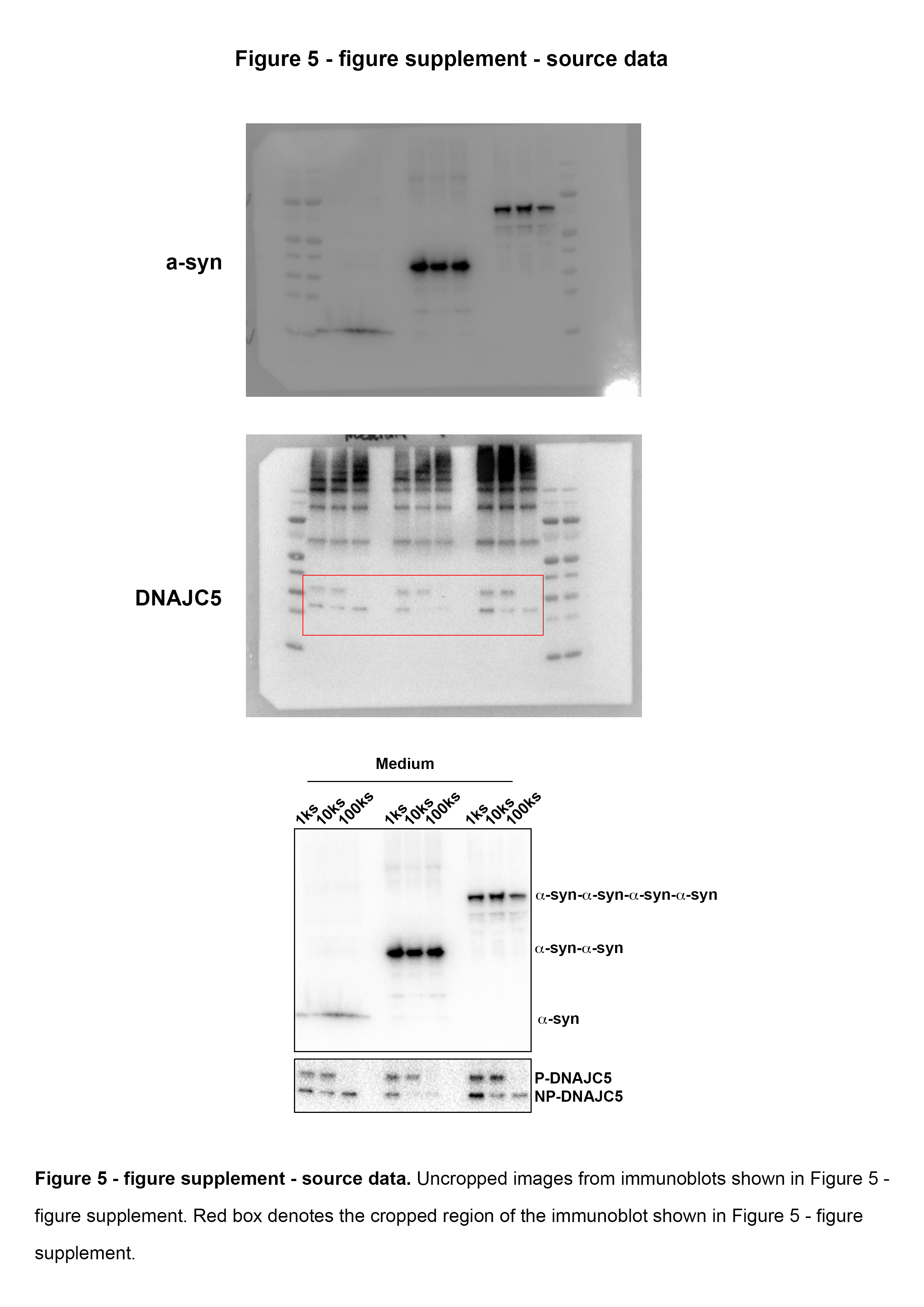

Supplement: Figure 5—figure supplement 1—source data 1. [file elife-85837-fig5-figsupp1-data1.zip › Figure 5-figure supplement 1-source data/Figure 5-figure supplement 1-source data.tif]

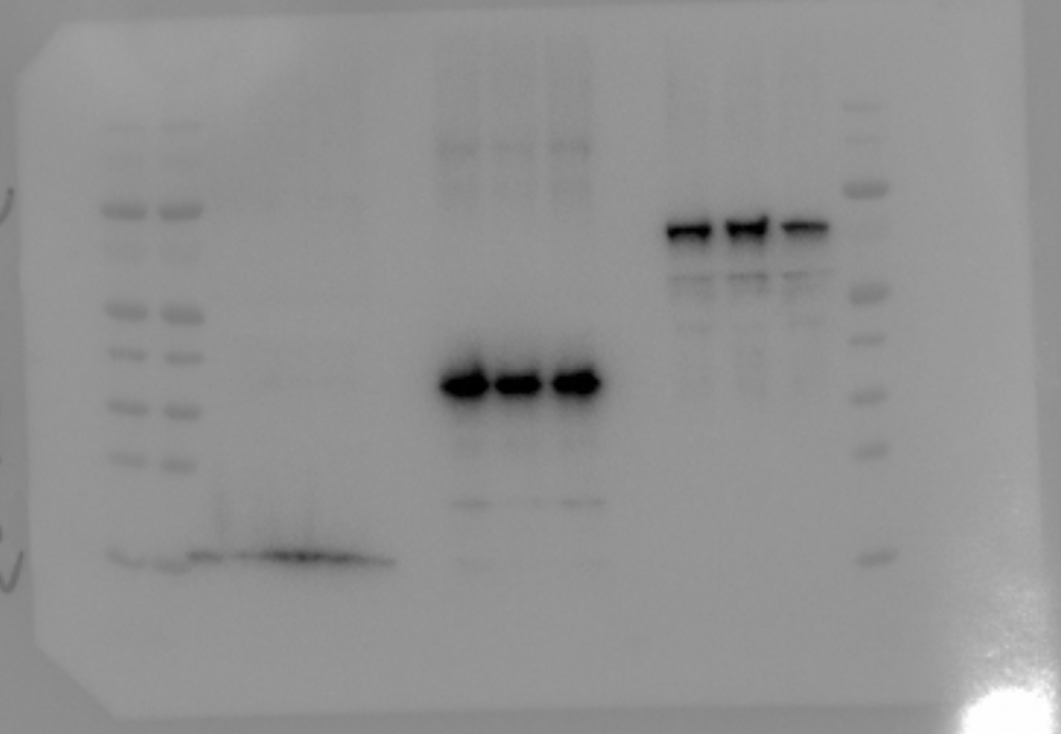

Supplement: Figure 5—figure supplement 1—source data 1. [file elife-85837-fig5-figsupp1-data1.zip › Figure 5-figure supplement 1-source data/Figure 5-figure supplement 1-1.tif]

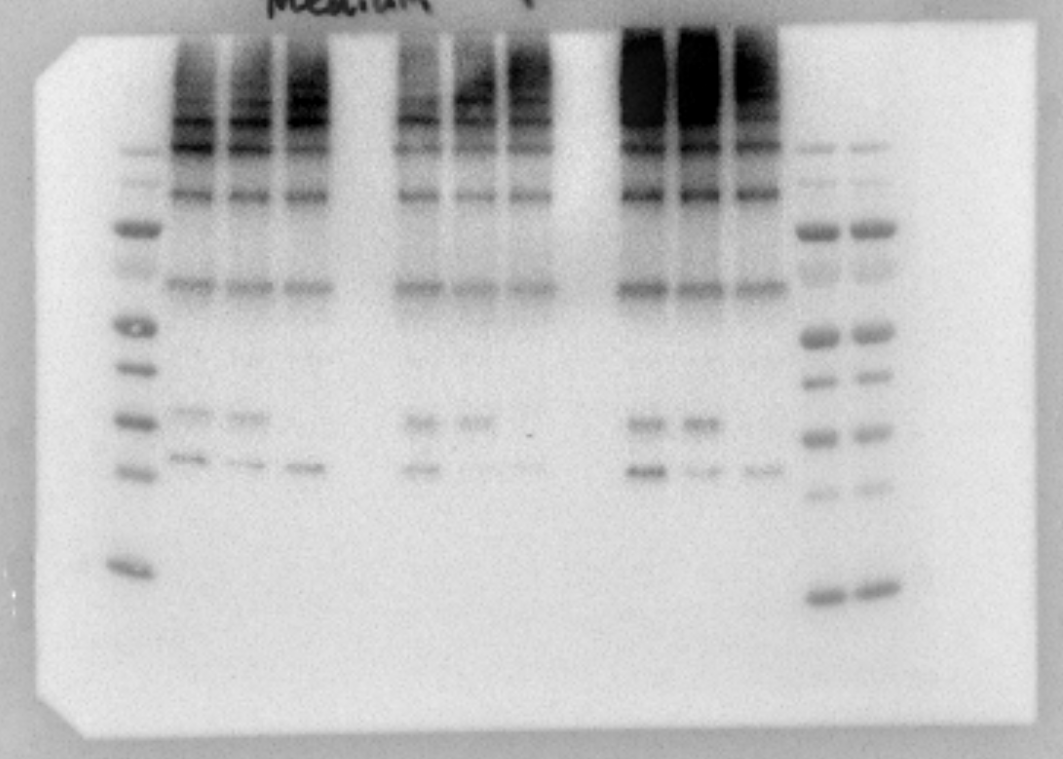

Supplement: Figure 5—figure supplement 1—source data 1. [file elife-85837-fig5-figsupp1-data1.zip › Figure 5-figure supplement 1-source data/Figure 5-figure supplement 1-2.tif]

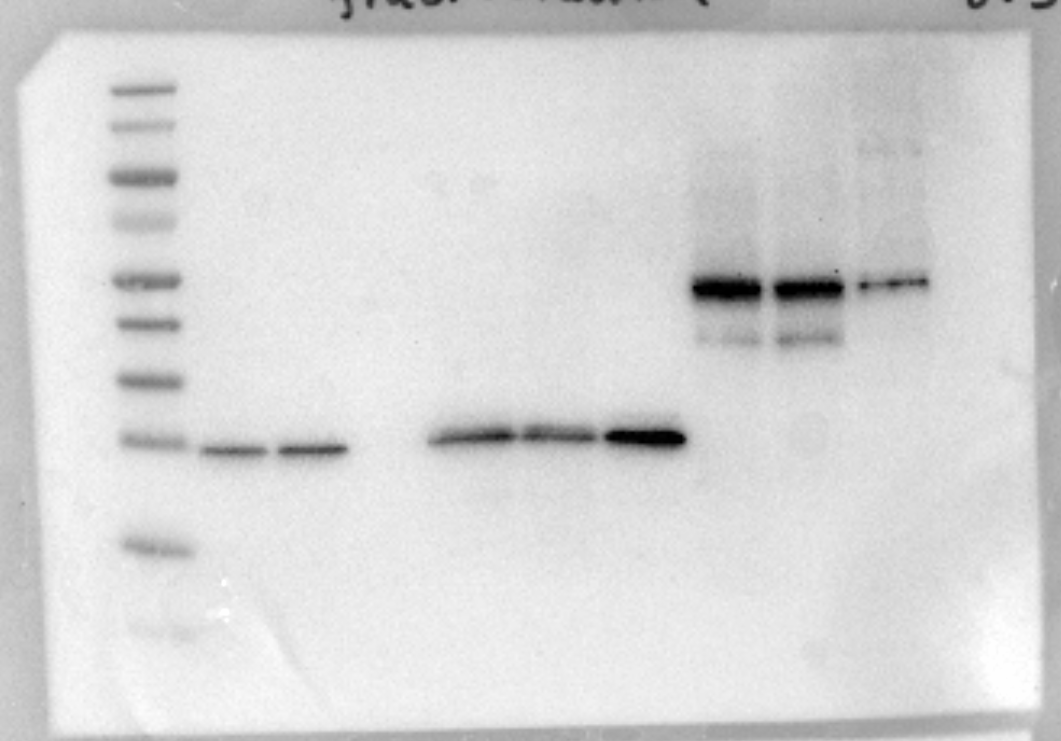

Supplement: Figure 5—figure supplement 2—source data 1. [file elife-85837-fig5-figsupp2-data1.zip › Figure 5-figure supplement 2-source data/Figure 5-figure supplement 2B-1.tif]

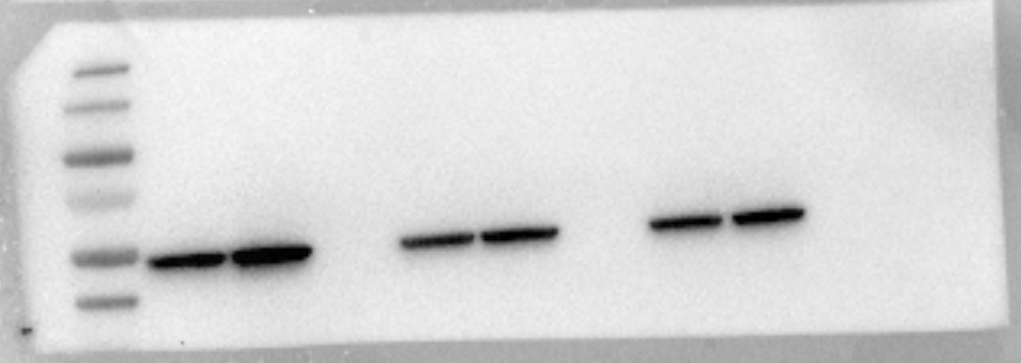

Supplement: Figure 5—figure supplement 2—source data 1. [file elife-85837-fig5-figsupp2-data1.zip › Figure 5-figure supplement 2-source data/Figure 5-figure supplement 2B-2.tif]

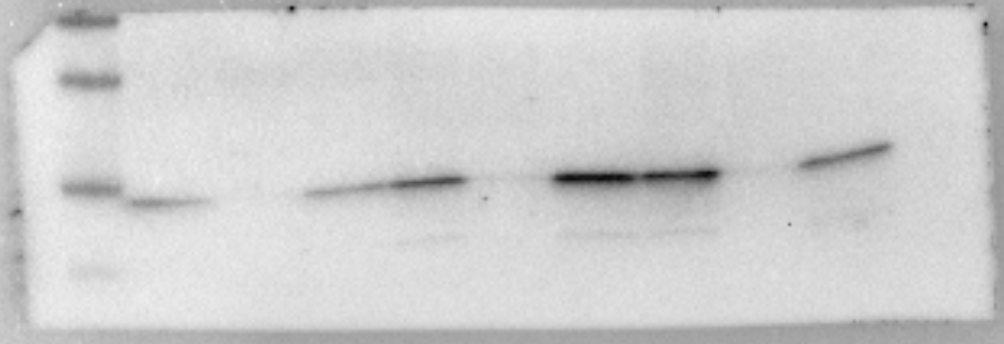

Supplement: Figure 5—figure supplement 2—source data 1. [file elife-85837-fig5-figsupp2-data1.zip › Figure 5-figure supplement 2-source data/Figure 5-figure supplement 2B-3.tif]

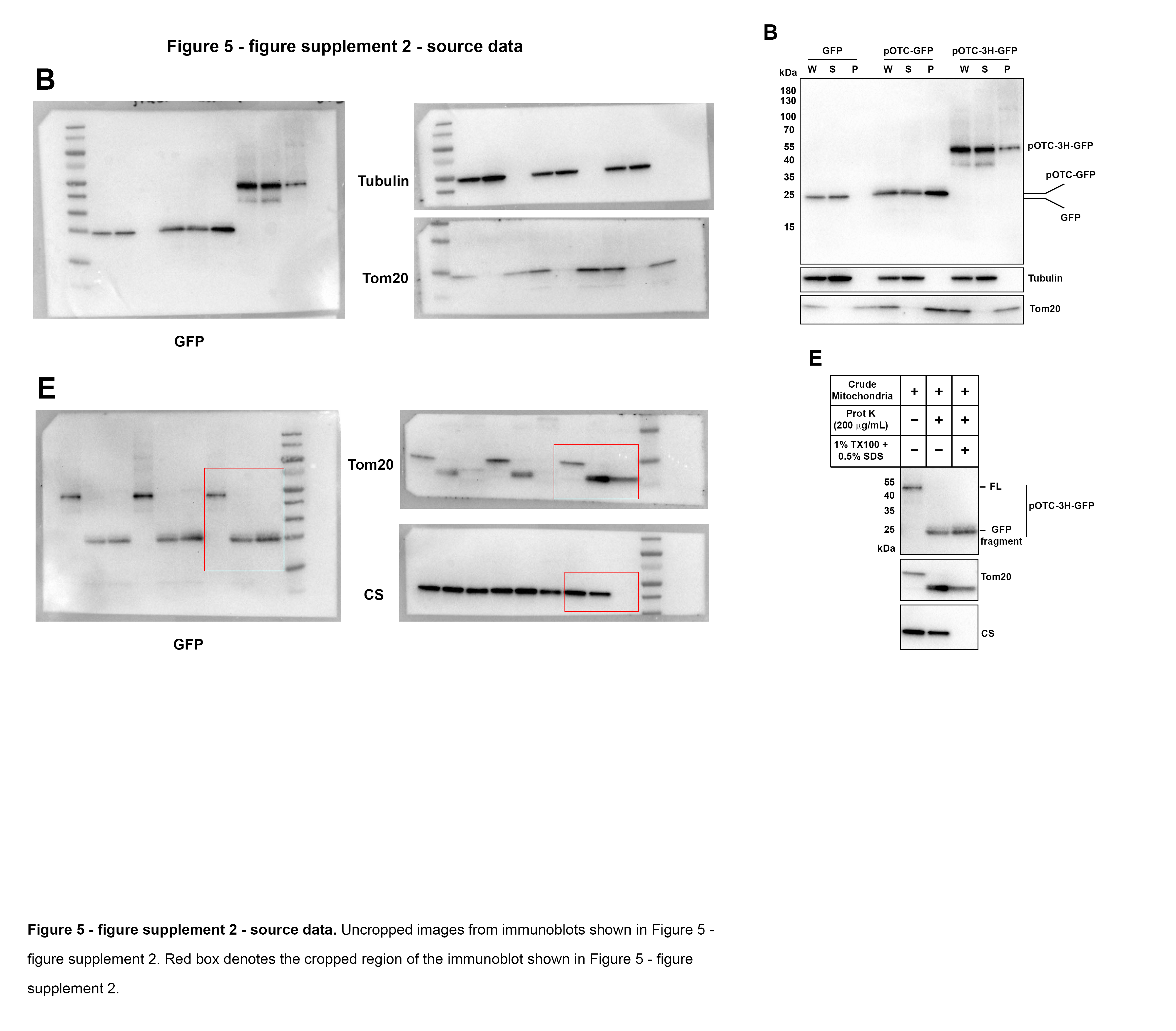

Supplement: Figure 5—figure supplement 2—source data 1. [file elife-85837-fig5-figsupp2-data1.zip › Figure 5-figure supplement 2-source data/Figure 5-figure supplement 2-source data.tif]

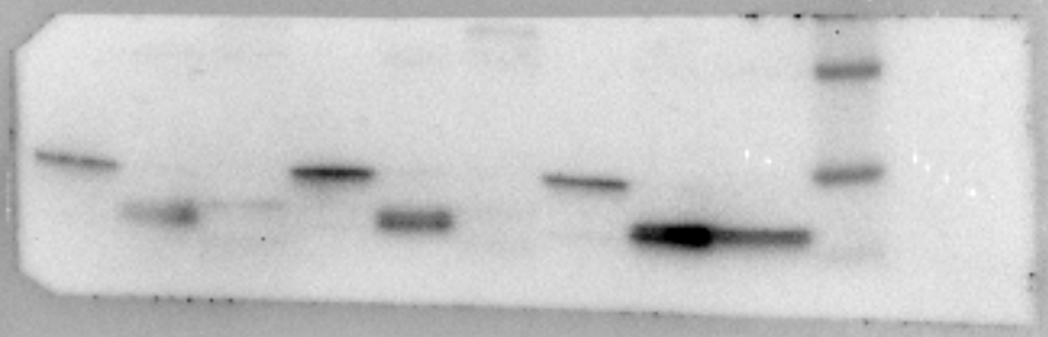

Supplement: Figure 5—figure supplement 2—source data 1. [file elife-85837-fig5-figsupp2-data1.zip › Figure 5-figure supplement 2-source data/Figure 5-figure supplement 2E-2.tif]

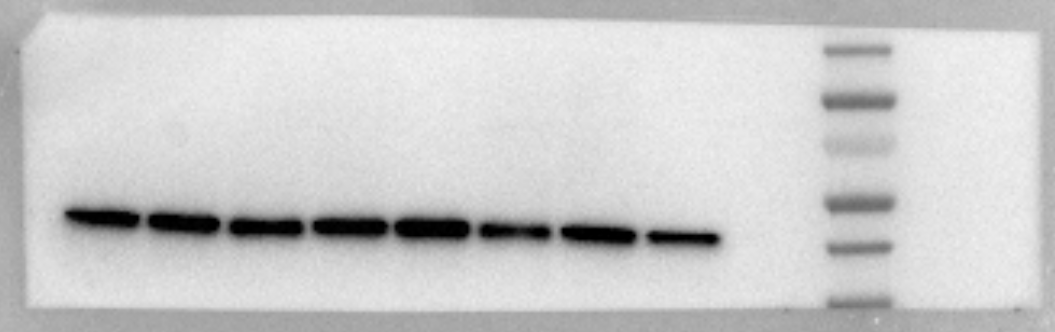

Supplement: Figure 5—figure supplement 2—source data 1. [file elife-85837-fig5-figsupp2-data1.zip › Figure 5-figure supplement 2-source data/Figure 5-figure supplement 2E-3.tif]

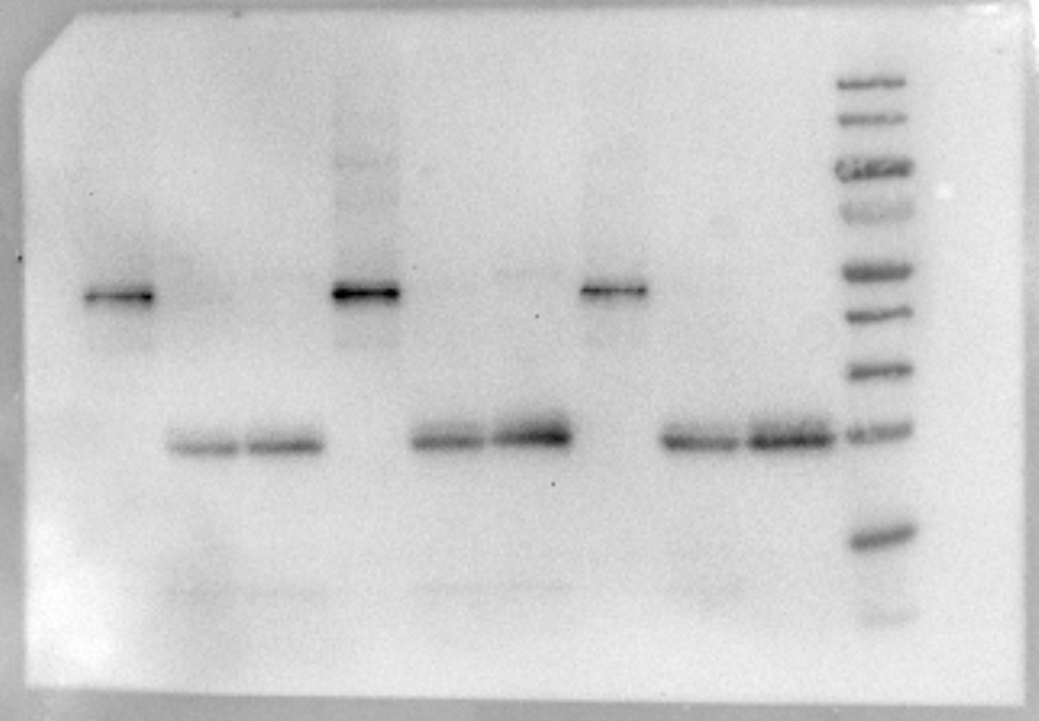

Supplement: Figure 5—figure supplement 2—source data 1. [file elife-85837-fig5-figsupp2-data1.zip › Figure 5-figure supplement 2-source data/Figure 5-figure supplement 2E-1.tif]

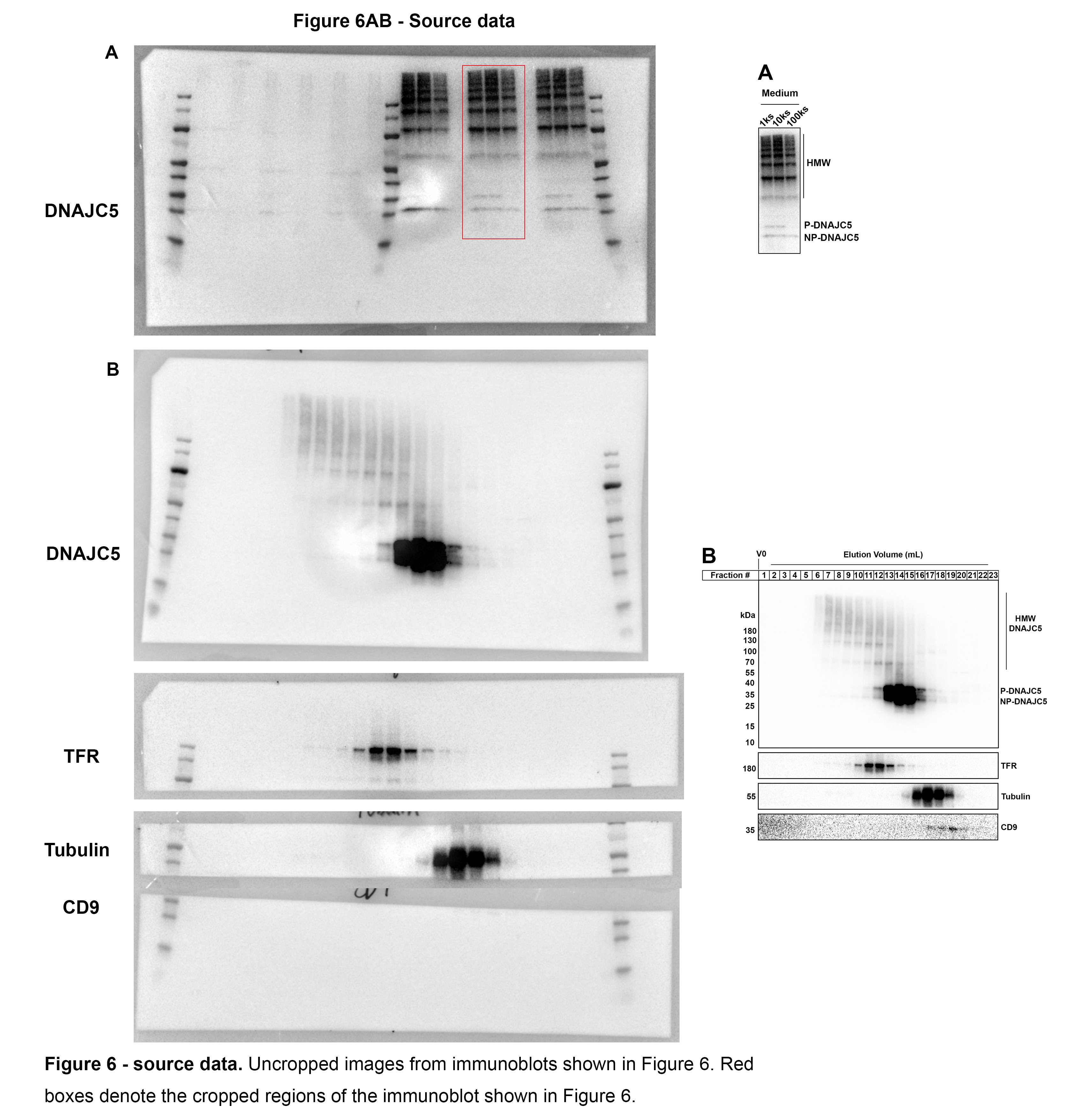

Supplement: Figure 6—source data 1. [file elife-85837-fig6-data1.zip › Figure 6-source data/Figure 6AB-source data.tif]

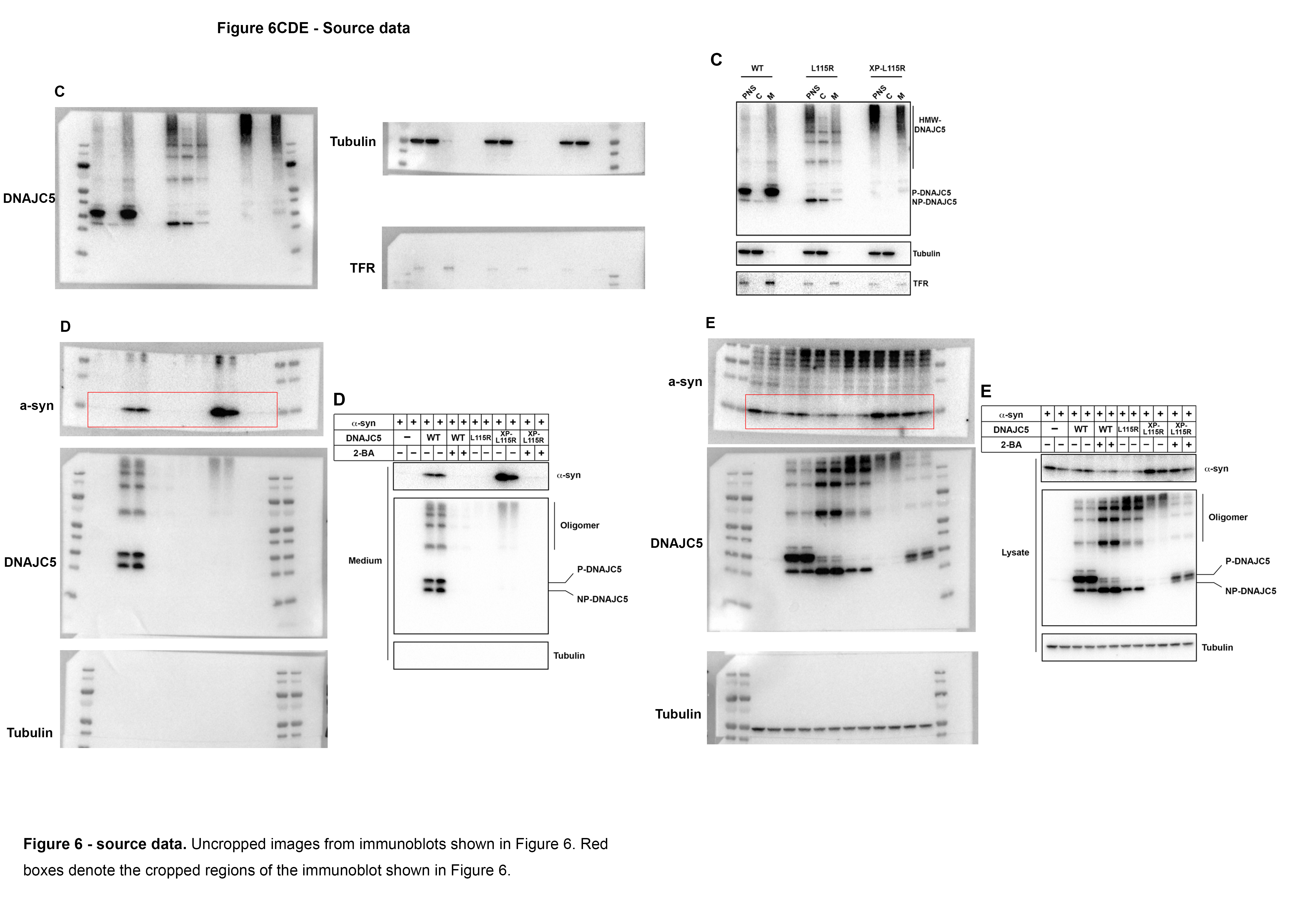

Supplement: Figure 6—source data 1. [file elife-85837-fig6-data1.zip › Figure 6-source data/Figure 6CDE-source data.tif]

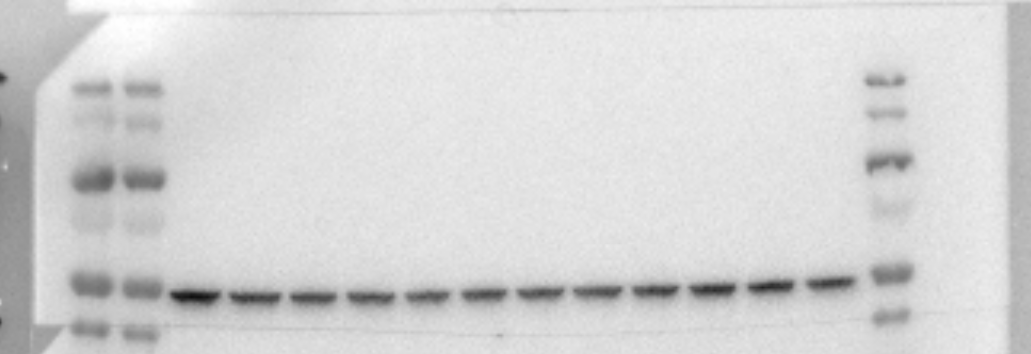

Supplement: Figure 6—source data 1. [file elife-85837-fig6-data1.zip › Figure 6-source data/Figure 6E-3.tif]

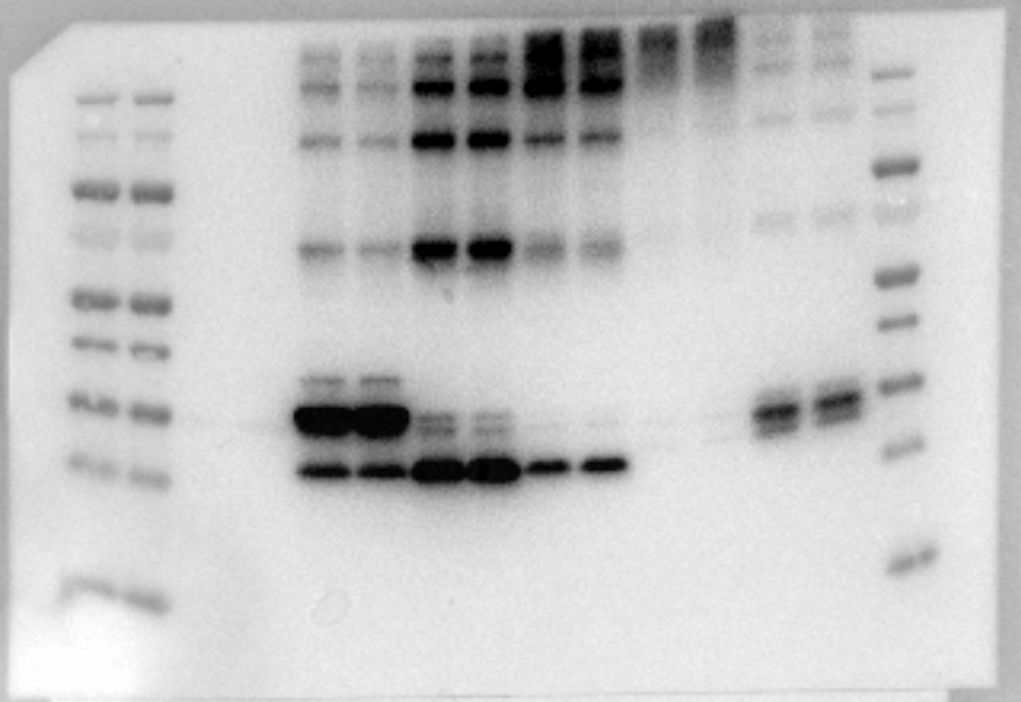

Supplement: Figure 6—source data 1. [file elife-85837-fig6-data1.zip › Figure 6-source data/Figure 6E-2.tif]

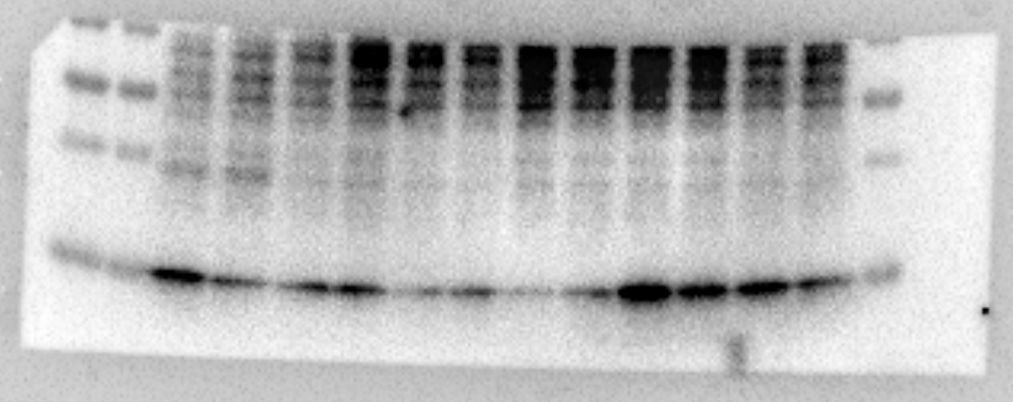

Supplement: Figure 6—source data 1. [file elife-85837-fig6-data1.zip › Figure 6-source data/Figure 6E-1.tif]

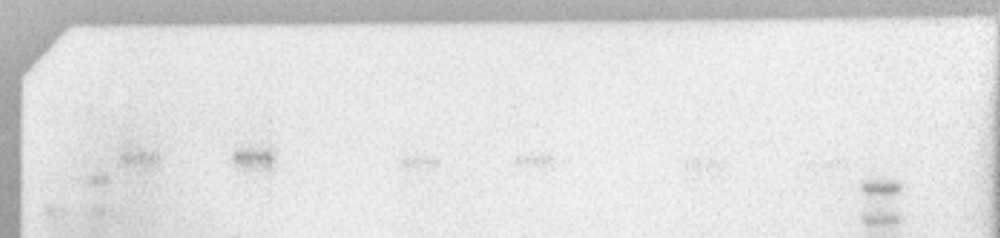

Supplement: Figure 6—source data 1. [file elife-85837-fig6-data1.zip › Figure 6-source data/Figure 6C-3.tif]

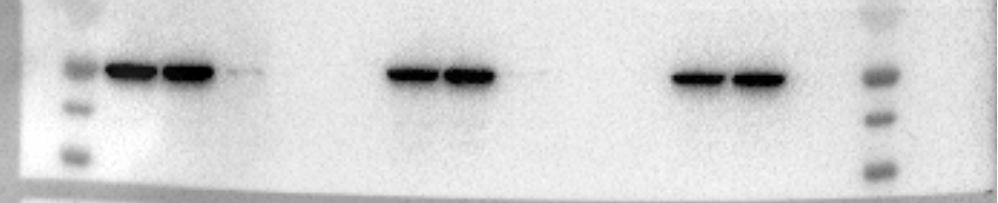

Supplement: Figure 6—source data 1. [file elife-85837-fig6-data1.zip › Figure 6-source data/Figure 6C-2.tif]

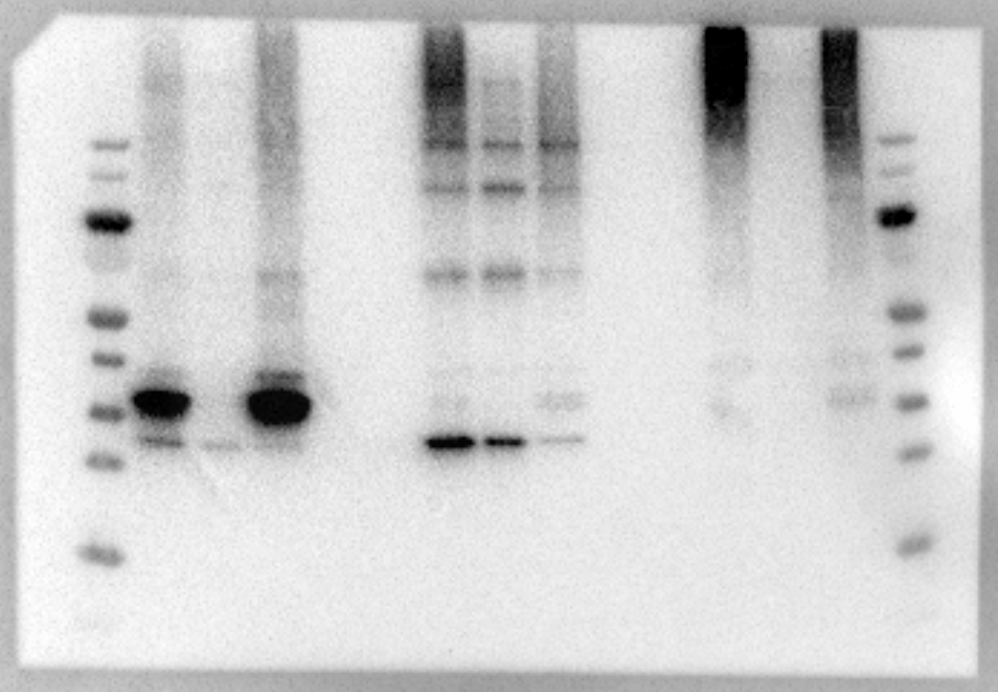

Supplement: Figure 6—source data 1. [file elife-85837-fig6-data1.zip › Figure 6-source data/Figure 6C-1.tif]

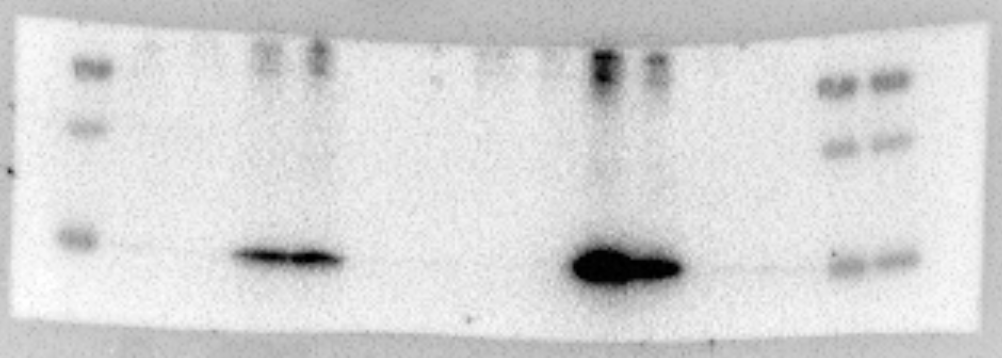

Supplement: Figure 6—source data 1. [file elife-85837-fig6-data1.zip › Figure 6-source data/Figure 6D-1.tif]

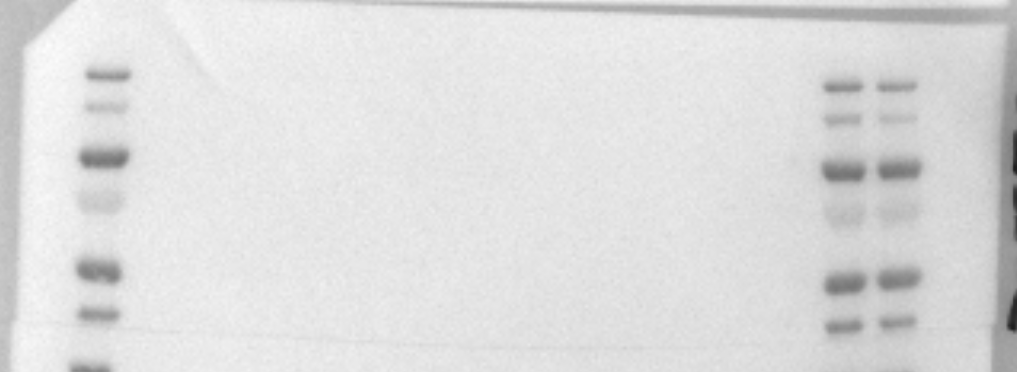

Supplement: Figure 6—source data 1. [file elife-85837-fig6-data1.zip › Figure 6-source data/Figure 6D-3.tif]

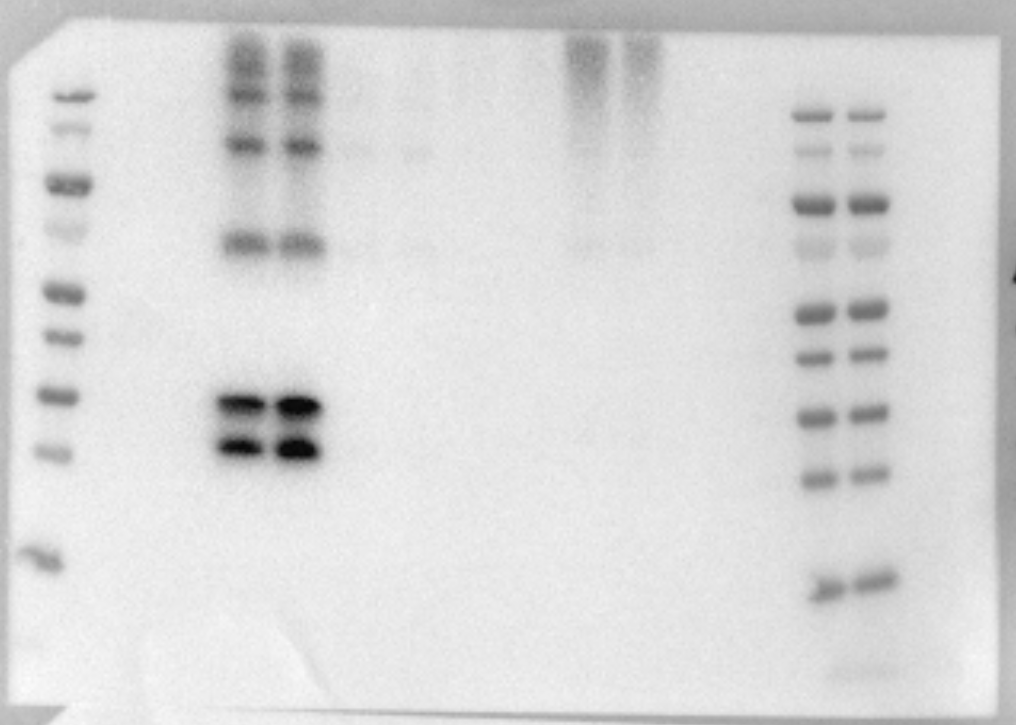

Supplement: Figure 6—source data 1. [file elife-85837-fig6-data1.zip › Figure 6-source data/Figure 6D-2.tif]

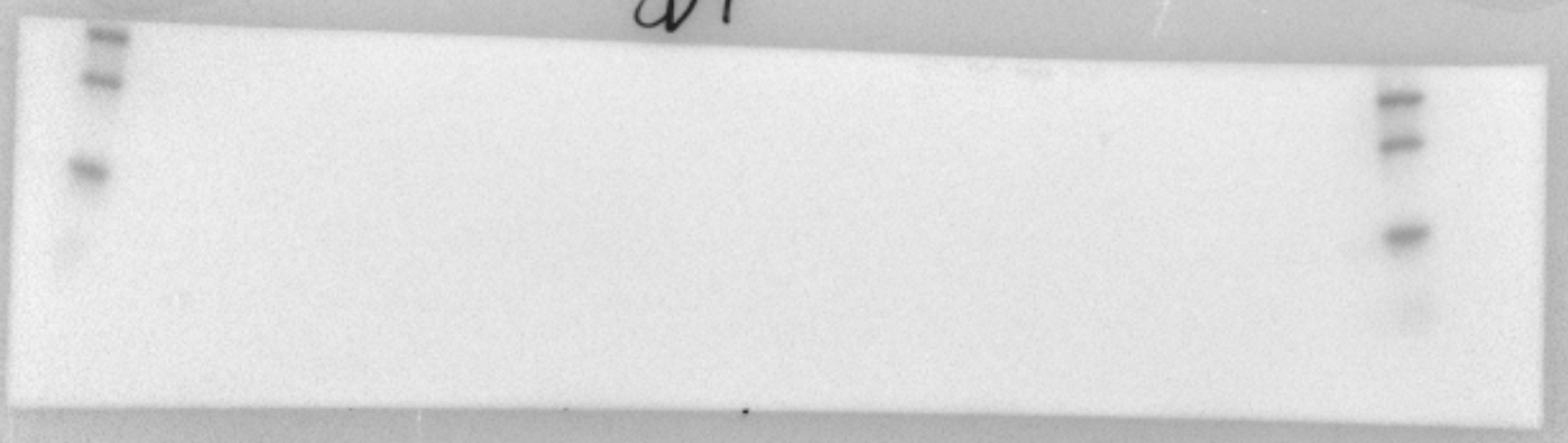

Supplement: Figure 6—source data 1. [file elife-85837-fig6-data1.zip › Figure 6-source data/Figure 6B-4.tif]

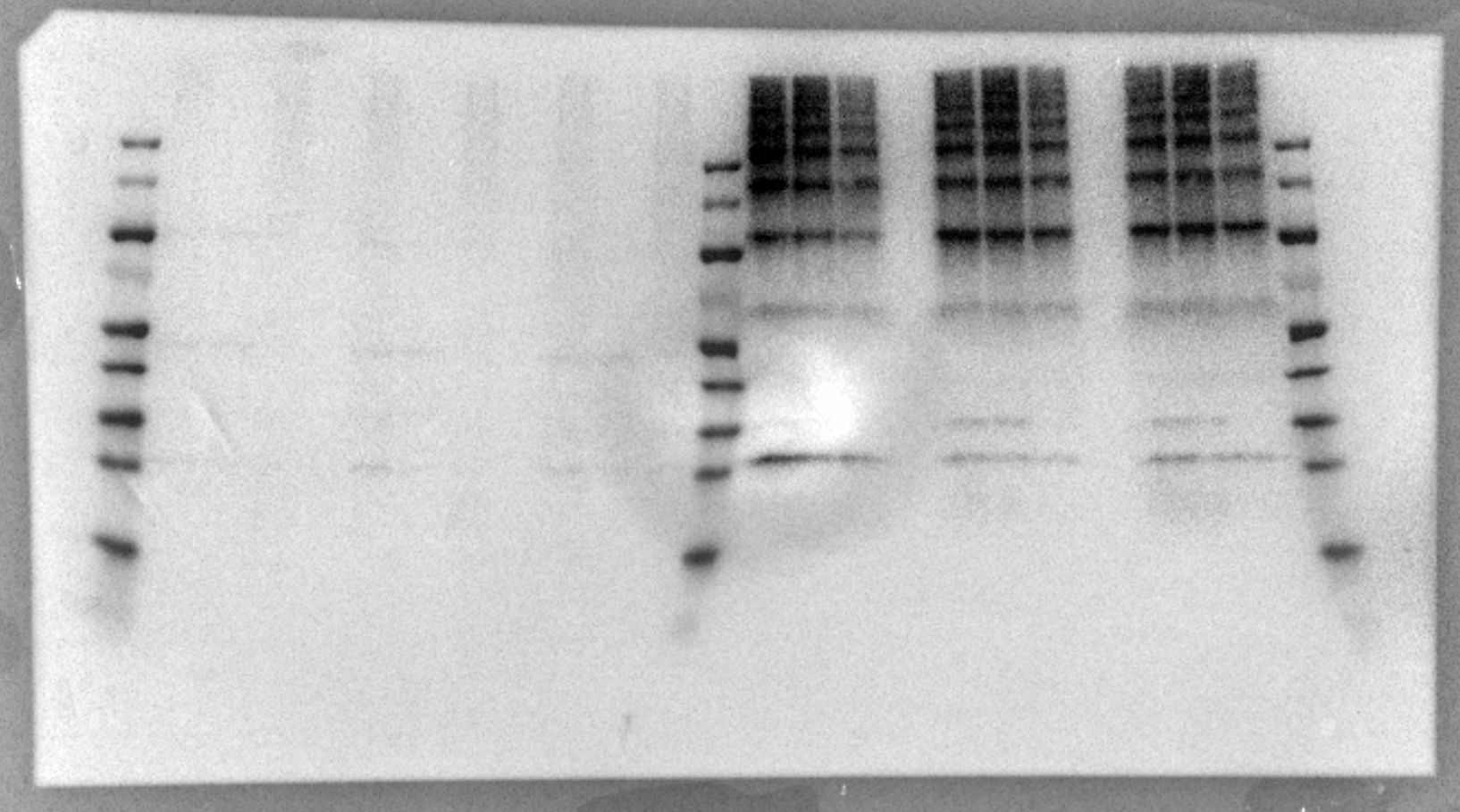

Supplement: Figure 6—source data 1. [file elife-85837-fig6-data1.zip › Figure 6-source data/Figure 6A.tif]

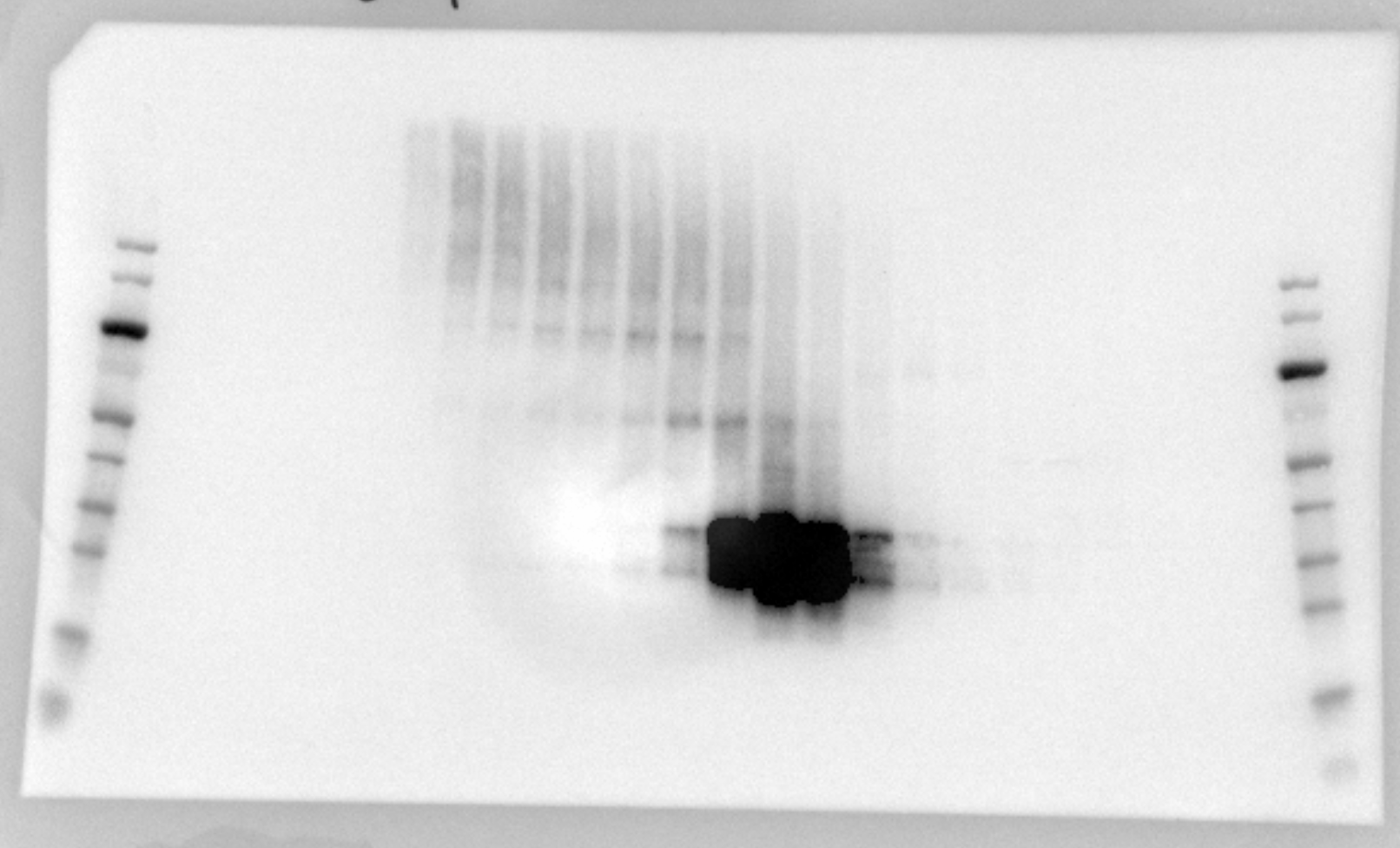

Supplement: Figure 6—source data 1. [file elife-85837-fig6-data1.zip › Figure 6-source data/Figure 6B-1.tif]

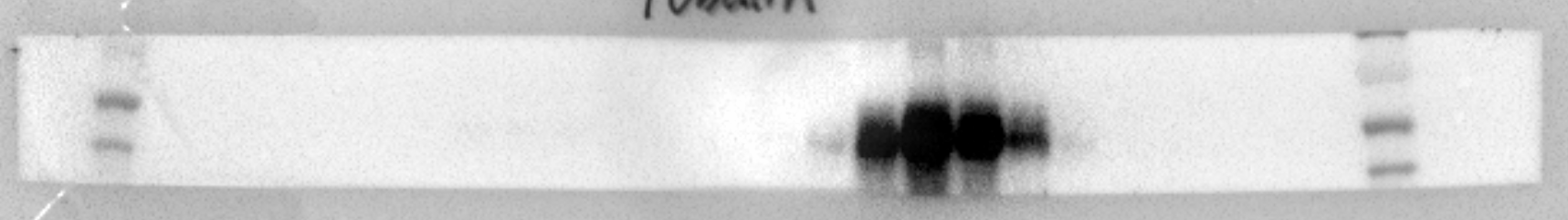

Supplement: Figure 6—source data 1. [file elife-85837-fig6-data1.zip › Figure 6-source data/Figure 6B-3.tif]

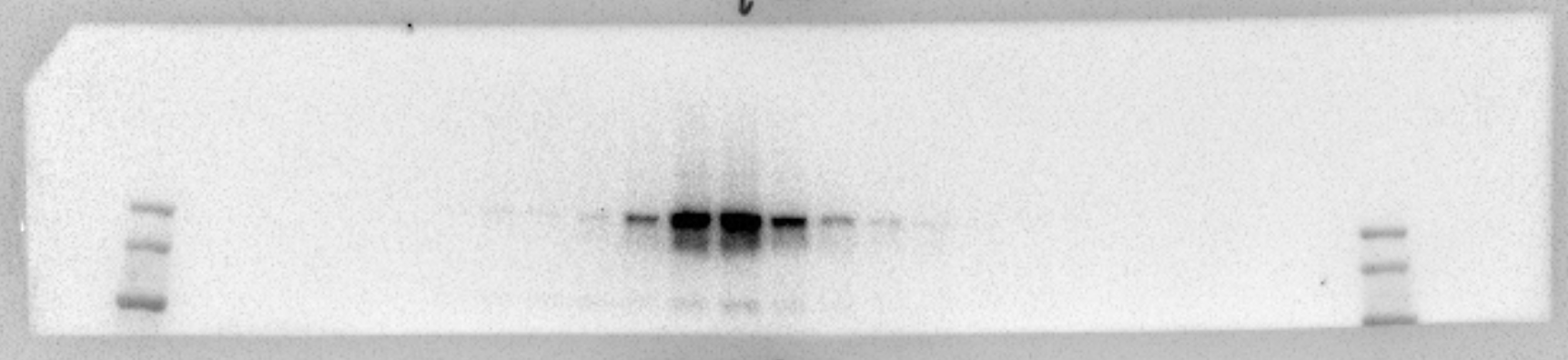

Supplement: Figure 6—source data 1. [file elife-85837-fig6-data1.zip › Figure 6-source data/Figure 6B-2.tif]

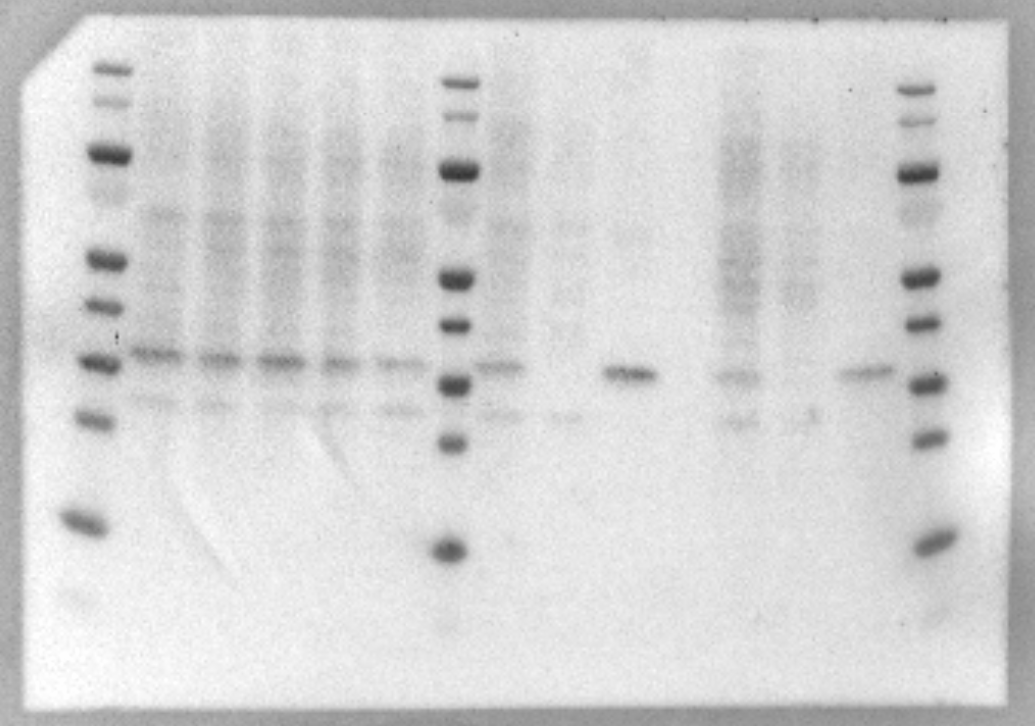

Supplement: Figure 6—figure supplement 1—source data 1. [file elife-85837-fig6-figsupp1-data1.zip › Figure 6-figure supplement 1-source data/Figure 6-figure supplement 1B-1.tif]

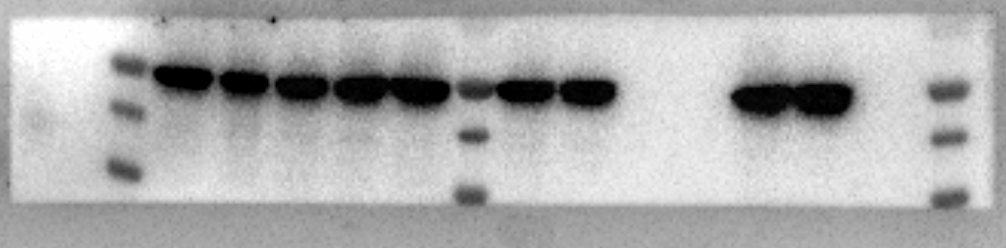

Supplement: Figure 6—figure supplement 1—source data 1. [file elife-85837-fig6-figsupp1-data1.zip › Figure 6-figure supplement 1-source data/Figure 6-figure supplement 1B-2.tif]

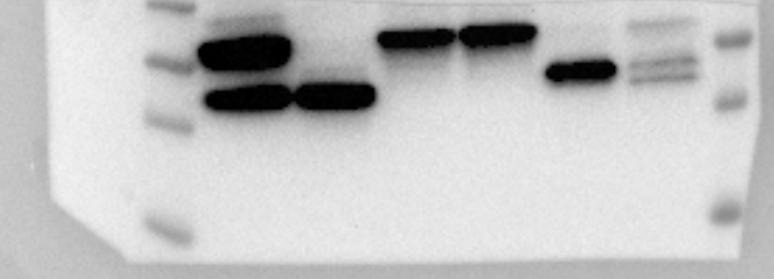

Supplement: Figure 6—figure supplement 1—source data 1. [file elife-85837-fig6-figsupp1-data1.zip › Figure 6-figure supplement 1-source data/Figure 6-figure supplement 1D-1.tif]

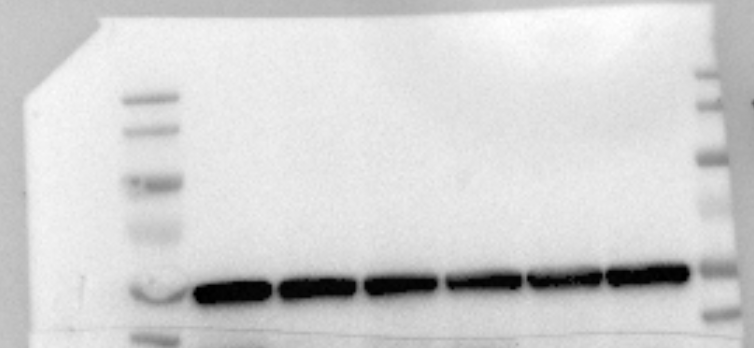

Supplement: Figure 6—figure supplement 1—source data 1. [file elife-85837-fig6-figsupp1-data1.zip › Figure 6-figure supplement 1-source data/Figure 6-figure supplement 1D-2.tif]

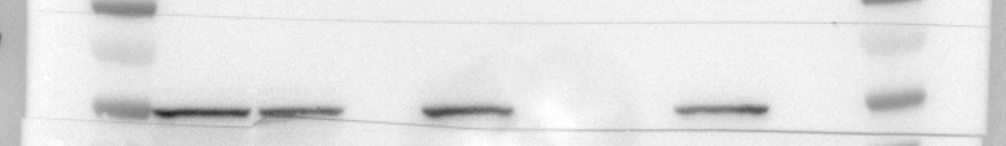

Supplement: Figure 6—figure supplement 1—source data 1. [file elife-85837-fig6-figsupp1-data1.zip › Figure 6-figure supplement 1-source data/Figure 6-figure supplement 1E-2.tif]

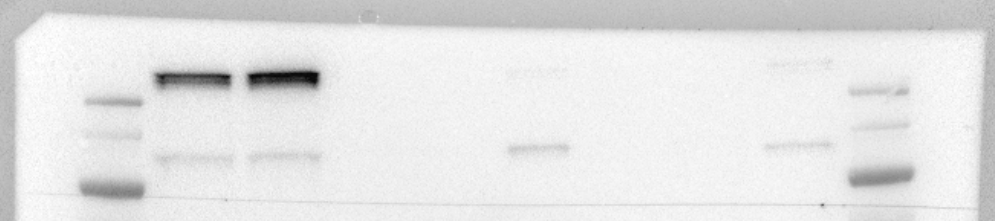

Supplement: Figure 6—figure supplement 1—source data 1. [file elife-85837-fig6-figsupp1-data1.zip › Figure 6-figure supplement 1-source data/Figure 6-figure supplement 1E-3.tif]

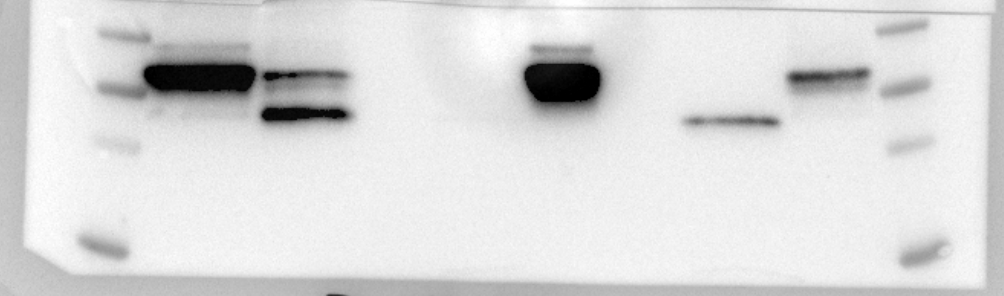

Supplement: Figure 6—figure supplement 1—source data 1. [file elife-85837-fig6-figsupp1-data1.zip › Figure 6-figure supplement 1-source data/Figure 6-figure supplement 1E-1.tif]

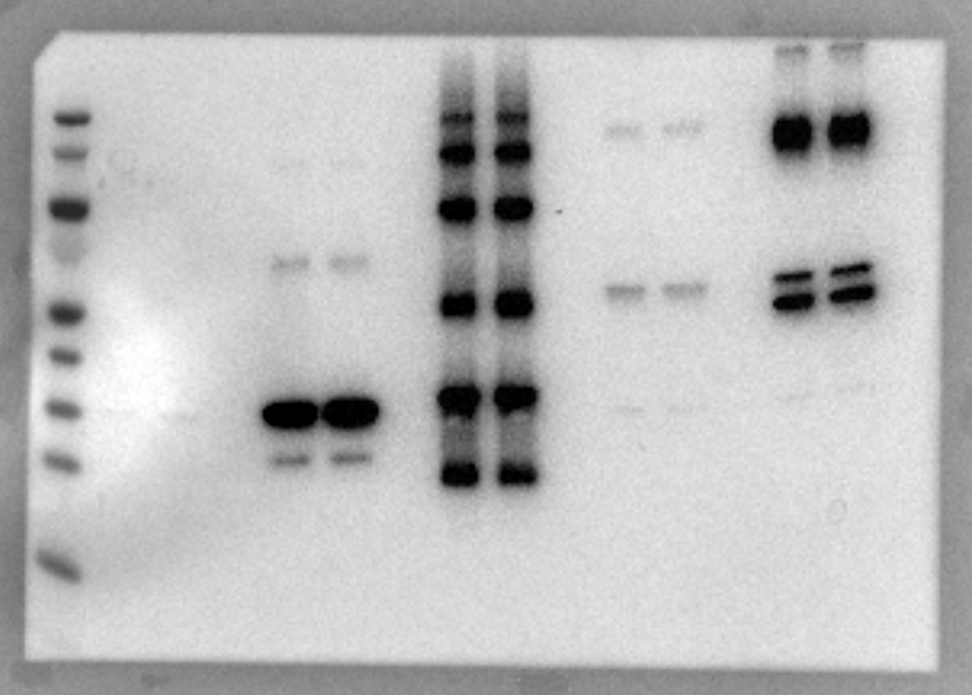

Supplement: Figure 6—figure supplement 1—source data 1. [file elife-85837-fig6-figsupp1-data1.zip › Figure 6-figure supplement 1-source data/Figure 6-figure supplement 1A.tif]

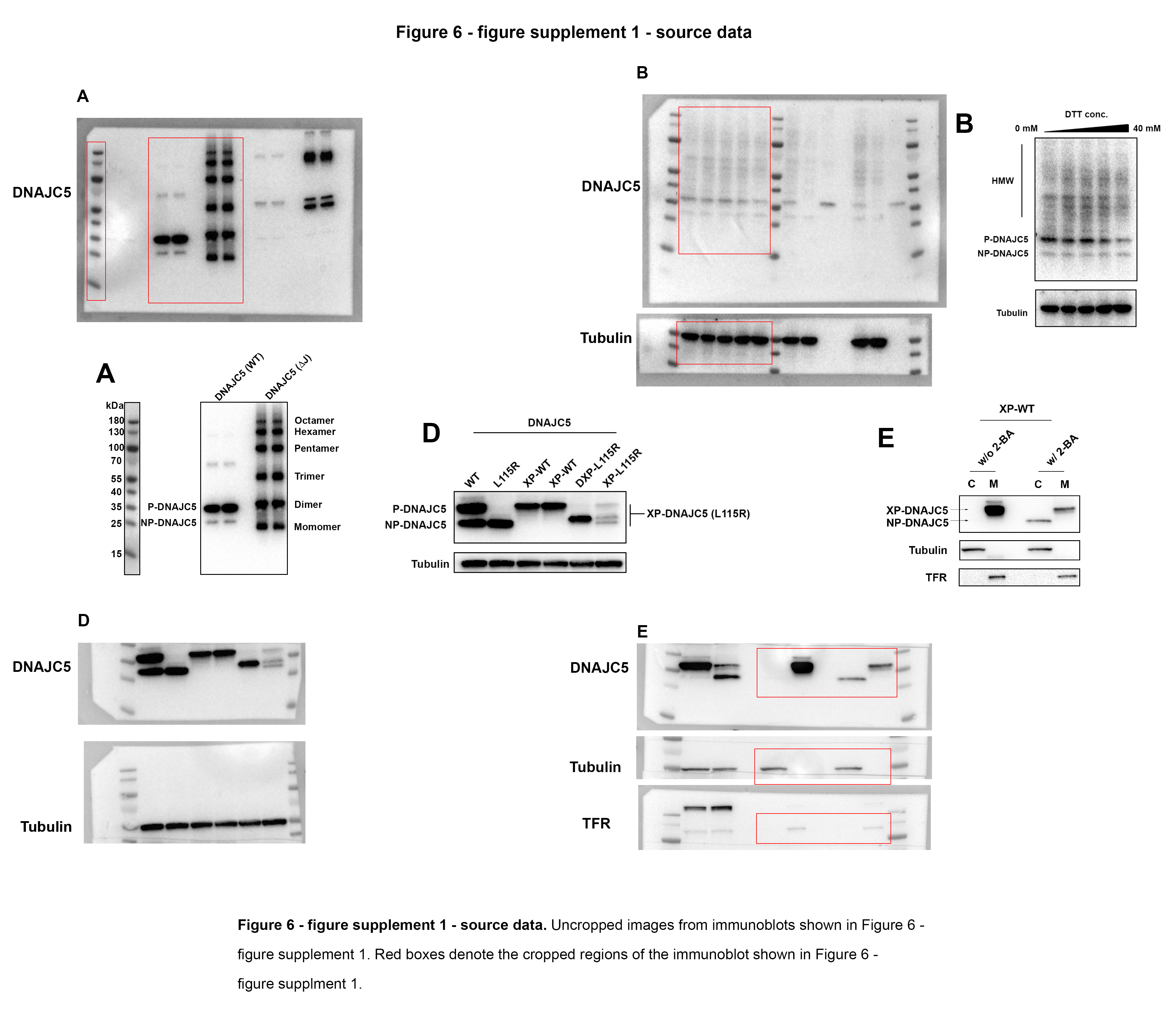

Supplement: Figure 6—figure supplement 1—source data 1. [file elife-85837-fig6-figsupp1-data1.zip › Figure 6-figure supplement 1-source data/Figure 6-figure supplement 1-source data.tif]

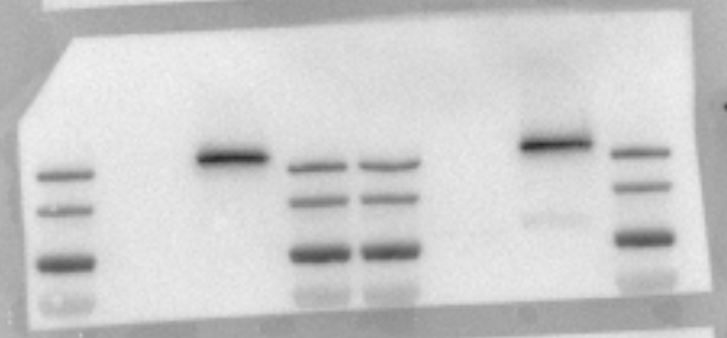

Supplement: Figure 7—source data 1. [file elife-85837-fig7-data1.zip › Figure 7-source data/Figure 7A-4.tif]

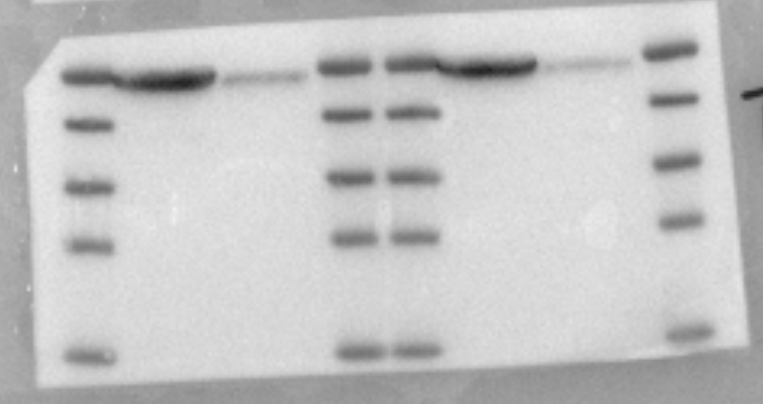

Supplement: Figure 7—source data 1. [file elife-85837-fig7-data1.zip › Figure 7-source data/Figure 7A-3.tif]

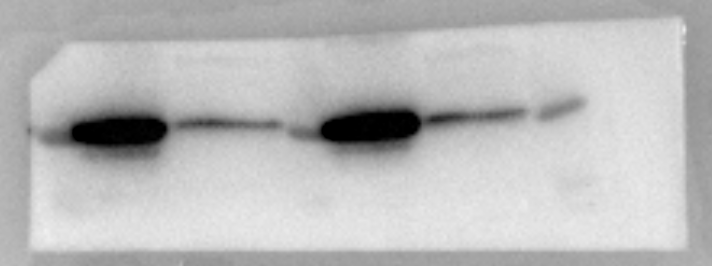

Supplement: Figure 7—source data 1. [file elife-85837-fig7-data1.zip › Figure 7-source data/Figure 7A-2.tif]

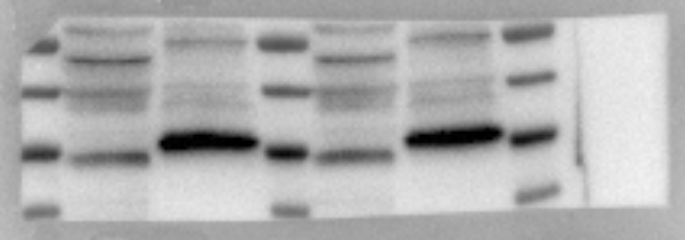

Supplement: Figure 7—source data 1. [file elife-85837-fig7-data1.zip › Figure 7-source data/Figure 7A-1.tif]

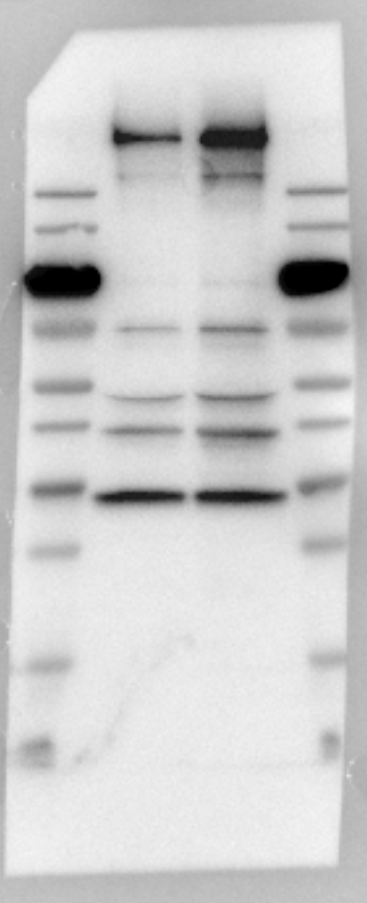

Supplement: Figure 7—source data 1. [file elife-85837-fig7-data1.zip › Figure 7-source data/Figure 7F-1.tif]

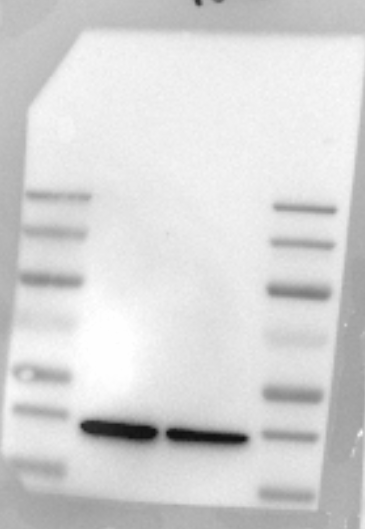

Supplement: Figure 7—source data 1. [file elife-85837-fig7-data1.zip › Figure 7-source data/Figure 7F-3.tif]

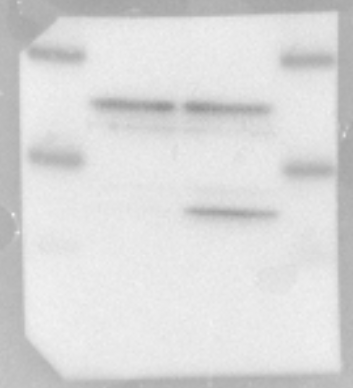

Supplement: Figure 7—source data 1. [file elife-85837-fig7-data1.zip › Figure 7-source data/Figure 7F-2.tif]

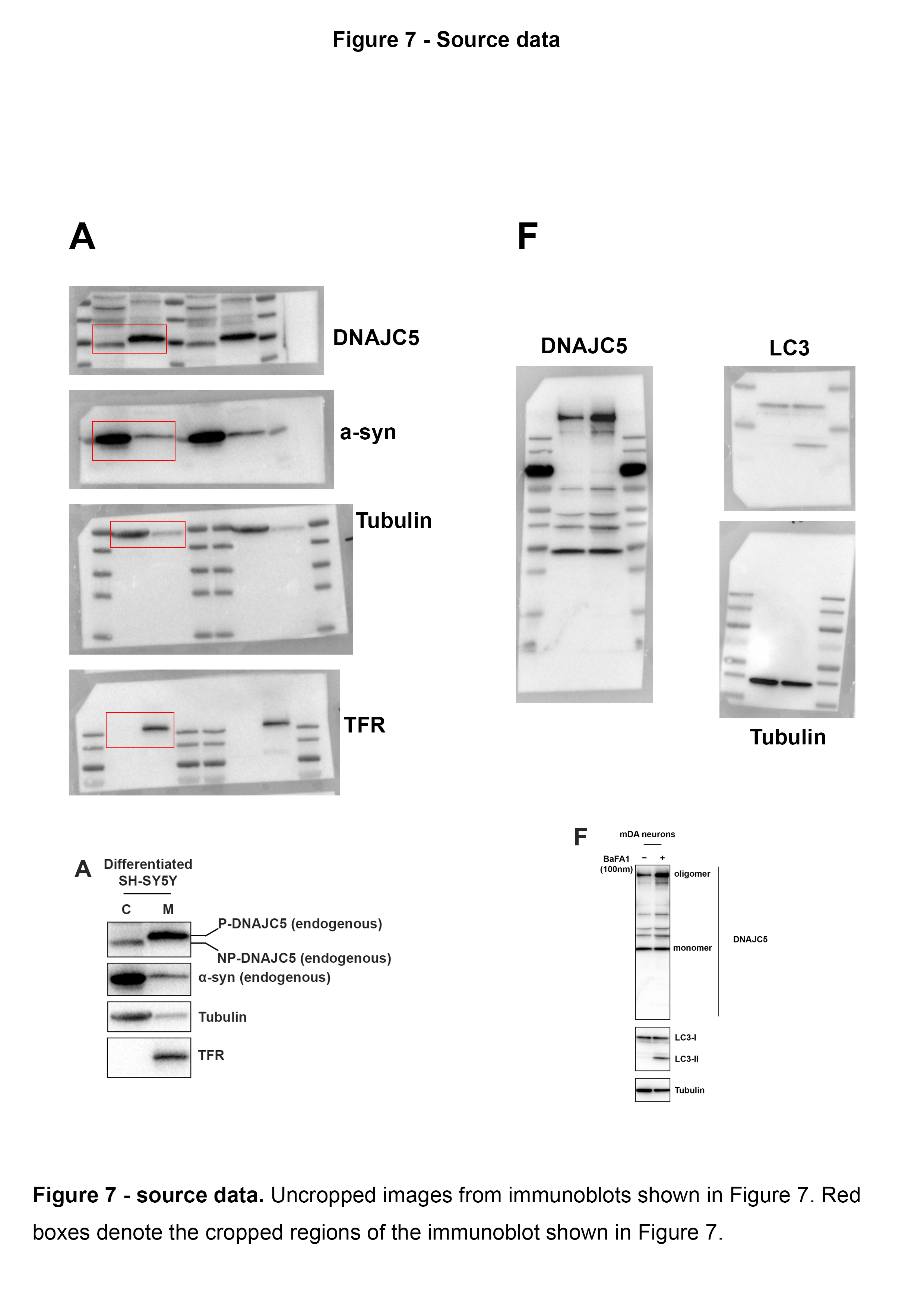

Supplement: Figure 7—source data 1. [file elife-85837-fig7-data1.zip › Figure 7-source data/Figure 7-source data.tif]

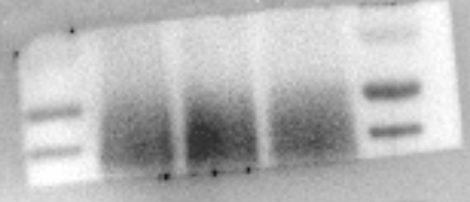

Supplement: Figure 7—figure supplement 1—source data 1. [file elife-85837-fig7-figsupp1-data1.zip › Figure 7-figure supplement 1-source data/Figure 7-figure supplement 1C-4.tif]

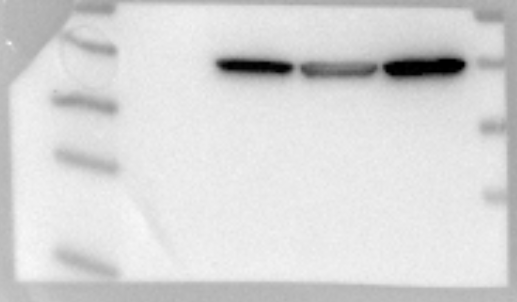

Supplement: Figure 7—figure supplement 1—source data 1. [file elife-85837-fig7-figsupp1-data1.zip › Figure 7-figure supplement 1-source data/Figure 7-figure supplement 1A-4.tif]

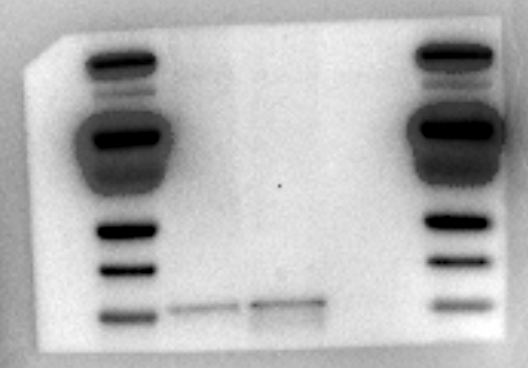

Supplement: Figure 7—figure supplement 1—source data 1. [file elife-85837-fig7-figsupp1-data1.zip › Figure 7-figure supplement 1-source data/Figure 7-figure supplement 1C-2.tif]

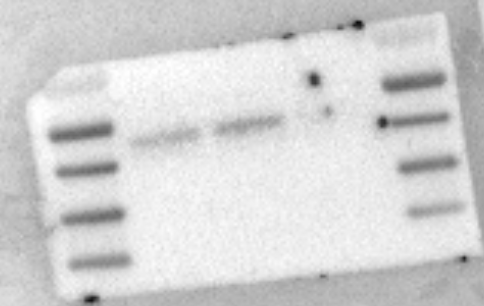

Supplement: Figure 7—figure supplement 1—source data 1. [file elife-85837-fig7-figsupp1-data1.zip › Figure 7-figure supplement 1-source data/Figure 7-figure supplement 1C-3.tif]

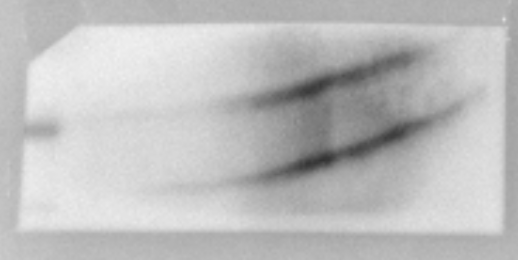

Supplement: Figure 7—figure supplement 1—source data 1. [file elife-85837-fig7-figsupp1-data1.zip › Figure 7-figure supplement 1-source data/Figure 7-figure supplement 1A-1.tif]

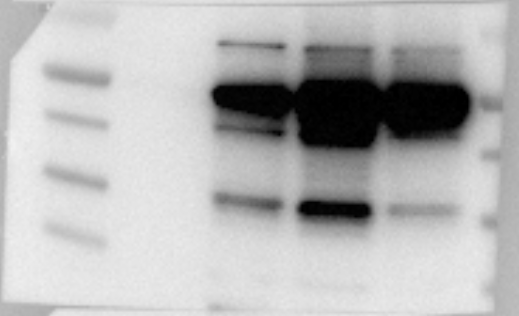

Supplement: Figure 7—figure supplement 1—source data 1. [file elife-85837-fig7-figsupp1-data1.zip › Figure 7-figure supplement 1-source data/Figure 7-figure supplement 1A-3.tif]

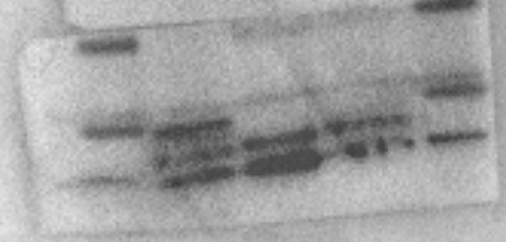

Supplement: Figure 7—figure supplement 1—source data 1. [file elife-85837-fig7-figsupp1-data1.zip › Figure 7-figure supplement 1-source data/Figure 7-figure supplement 1C-1.tif]

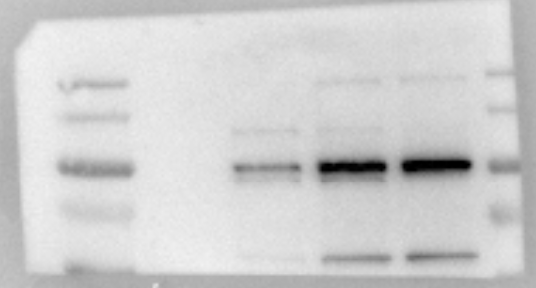

Supplement: Figure 7—figure supplement 1—source data 1. [file elife-85837-fig7-figsupp1-data1.zip › Figure 7-figure supplement 1-source data/Figure 7-figure supplement 1A-2.tif]

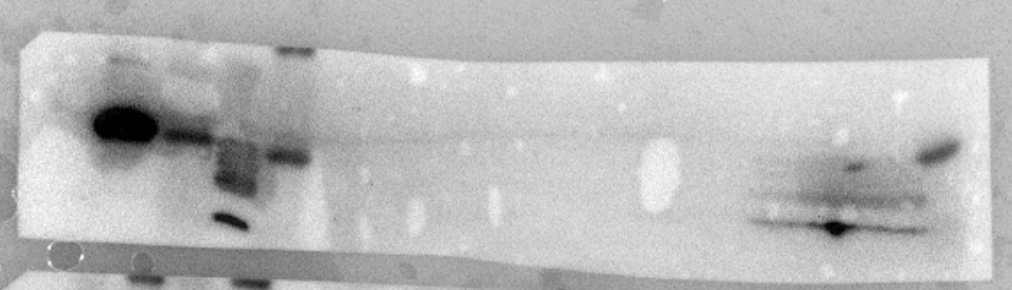

Supplement: Figure 7—figure supplement 1—source data 1. [file elife-85837-fig7-figsupp1-data1.zip › Figure 7-figure supplement 1-source data/Figure 7-figure supplement 1D-1.tif]

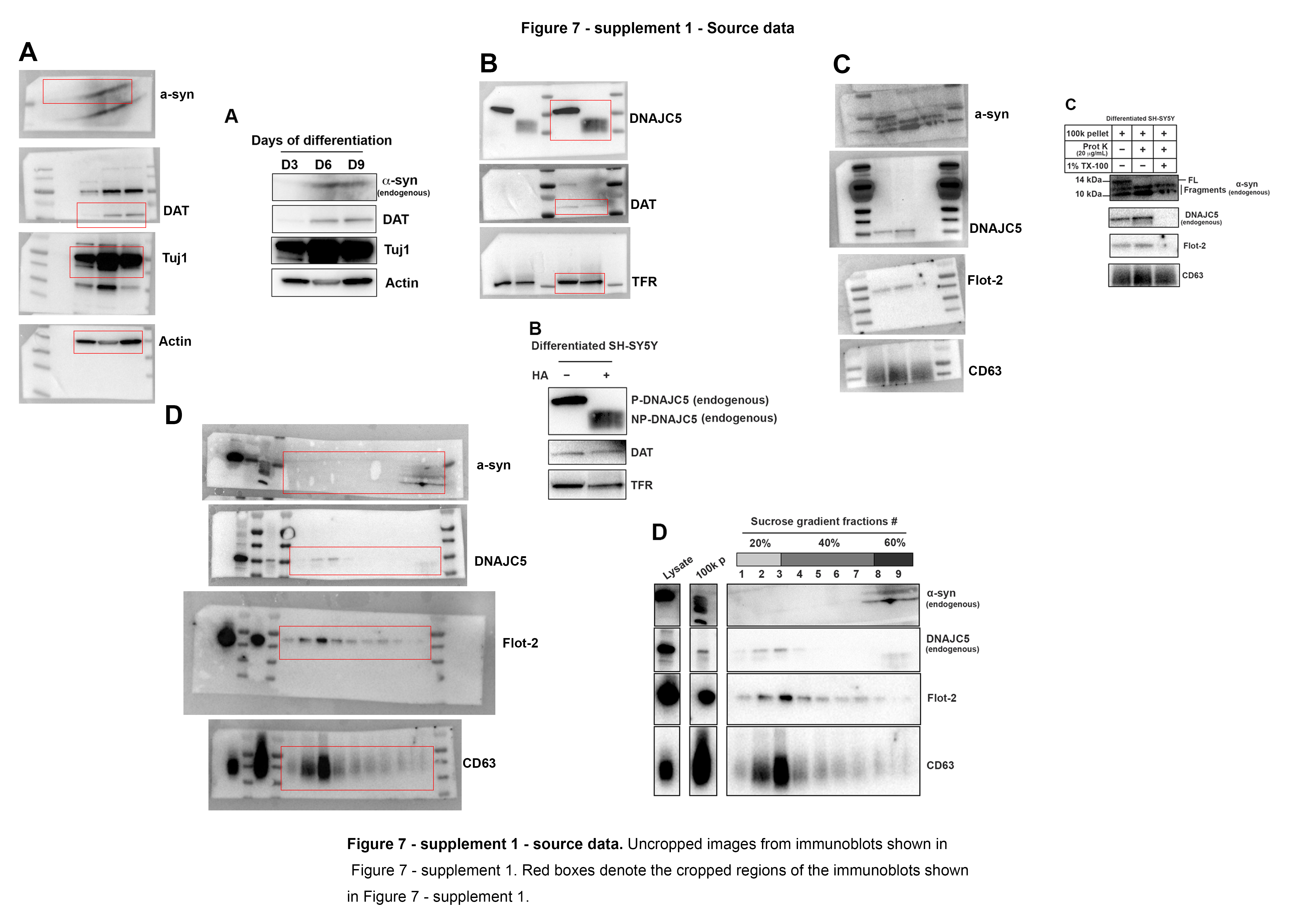

Supplement: Figure 7—figure supplement 1—source data 1. [file elife-85837-fig7-figsupp1-data1.zip › Figure 7-figure supplement 1-source data/Figure 7-figure supplement 1-source data.tif]

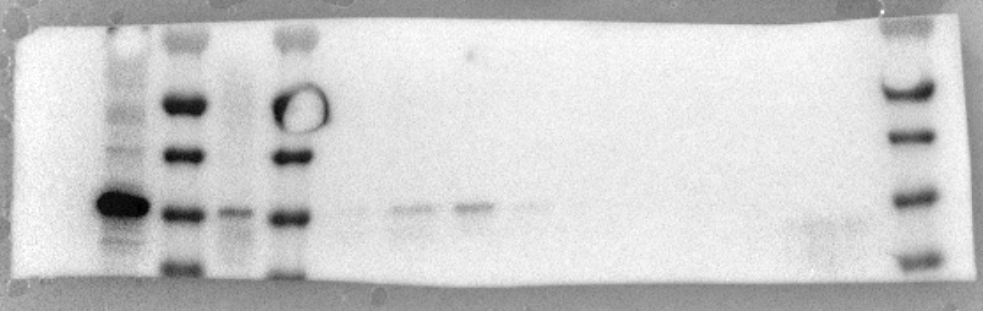

Supplement: Figure 7—figure supplement 1—source data 1. [file elife-85837-fig7-figsupp1-data1.zip › Figure 7-figure supplement 1-source data/Figure 7-figure supplement 1D-2.tif]

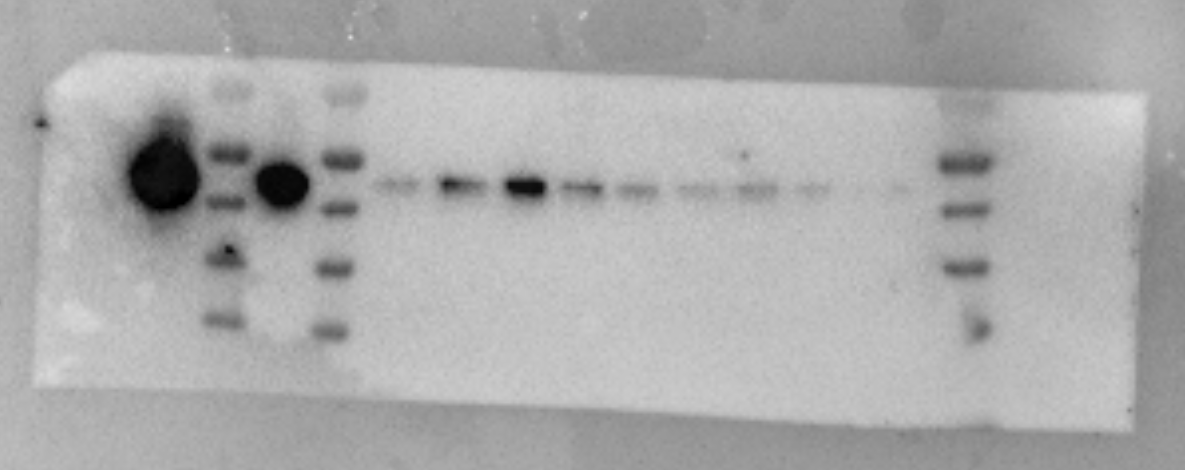

Supplement: Figure 7—figure supplement 1—source data 1. [file elife-85837-fig7-figsupp1-data1.zip › Figure 7-figure supplement 1-source data/Figure 7-figure supplement 1D-3.tif]

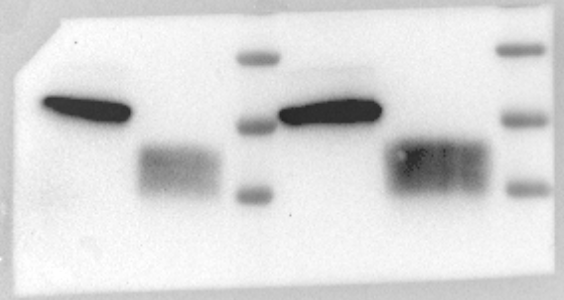

Supplement: Figure 7—figure supplement 1—source data 1. [file elife-85837-fig7-figsupp1-data1.zip › Figure 7-figure supplement 1-source data/Figure 7-figure supplement 1B-1.tif]

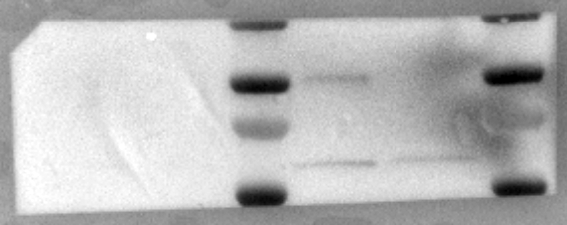

Supplement: Figure 7—figure supplement 1—source data 1. [file elife-85837-fig7-figsupp1-data1.zip › Figure 7-figure supplement 1-source data/Figure 7-figure supplement 1B-2.tif]

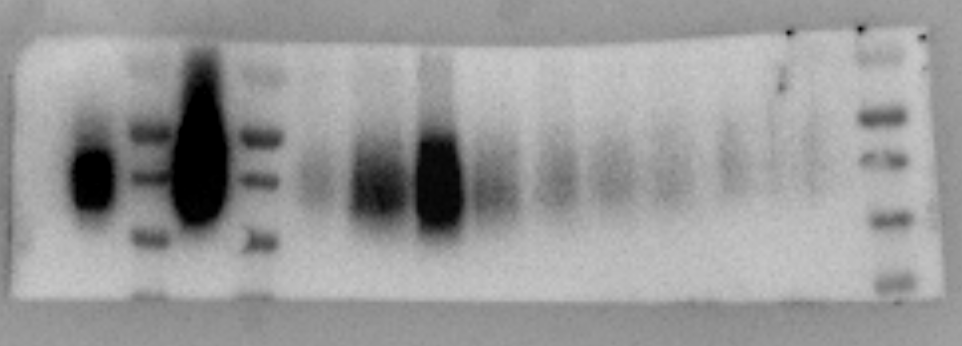

Supplement: Figure 7—figure supplement 1—source data 1. [file elife-85837-fig7-figsupp1-data1.zip › Figure 7-figure supplement 1-source data/Figure 7-figure supplement 1D-4.tif]

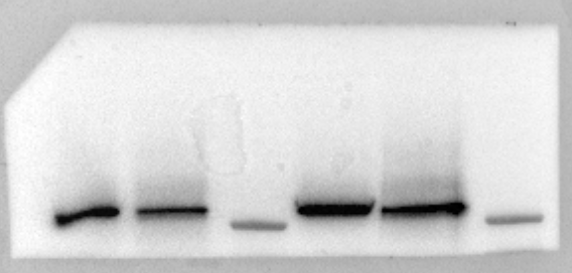

Supplement: Figure 7—figure supplement 1—source data 1. [file elife-85837-fig7-figsupp1-data1.zip › Figure 7-figure supplement 1-source data/Figure 7-figure supplement 1B-3.tif]

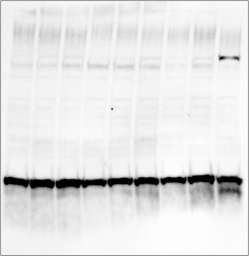

Supplement: Figure 7—figure supplement 2—source data 1. [file elife-85837-fig7-figsupp2-data1.zip › Figure 7-figure supplement 2-source data/Figure 7-figure supplement 2B-1.tif]

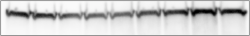

Supplement: Figure 7—figure supplement 2—source data 1. [file elife-85837-fig7-figsupp2-data1.zip › Figure 7-figure supplement 2-source data/Figure 7-figure supplement 2B-3.tif]

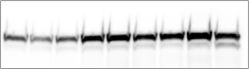

Supplement: Figure 7—figure supplement 2—source data 1. [file elife-85837-fig7-figsupp2-data1.zip › Figure 7-figure supplement 2-source data/Figure 7-figure supplement 2B-2.tif]

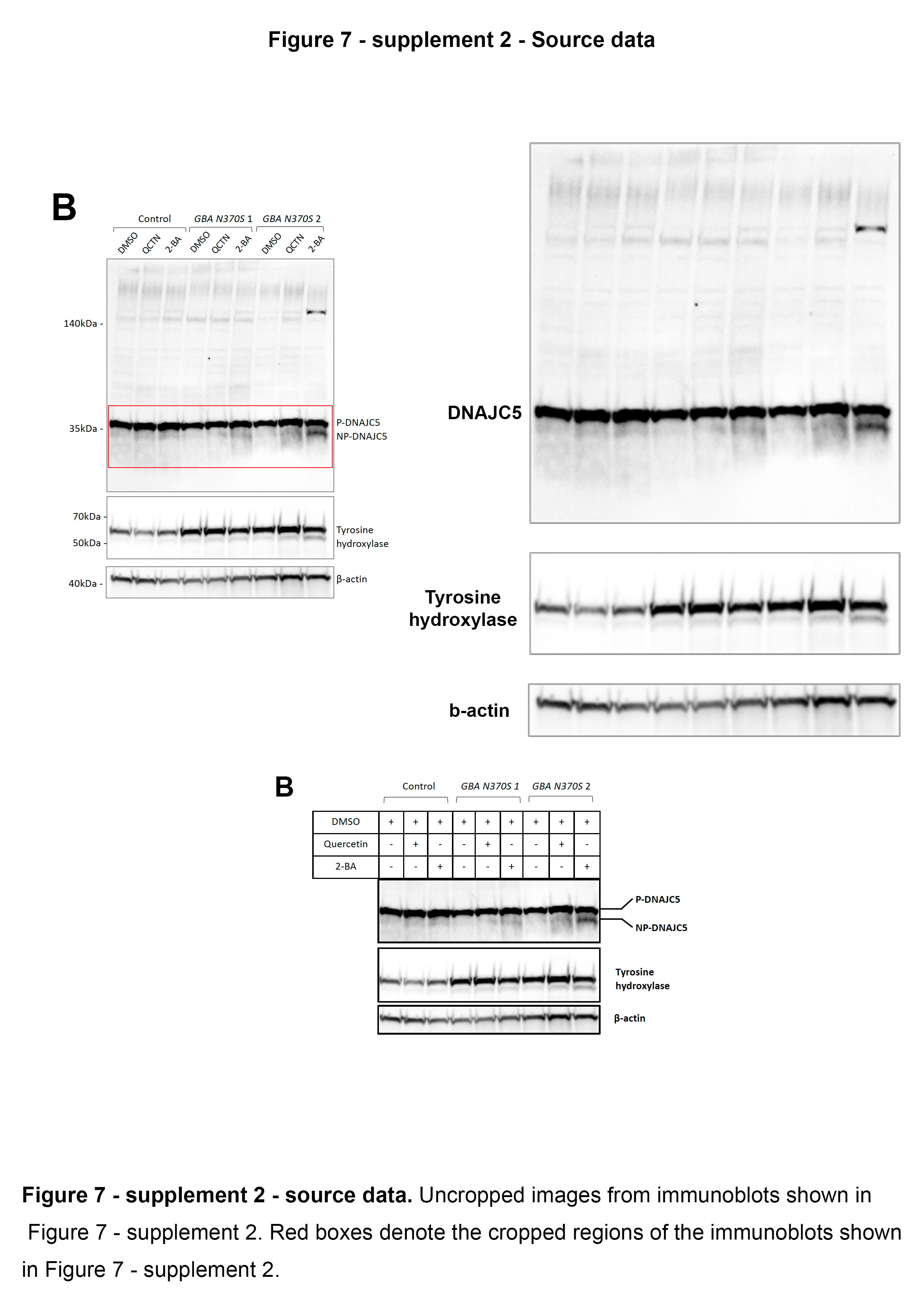

Supplement: Figure 7—figure supplement 2—source data 1. [file elife-85837-fig7-figsupp2-data1.zip › Figure 7-figure supplement 2-source data/Figure 7-figure supplement 2-source data.tif]

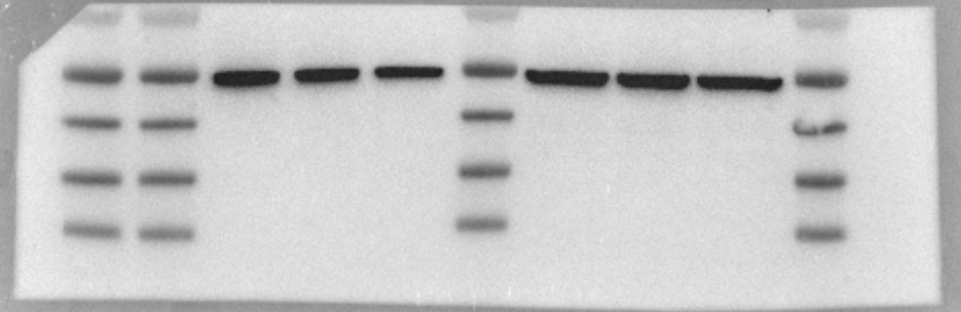

Supplement: Figure 7—figure supplement 3—source data 1. [file elife-85837-fig7-figsupp3-data1.zip › Figure 7-figure supplement 3-source data/Figure 7-figure supplement 3A-2.tif]

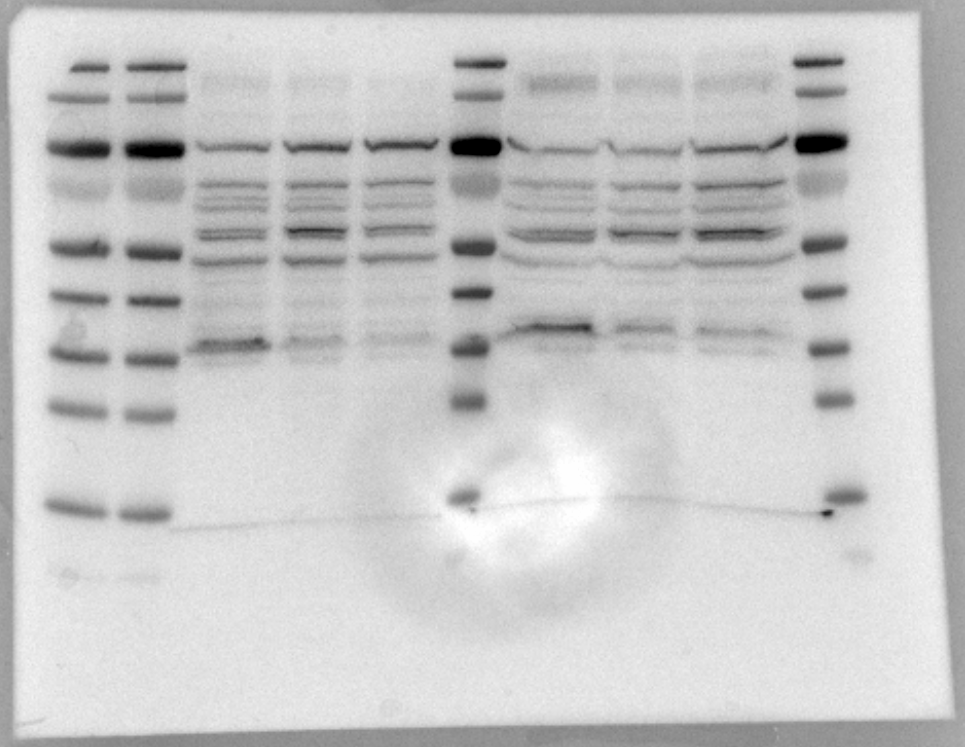

Supplement: Figure 7—figure supplement 3—source data 1. [file elife-85837-fig7-figsupp3-data1.zip › Figure 7-figure supplement 3-source data/Figure 7-figure supplement 3A-1.tif]

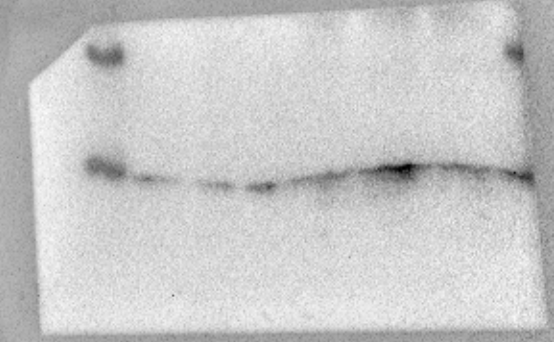

Supplement: Figure 7—figure supplement 3—source data 1. [file elife-85837-fig7-figsupp3-data1.zip › Figure 7-figure supplement 3-source data/Figure 7-figure supplement 3B-2.tif]

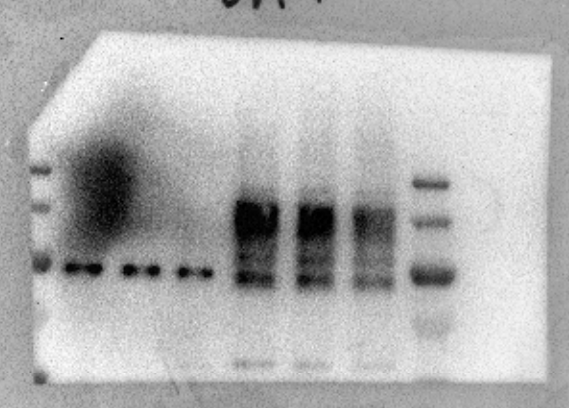

Supplement: Figure 7—figure supplement 3—source data 1. [file elife-85837-fig7-figsupp3-data1.zip › Figure 7-figure supplement 3-source data/Figure 7-figure supplement 3B-3.tif]

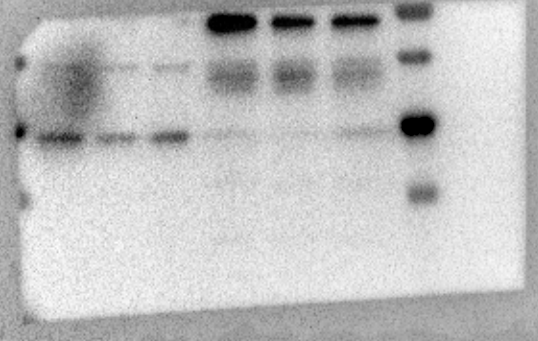

Supplement: Figure 7—figure supplement 3—source data 1. [file elife-85837-fig7-figsupp3-data1.zip › Figure 7-figure supplement 3-source data/Figure 7-figure supplement 3B-1.tif]

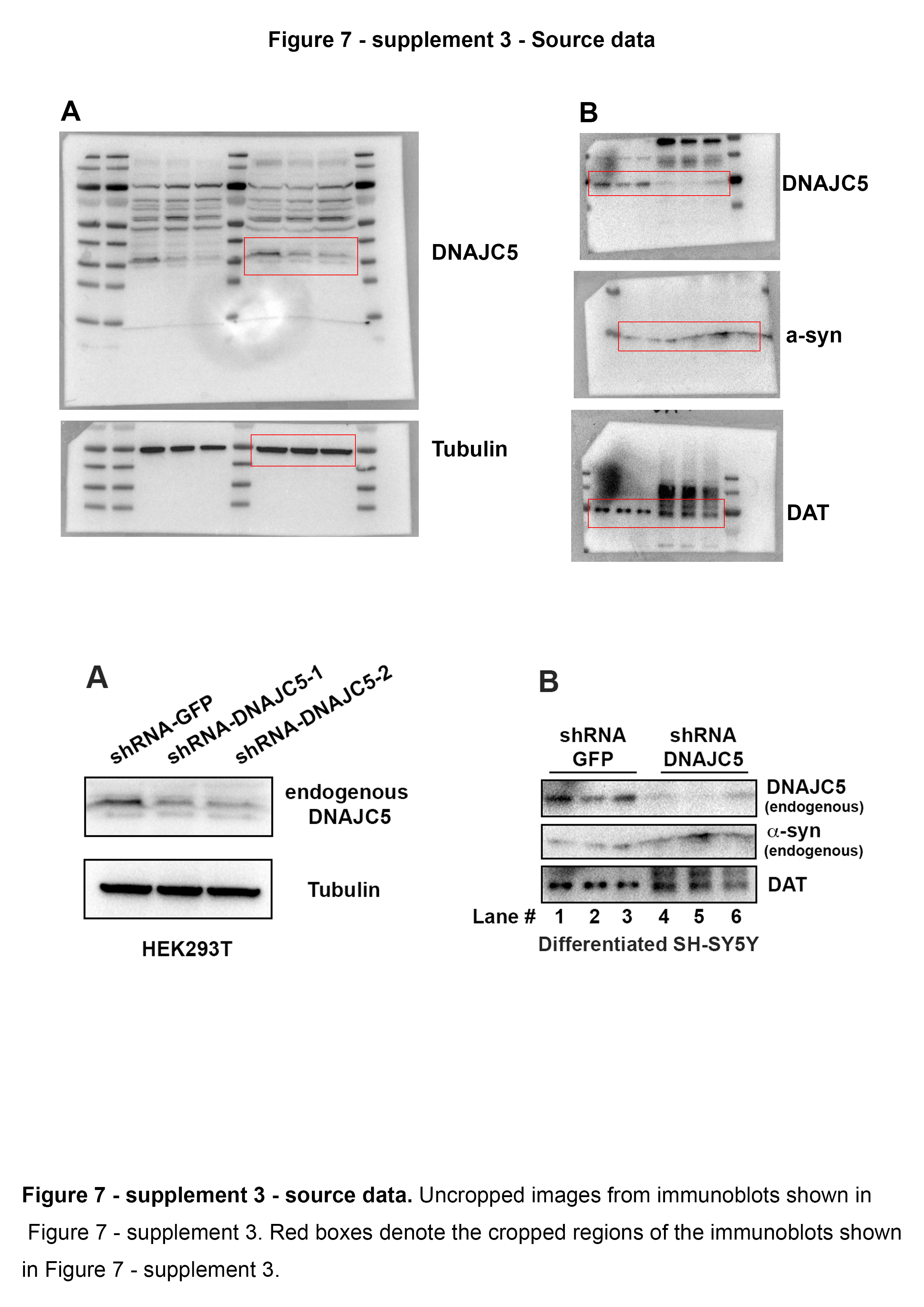

Supplement: Figure 7—figure supplement 3—source data 1. [file elife-85837-fig7-figsupp3-data1.zip › Figure 7-figure supplement 3-source data/Figure 7-figure supplement 3-source data.tif]

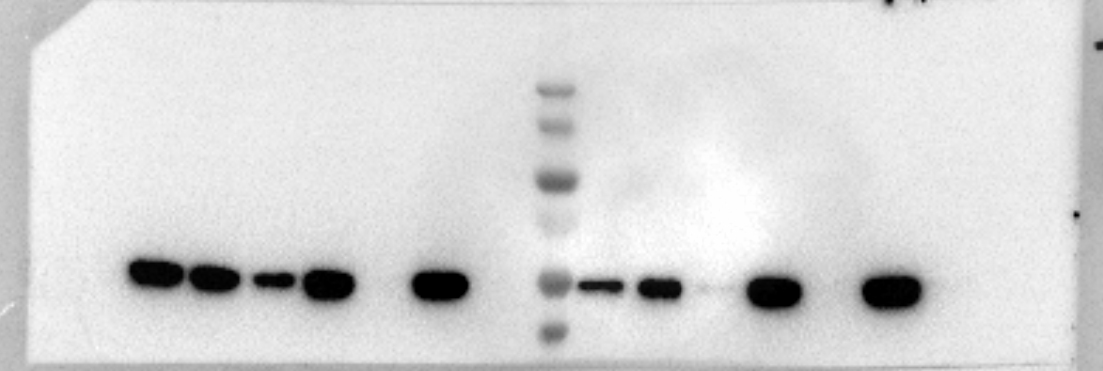

Supplement: Figure 7—figure supplement 4—source data 1. [file elife-85837-fig7-figsupp4-data1.zip › Figure 7-figure supplement 4-source data/Figure 7-figure supplement 4B-2.tif]

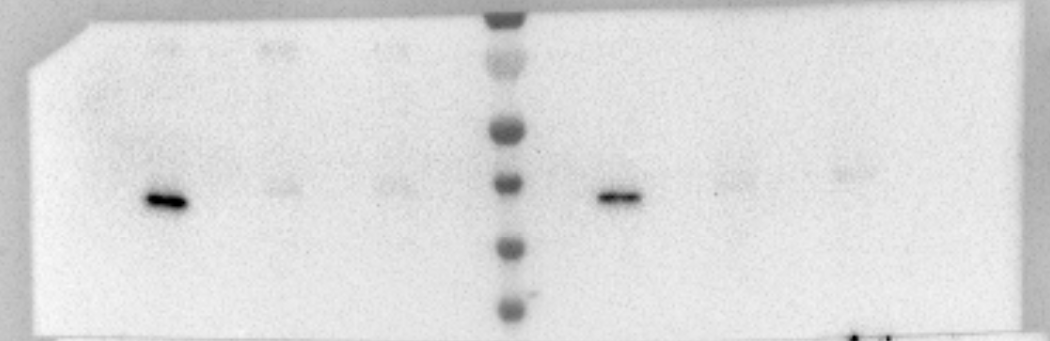

Supplement: Figure 7—figure supplement 4—source data 1. [file elife-85837-fig7-figsupp4-data1.zip › Figure 7-figure supplement 4-source data/Figure 7-figure supplement 4B-1.tif]

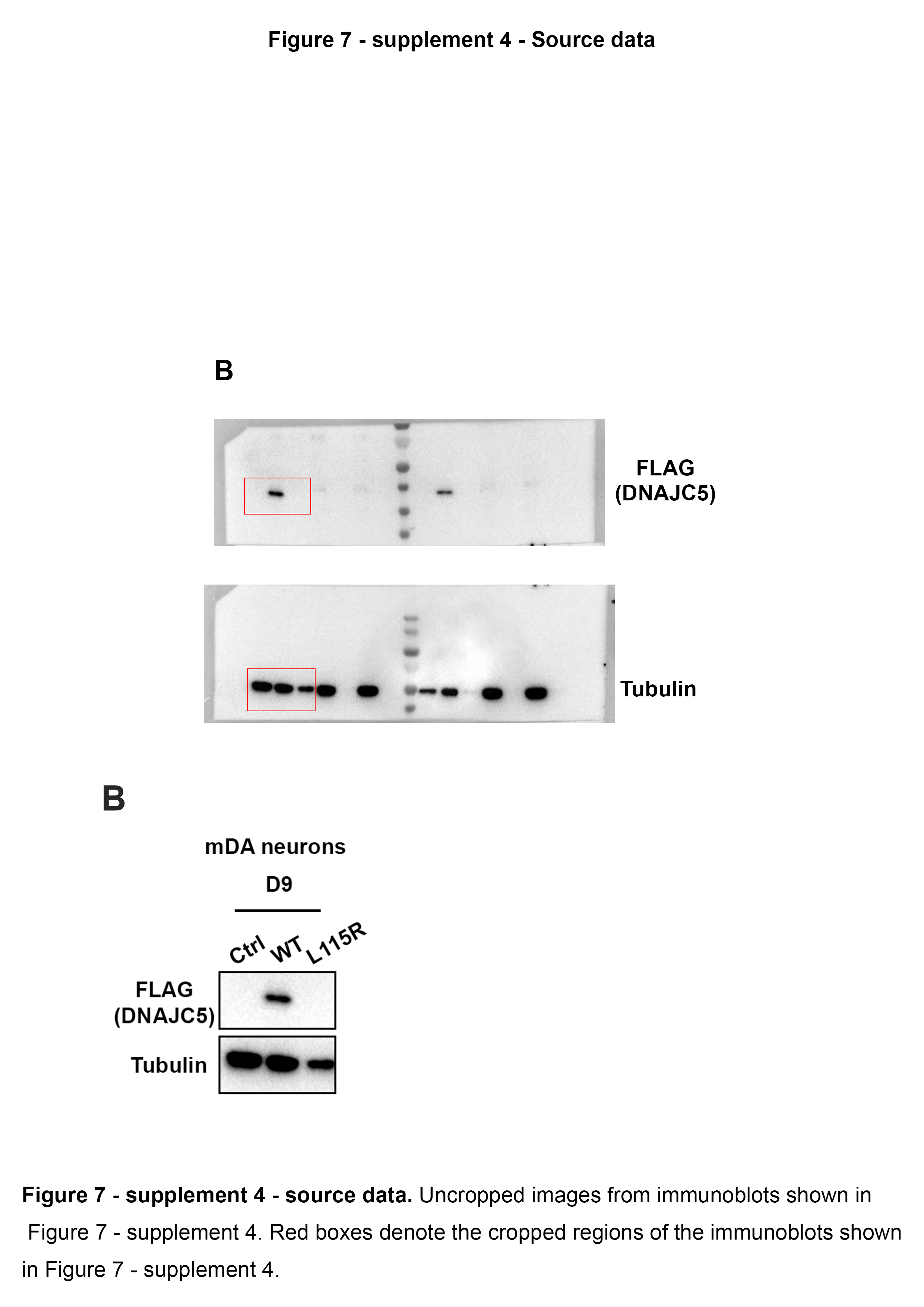

Supplement: Figure 7—figure supplement 4—source data 1. [file elife-85837-fig7-figsupp4-data1.zip › Figure 7-figure supplement 4-source data/Figure 7-figure supplement 4-source data.tif]

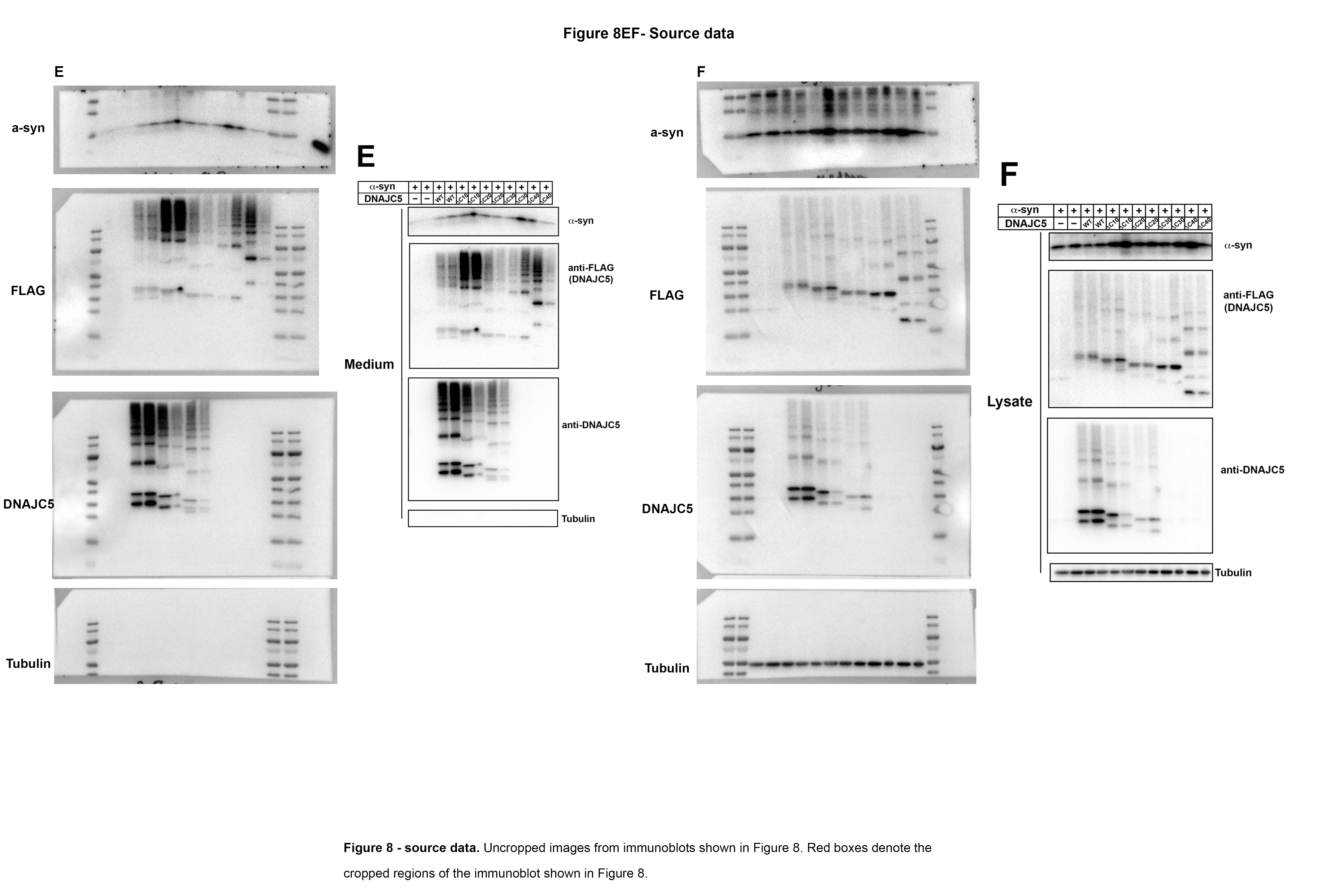

Supplement: Figure 8—source data 1. [file elife-85837-fig8-data1.zip › Figure 8-source data/Figure 8EF-source data.tif]

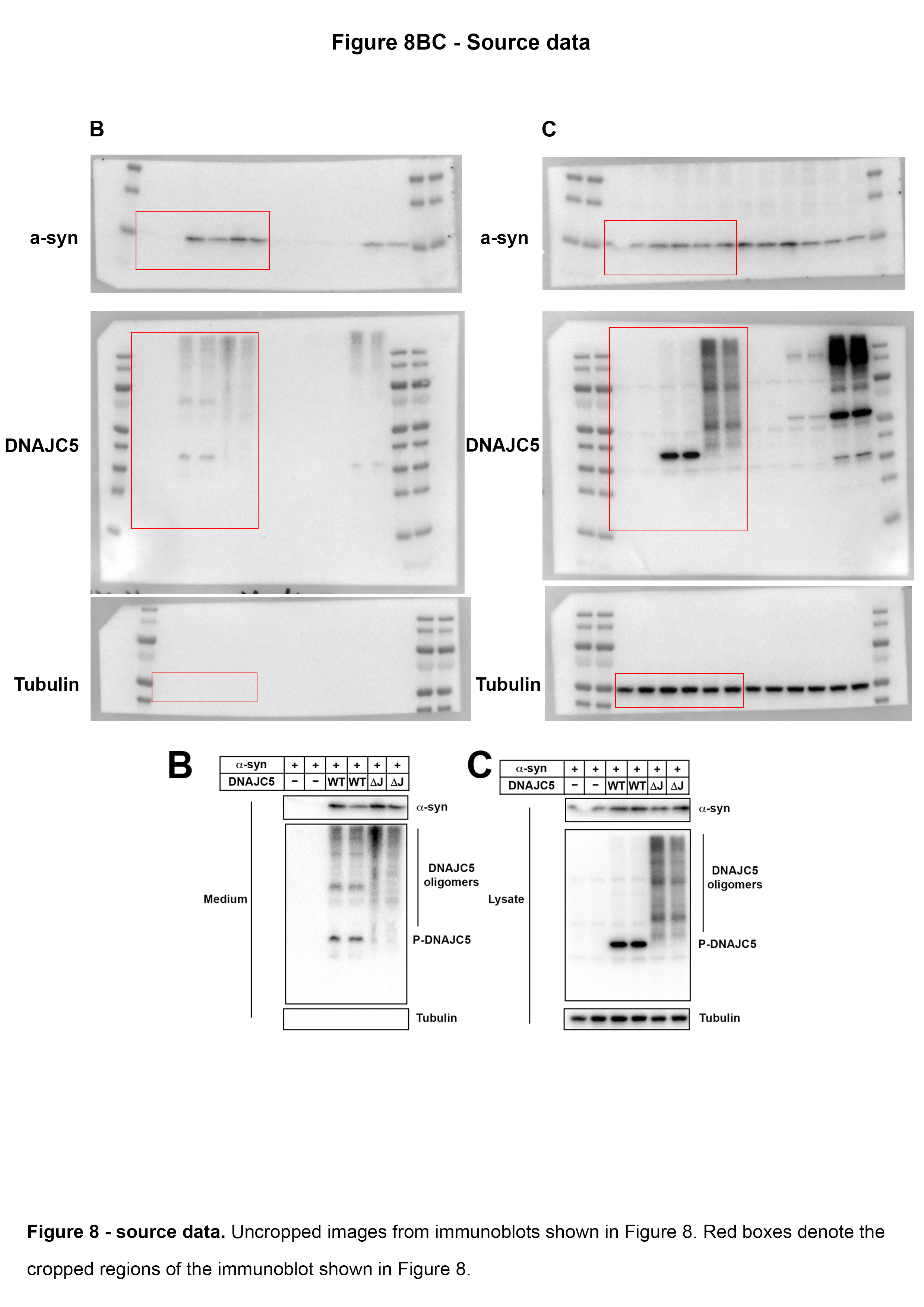

Supplement: Figure 8—source data 1. [file elife-85837-fig8-data1.zip › Figure 8-source data/Figure 8BC-source data.tif]

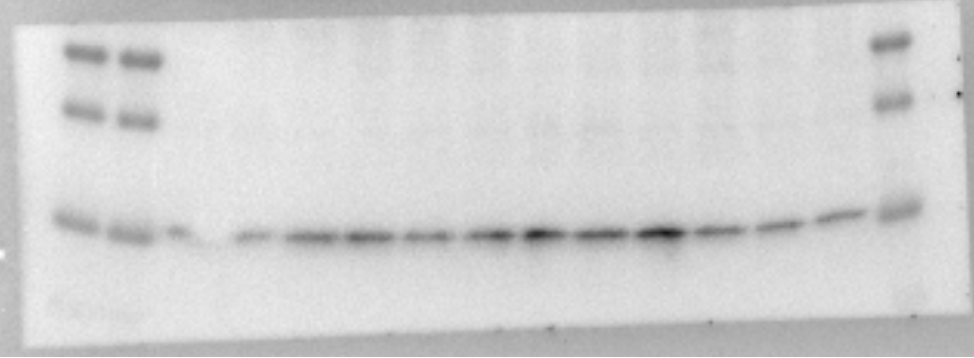

Supplement: Figure 8—source data 1. [file elife-85837-fig8-data1.zip › Figure 8-source data/Figure 8C-1.tif]

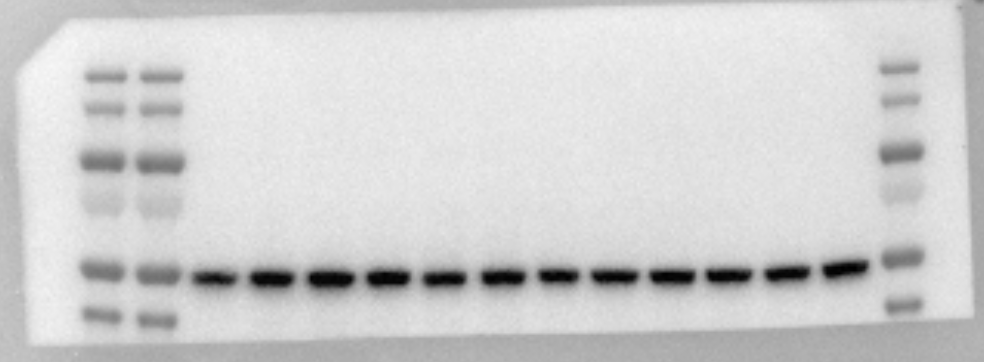

Supplement: Figure 8—source data 1. [file elife-85837-fig8-data1.zip › Figure 8-source data/Figure 8C-3.tif]

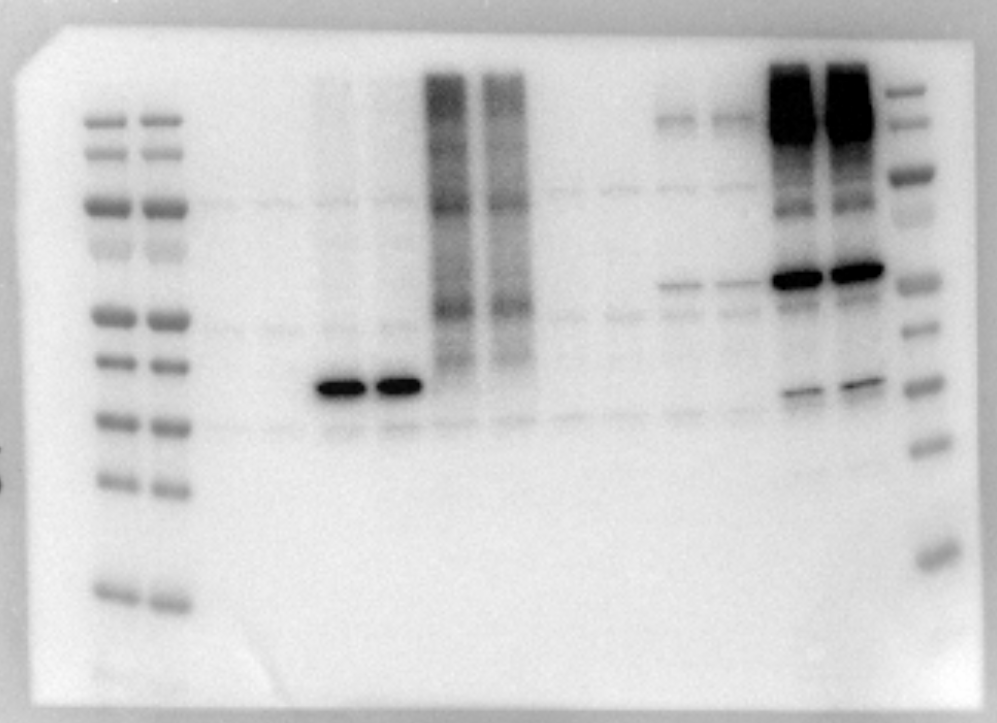

Supplement: Figure 8—source data 1. [file elife-85837-fig8-data1.zip › Figure 8-source data/Figure 8C-2.tif]

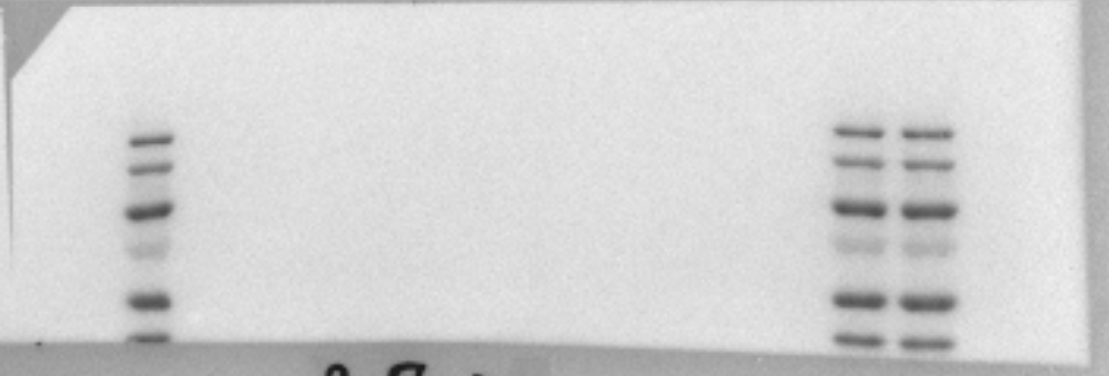

Supplement: Figure 8—source data 1. [file elife-85837-fig8-data1.zip › Figure 8-source data/Figure 8E-4.tif]

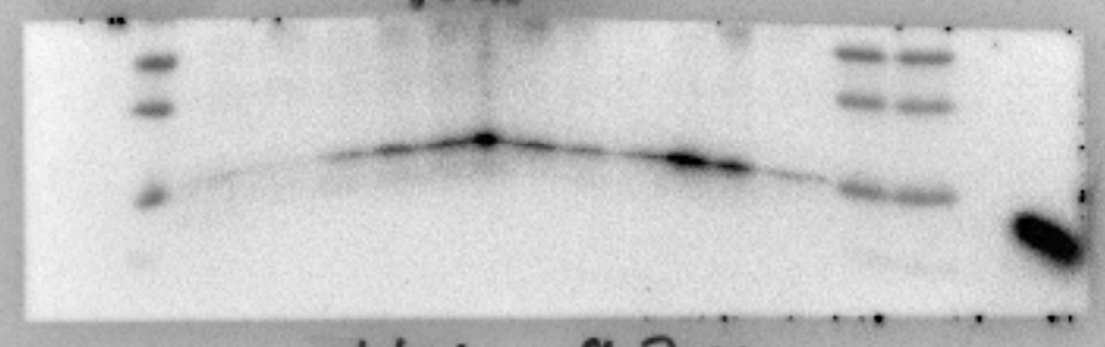

Supplement: Figure 8—source data 1. [file elife-85837-fig8-data1.zip › Figure 8-source data/Figure 8E-1.tif]

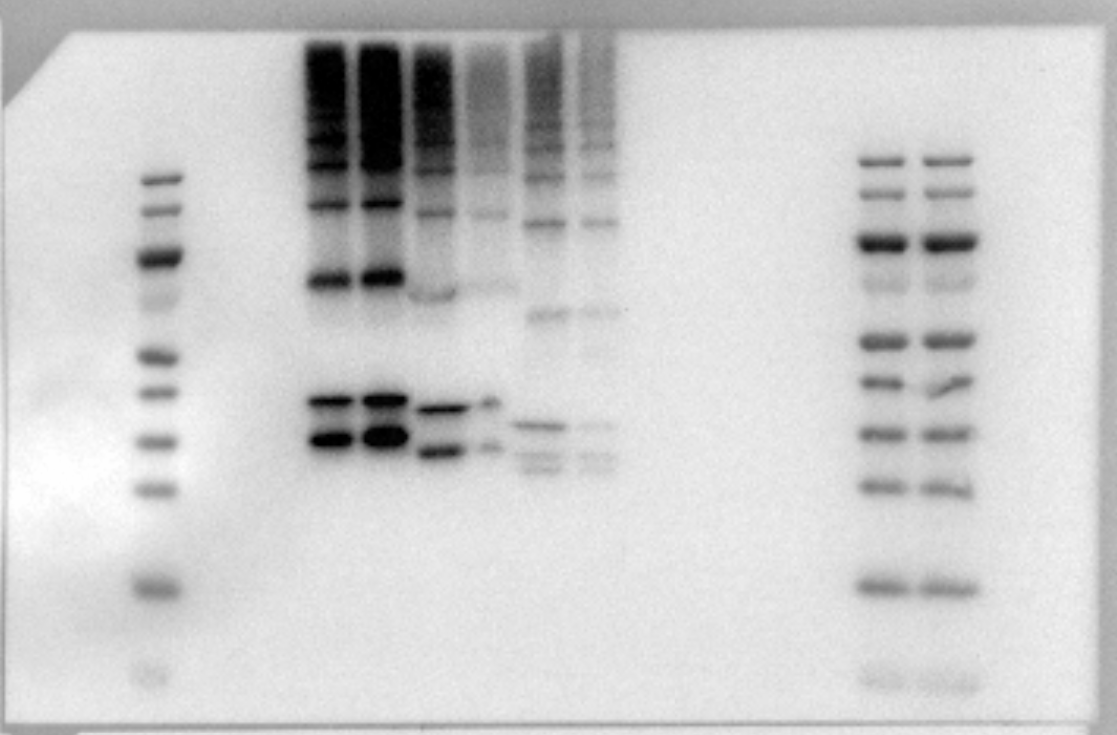

Supplement: Figure 8—source data 1. [file elife-85837-fig8-data1.zip › Figure 8-source data/Figure 8E-3.tif]

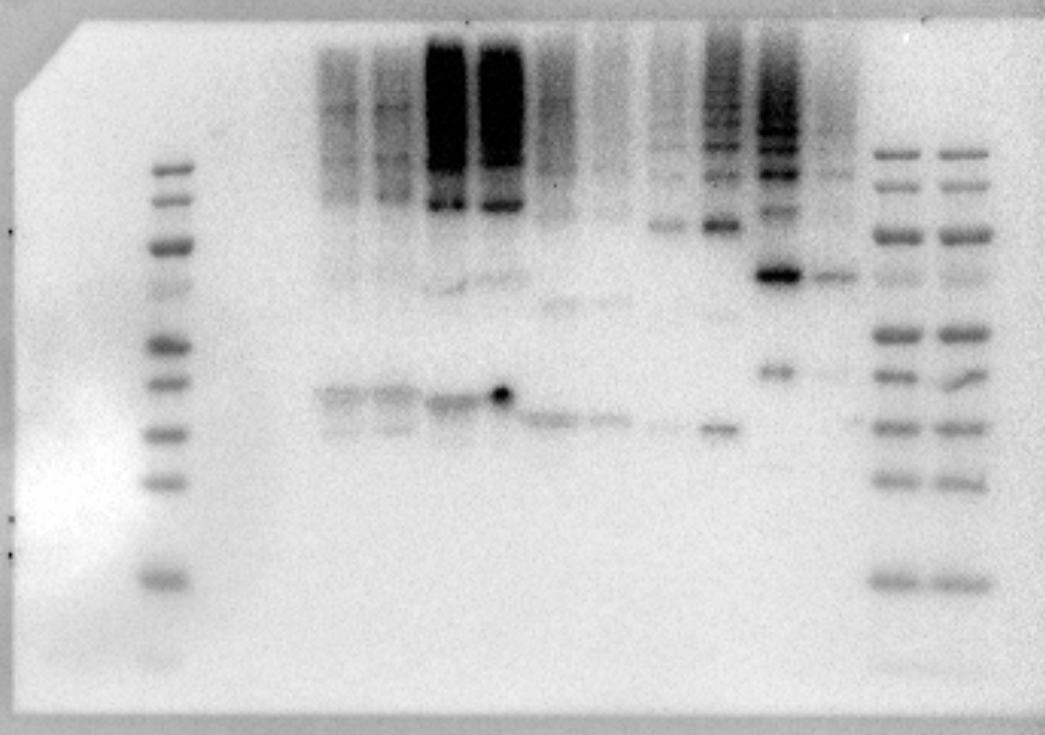

Supplement: Figure 8—source data 1. [file elife-85837-fig8-data1.zip › Figure 8-source data/Figure 8E-2.tif]

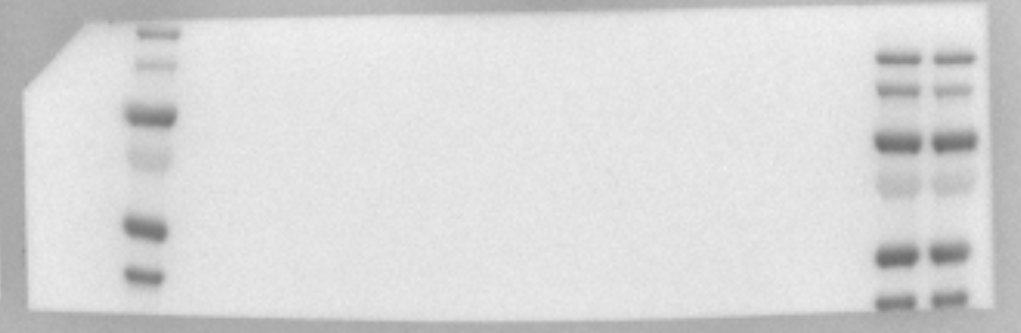

Supplement: Figure 8—source data 1. [file elife-85837-fig8-data1.zip › Figure 8-source data/Figure 8B-3.tif]

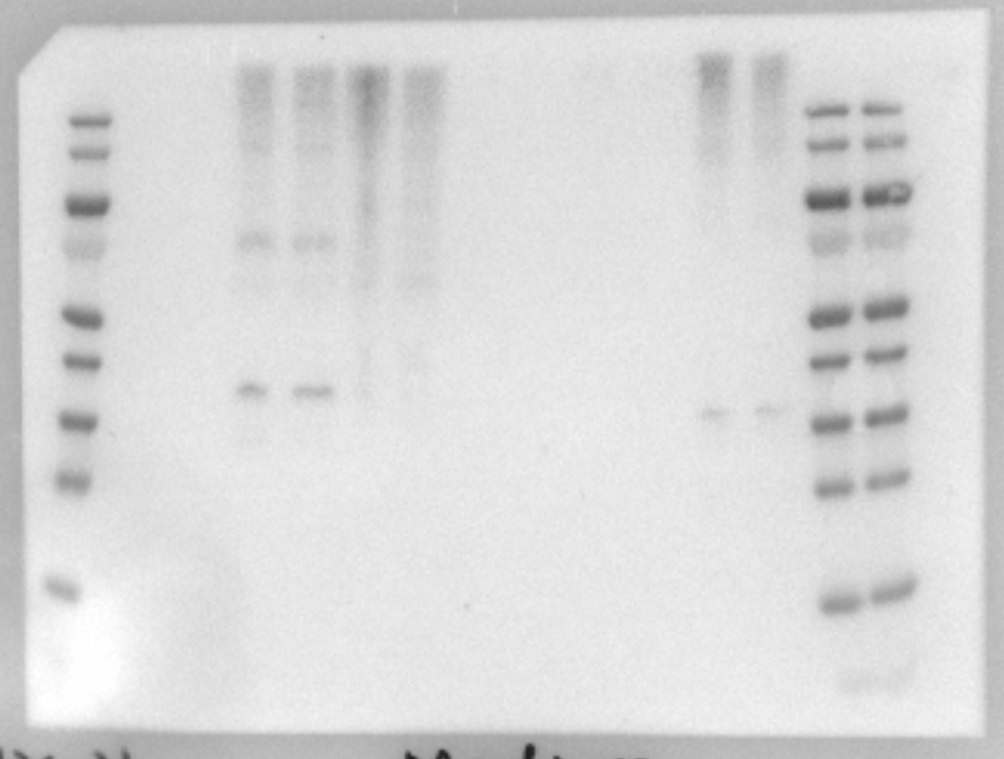

Supplement: Figure 8—source data 1. [file elife-85837-fig8-data1.zip › Figure 8-source data/Figure 8B-2.tif]

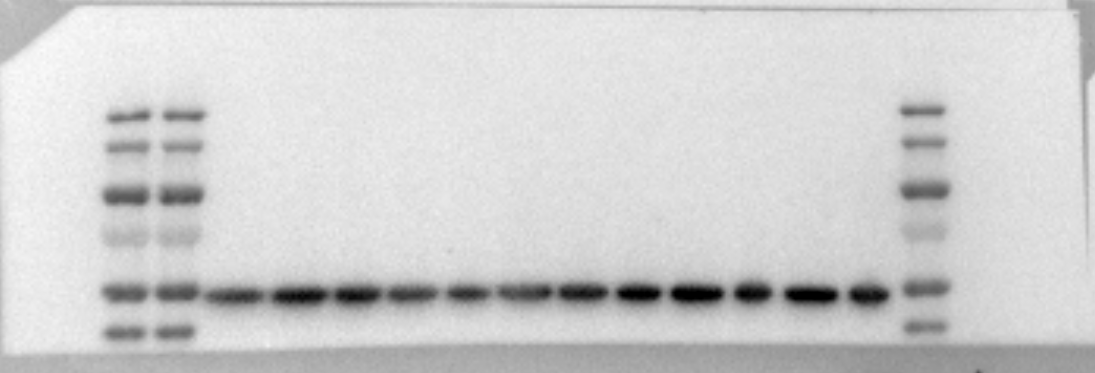

Supplement: Figure 8—source data 1. [file elife-85837-fig8-data1.zip › Figure 8-source data/Figure 8F-4.tif]

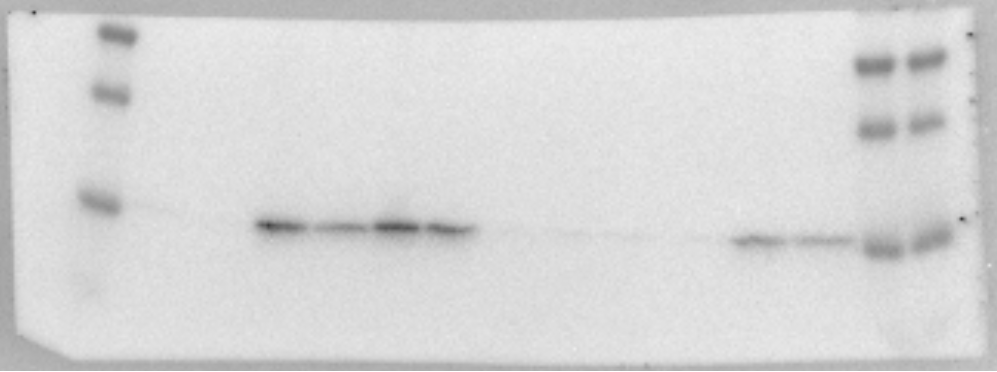

Supplement: Figure 8—source data 1. [file elife-85837-fig8-data1.zip › Figure 8-source data/Figure 8B-1.tif]

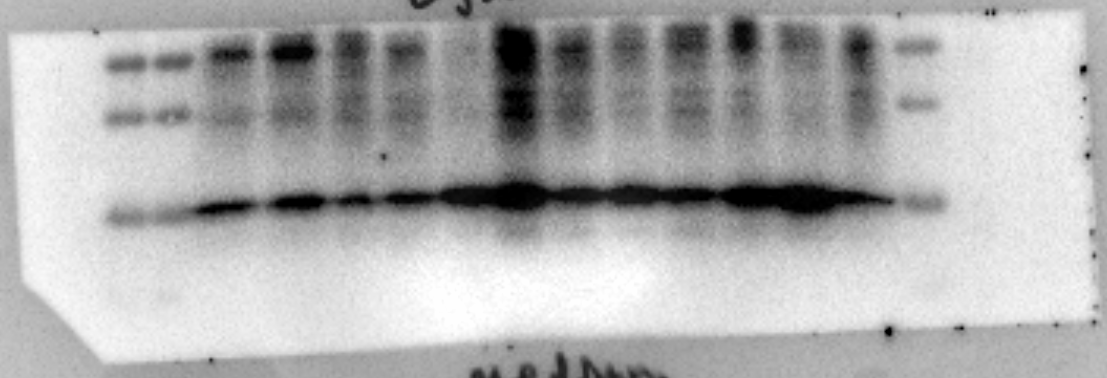

Supplement: Figure 8—source data 1. [file elife-85837-fig8-data1.zip › Figure 8-source data/Figure 8F-1.tif]

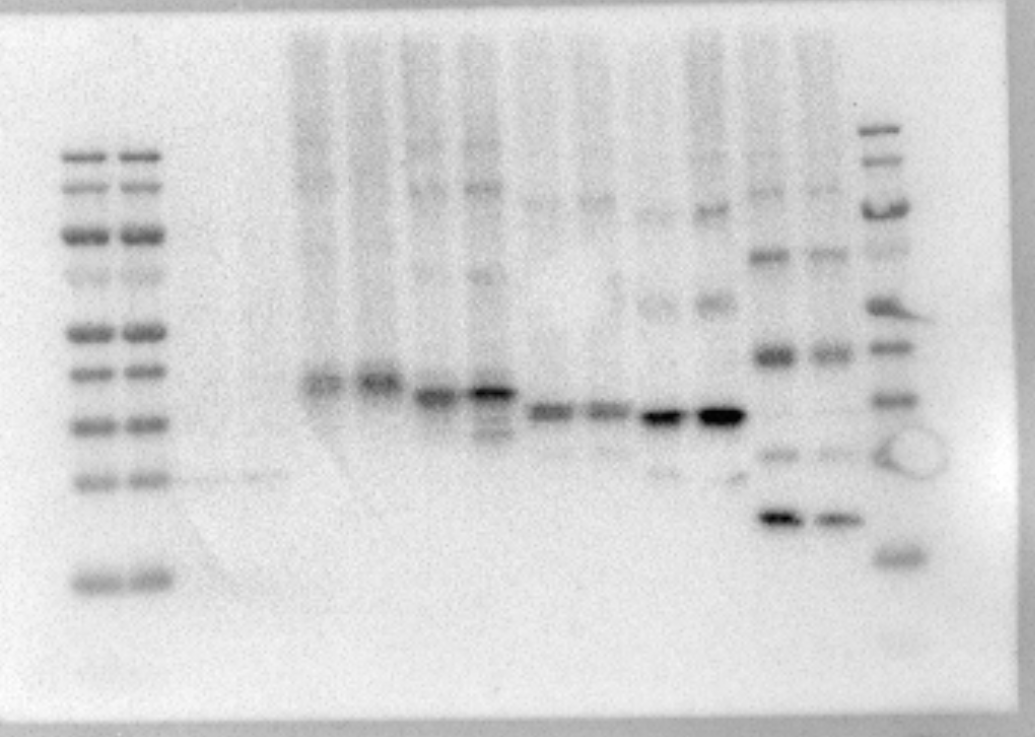

Supplement: Figure 8—source data 1. [file elife-85837-fig8-data1.zip › Figure 8-source data/Figure 8F-2.tif]

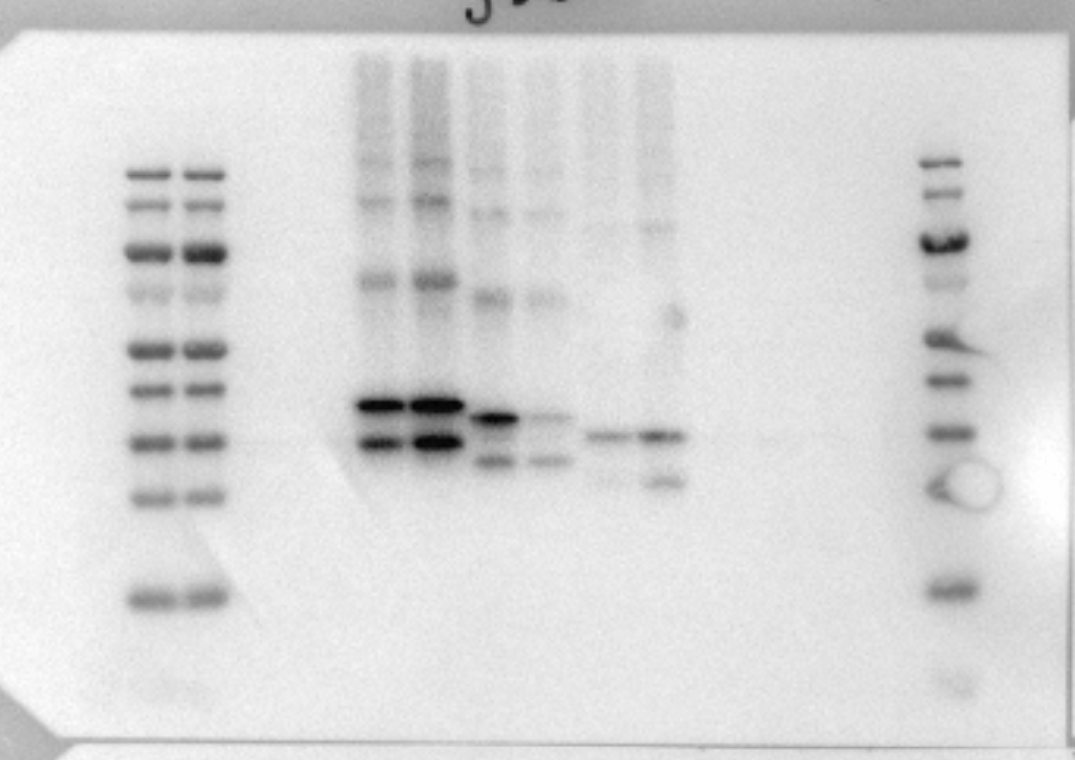

Supplement: Figure 8—source data 1. [file elife-85837-fig8-data1.zip › Figure 8-source data/Figure 8F-3.tif]
